# 7-day patterns in Black-White segregation in 49 metropolitan areas

**DOI:** 10.1038/s41598-024-56257-1

**Published:** 2024-03-20

**Authors:** Joanna Chae

**Affiliations:** Columbia University in the City of New York, Sociology, New York, NY 10027 USA

**Keywords:** Population dynamics, Urban ecology

## Abstract

While residential segregation is a persistent attribute of metropolitan areas, recent studies find segregation levels fluctuate throughout the day, reaching their lowest levels during daytime hours. This paper shows hourly variations in Black-White segregation from Monday through Sunday for the top 49 most populated metropolitan areas with Global Positioning System (GPS) data collected from mobile phones from October 2018. I find that segregation levels are higher on average over weekends compared to that of weekdays. I use models to identify the characteristics of neighborhoods with higher levels of segregation on weekends, which include all demographic variables and nearly a third of 35 sectors of businesses and organizations, such as retail, personal care, and religious organizations. I also find more than a third of the sectors are associated with higher levels of segregation during business hours on weekdays, including academic institutions, health care, manufacturing, and financial institutions. Findings from this paper display the significance in the distinction between weekdays and weekends with where people spend their time and how this relates to racial segregation. Specifically, Black people, on average, stay in their home census tracts and visit non-White neighborhoods for organizational resources more so than White people. Significant patterns of associations between racial segregation and the majority of businesses demonstrate the salience of race for more industries than previously understood.

## Introduction

Racial residential segregation has been described as the structural linchpin through which racial stratification persists in America^1,2^. Racial segregation has been found to perpetuate disadvantages among racial minorities, which encompass higher rates of poverty^3,4^, poorer educational outcomes^5,6^, political marginalization^7^, racial hostility^8^, greater exposure to crime^9,10^, and poorer health outcomes^11,12^. This study specifically focuses on Black-White segregation because it is most severe compared to other forms of racial segregation^13^. While Black-White residential segregation has declined across many decades^14^, as of 2010, one third of Black people live in hypersegregated metropolitan areas^15^.

Scholars have studied racial segregation in a variety of contexts, such as schools and workplaces, to understand whether segregation levels as measured by residential neighborhoods reflect the segregation levels people experience. Global positioning system (GPS) data has made it possible to measure segregation levels of a wider variety of places, including restaurants, outdoor spaces, museums, and pharmacies^16–18^. These studies find that while people spend time in less segregated spaces compared to that of residential neighborhoods, one’s race, income, and political affiliations are found to be correlated with where they travel to for everyday activities^16–21^. This study builds on prior work by demonstrating how racial segregation is associated with a comprehensive list of industries that people visit for not only leisure, but also for work to demonstrate the salience of race in the local spaces in which businesses are embedded.

Recent studies also show temporal variation in segregation levels. With mobile phone location data, Athey et al.^17^ show hourly trends throughout an average, 24-h day and find that racial segregation, between Whites and Non-Whites, is lower during the day than in the evening, which can be attributed to the high volume of travel people make in large metropolitan areas^22^.

This study expands on prior work by showing how hourly trends differ throughout a 7-day week to examine how segregation levels change from weekdays to weekends and whether differences are found during daytime hours when travel is more frequent. The distinction between weekdays and weekends is important for several reasons. First, changes in hours dedicated for work may contribute to dissimilar segregation trends between weekdays and weekends. According to the American Time Use Survey (ATUS), in 2018, 54.2% of the population worked on an average weekday, while 20.4% of the population worked on an average weekend. The average number of worked hours is also remarkably less over weekends. In addition, according to the Work Schedules and Work at Home survey, a special supplement to the Current Population Survey, more Black people take evening or night shifts, yet they work on weekends at similar rates as White people^23^. Although declines in Black-White segregation between occupations and schools have stalled since the 1980s^24–26^, neighborhoods where people work are less segregated than where people live^27^. Racial integration among employees are found in occupations with low average earnings with retail trade being the most integrated sector^25,28^. Hence, segregation levels are expected to be lower on weekdays due to longer hours spent for work and greater racial diversity in the neighborhoods where people work.

Second, changes in hours dedicated for leisure is another factor that could contribute to dissimilar segregation trends between weekdays and weekends. The ATUS reports more time is spent socializing, participating in recreational activities, and housework on weekends. While less is known about differences in how Black and White people spend their weekends, prior work suggests differences in how leisure time is spent by race. For example, White people have been found to be more physically active during leisure time^29,30^, as well as visit museums and performances, and attend extracurricular and cultural classes more frequently than Black people^31,32^. Given these findings, segregation levels could increase on days with more leisure time, as on weekends.

Third, distinguishing weekdays from weekends in addition to distinguishing hours in which businesses, in general, are open and closed, is important to consider when estimating segregation levels by place. Leisure and hospitality, wholesale and retail trade, and transportation and utilities are the only sectors where more than 20% of its wage and salary workers have alternate-shift schedules, which includes working evenings and weekends^23^. Lower percentages of alternate-shift schedules suggest limited business hours beyond 9 AM to 5 PM, Monday through Friday. Hence, comparisons in associations by weekdays and weekends during daytime hours can demonstrate how businesses contribute to the racial segregation of their local environments.

Taken together, this study makes three important contributions. First, I build on prior work by showing how Black-White segregation fluctuates across the week, with a focus on how levels change between weekdays and weekends. Second, I demonstrate how these fluctuations are attributable to businesses by narrowing in on the days and hours when businesses are most active. Third, I describe whether differences in segregation levels are attributed to the presence of Black or White people, and whether specific categories of census tracts, as classified by percentages of Black or White residents, contribute more towards hourly racial segregation.

## Data

### Mobile phone location data

I use proprietary, anonymized mobile phone location data sold by Fysical Labs from October 2018 for the 49 most populous metropolitan areas in the US. Data points were generated whenever devices move 100 m, stop moving for 5 min, or detect an iBeacon. Data were collected from several dozen applications that use location services, such as weather, navigation and social media. These conditions generate unweighted, panel data that include unique IDs for mobile phones, and longitudes and latitudes of the locations mobile phones are found. The data include 15,037,449 unique devices. More details on the sample can be found in Sections B and C of the Appendix.

### Additional data sources

I use three sources of data to examine the characteristics of census tracts that contribute more towards Black-White segregation. I use the 2018 American Community Survey (ACS) in two ways. First, I use data by census block groups to impute demographic information for mobile phone owners. Second, I use data by census tracts to compute residential levels, with which I use to compare hourly segregation scores, and for models that include percentages of Black and White residents, population sizes, and median household income as control variables.

I use the 2018 Historical Business data from Data Axle, which provide information on the types of organizations and businesses that are present by various spatial units, including census tracts, but does not provide details on operational hours or the demographic distribution of clientele, which would be necessary for further causal analysis on the relationship between businesses and racial segregation. I include all the industries as identified by the North American Industry Classification System (NAICS), such that I can identify associations between industries and racial segregation net of all other industries present. While I preserve 15 of the sectors, I subdivide 8 sectors into 20 different categories to account for categorizations in prior studies, greater internal coherence within categories, and separation between industries with and without indoor facilities. A comprehensive table on how NAICS industries are categorized into sectors can be found in Section F of the Appendix. For example, I divided Arts, Entertainment, and Recreation into three categories: culture, which includes museums, libraries, ballet, and other venues for the arts^33–38^; recreation, which includes sports venues, zoos, and casinos; and other culture, which includes organizations that support the arts, such as agents and managers and promoters without facilities.

I use data on national transit stops provided by the US Department of Transportation. Data from the US Department of Transportation does not include information for Louisville, KY; New Orleans, LA; and San Antonio, TX. Regression results presented in this paper control for public transportation stops, and thereby exclude these cities. Results that include these three cities and exclude transportation stops as a control variable are provided in Section H of the Appendix. These results have insignificant differences between the results that exclude the three cities and include public transportation stops as a control variable.

## Methods

### Clustering data

To reduce the data to locations where people stopped, I cluster the data using density-based spatial clustering of applications with noise (DBSCAN*) for every date and hour in October 2018^22^. I assume a person visited a location if they had at least four data points for that date and hour that are within 0.0001^∘^ from each other, and assume the center of the four or more data points as the location in which a person visited. More details on how I chose the parameters used for DBSCAN* clustering can be found in Section A of the Appendix. After clustering the data for every date and hour of October 2018, I combined the data that are generated for the same day of the week and hour of day for subsequent analyses. Since the data are not generated continuously, I cannot be certain of how long someone visited a location, and consequently, I compute segregation scores based on the clusters that are found within each hour.

### Imputing demographic characteristics

Because the mobile phone data are anonymized, I impute the race of mobile phone owners. To do so, I first identify the most frequently visited census block group for every mobile phone owner and assume that is their home block group. I use home locations by census block groups because they are the smallest geographic unit for which the ACS 2018 provides residential racial demographic information, which is necessary for my imputation strategy. For devices that have multiple home block groups that are tied for most frequent appearances, I randomly select one of the block groups as the home block group.

Other studies assume home locations by using people’s travel patterns in the evening^16,18,22^. Findings that use evening travel patterns to infer home locations can be found in Section D of the Appendix. I defer to using the most frequently visited block groups across all hours as the home block group because this definition includes more unique devices with 15,037,449 devices, as opposed to 9,669,155. I find that even when assuming the most frequently visited census block group as the home block group, the largest proportions of people are found in their home census tracts between 12 and 4 AM. At minimum, 99.1% of the residential block groups among all 49 metropolitan areas have at least one person in the sample. The median proportion of the population of residents of a block group included in the sample is 6.8%.

I use the demographic distribution of the race of residents by census block group to impute race. Specifically, with the 2018 ACS for every block group, I compute the proportions of residents by eight categories of race, including American Indian and Alaska Native, Asian, Black, Hispanic, Native Hawaiian and other Pacific Islander, White, two or more races, and other races. I use these proportions as probabilities of a multinomial distribution to impute race for every mobile phone owner. I repeat this process 100 times and use these imputations to compute 100 Black-White dissimilarity scores for every CBSA for every hour from Monday through Sunday, which generate distributions of estimates. I use these imputations to generate distributions of estimates in subsequent analyses. Imputation is affected by how representative the data are of metropolitan areas, which is discussed at length in Section C of the Appendix.

### Estimating 7-day Black-White Dissimilarity

I use the Black-White Dissimilarity Index, which shows how evenly distributed two groups are within a space as my measure of racial segregation^39^. If all subunits have the same proportions of the two racial groups within a larger geographic area, evenness is maximized. While I compute Black-White Dissimilarity scores for metropolitan areas with census tracts as the subunit, I assign weights to every individual by census block group to account for uneven sampling. I use weights by census block groups to increase precision, as smaller geographic units for weighting will increase precision. Census block groups have population sizes between 600 and 3000 people, while census tracts have between 1200 and 8000 people^40^. For individual *i*, who lives in block group *b*, their weight $w_{i}$ is equal to $\frac{S_{bi}}{s_{bi}}$, where ${S_{bi}}$ is the population size of the block group according to the 2018 ACS, and $s_{bi}$ is the sample size of the block group. Black-White Dissimilarity in its weighted form *D* for hour *v* in metropolitan area *j* with *n* number of census tracts and imputation *t* is defined as:

1 $$\begin{aligned} D_{jtv} = \frac{1}{2} \sum _{a=1}^{n} \Bigg | \frac{\sum _{i=1}^{f_{atv}}w_{aitv}}{F_{jtv}} - \frac{\sum _{i=1}^{g_{atv}}w_{aitv}}{G_{jtv}} \Bigg | \end{aligned}$$

where *i* is an index for individuals; $f_{atv}$, the number of White people within census tract *a* for imputation *t* at hour *v*; $g_{atv}$, the number of Black people in census tract *a* for imputation *t* at hour *v*; $F_{jtv}$, the sum of weights for all White residents of metropolitan area *j* for imputation *t* at hour *v*; and $G_{jtv}$, the sum of the weights for all Black residents of metropolitan area *j* for imputation *t* at hour *v*. To compare how hourly Black-White Dissimilarity scores compare with residential segregation levels, I present ratios between the two with 95% confidence intervals with standard errors of hourly segregation levels throughout the week by metropolitan area. Ratios greater than 1 suggests hourly segregation levels are higher than residential levels, and below 1 suggests they are lower. Results that are unweighted, and that use home location by nighttime travel can be found in the Appendix.

### Explaining patterns in 7-day Black-White Dissimilarity

Through modeling, I investigate which businesses, racial groups, and categories of census tracts contribute to lower levels of Black-White segregation. I compute models separately for every hour of the day because data points from one hour are likely to be correlated with data from the hour prior at minimum. Yet, instead of computing the same model 168 times for every hour of the week, I compute 24 with data that are combined across 7 days for the same hour so that I can include interaction effects between independent variables and an indicator variable for whether the day is part of the weekday or weekend. These interactions are used to gauge which independent variables have different associations between weekdays and weekends. As independent variables, I include counts of businesses in 35 sectors, and 6 variables that account for demographic characteristics, number of public transportation stops, and proportions of residents who are home. I standardize all variables by metropolitan area, to identify average, within-group associations between the outcome and independent variables. I focus on within-group associations to determine the characteristics of census tracts with stronger associations with Black-White segregation, as opposed to differences between metropolitan areas. Standard errors are also clustered by metropolitan area. I run separate models with estimates derived from every imputation of race, and pool coefficients and standard errors with Rubin’s Rule^41^. In sum, models for hour *v*, census tract *a* in metropolitan area *j*, and imputation *t*, are defined as:

2 $$\begin{aligned}{} & {} \begin{aligned} \tilde{y}_{ajtv} =&\beta _{0}+\beta _{1}{day}+\sum _{k=2}^{42} \left[ \beta _{k}\tilde{x}_{akj}+\beta _{k+43}\tilde{x}_{akj}*{day} \right] \end{aligned} \end{aligned}$$

3 $$\begin{aligned}{} & {} \tilde{x}_{akj}=\frac{x_{ak}-\bar{x}_{kj}}{s_{kj}} \end{aligned}$$

where $s_{kj}$ is the standard deviation of variable *k* for metropolitan area *j*, and $\bar{x}_{kj}$ is the average of variable *k* in metropolitan area *j*.

I also compute models with three-way interactions among scaled independent variables, an indicator variable for weekend or weekday, and a factor variable, which categorizes census tracts by those in the top 25th percentile for the proportion of Black residents or White residents by metropolitan area. All remaining census tracts are included in the “Middle 50%” category. With the three-way interaction, I gauge which subsets of census tracts have stronger associations with the outcome of interest. The equation is:

4 $$\begin{aligned} \begin{aligned} \tilde{y}_{ajtv} =&\beta _{0}+\beta _{1}{day}+\sum _{k=2}^{42} \left[ \beta _{k}\tilde{x}_{akj}+\beta _{k+43}\tilde{x}_{akj}*{race} \right. \\&\left. +\beta _{k+86}\tilde{x}_{akj}*{day}+\beta _{k+129}\tilde{x}_{akj}*{day}*{race} \right] \end{aligned} \end{aligned}$$

I use Eqs. (2) and (4) with three different outcome variables.

The first outcome aims to identify the characteristics of tracts that contribute to higher Black-White segregation levels over the weekend. To this end, I use the census tract’s contribution to the metropolitan area’s Black-White Dissimilarity score as the outcome variable, and standardize it by metropolitan areas. The equation is as follows:

5 $$\begin{aligned} \tilde{y}_{ajtv}=\frac{ \left( \Big |\frac{\sum _{i=1}^{f_{atv}}w_{aitv}}{F_{jtv}} -\frac{\sum _{i=1}^{g_{atv}}w_{aitv}}{G_{jtv}} \Big | \right) - \bar{y}_{jtv}}{s_{jtv}} \end{aligned}$$

The second outcome variable is the proportion of Black people within a census tract relative to that of the metropolitan area for a given hour to gauge whether changes in proportions of Black people contribute to variations in Black-White segregation. These proportions are also standardized by metropolitan area and are defined as:

6 $$\begin{aligned} \tilde{y}_{ajtv}=\frac{ \left( \Big |\frac{\sum _{i=1}^{g_{atv}}w_{aitv}}{G_{jtv}} \Big | \right) - \bar{y}_{jtv}}{s_{jtv}} \end{aligned}$$

The third outcome variable is the standardized proportion of White people within a census tract relative to that of the metropolitan area for a given hour to gauge whether changes in proportions of White people contribute to fluctuations in segregation. It is defined as:

7 $$\begin{aligned} \tilde{y}_{ajtv}=\frac{ \left( \Big |\frac{\sum _{i=1}^{f_{atv}}w_{aitv}}{F_{jtv}} \Big | \right) - \bar{y}_{jtv}}{s_{jtv}} \end{aligned}$$

For models with two-way interactions, I compare coefficients for independent variables between weekdays and weekends. If $\beta _{k}$ is the coefficient for variable *k* during weekdays, and $\beta _{kw}$ is the interaction term for variable *k* and an indicator variable *w* for weekends, I compare $\beta _{k}$ with $\beta _{k}+\beta _{kw}$ in all related figures of coefficients.

For models with three-way interactions, I compare coefficients for weekdays and weekends among three categories of census tracts. For variable *k* and census tracts in racial category *d*, I compare $\beta _{k}$ + $\beta _{kd}$ with $\beta _{k}$ + $\beta _{kd}$ + $\beta _{kw}$ + $\beta _{kwd}$, where $\beta _{k}$ is the coefficient for variable *k* during weekdays for the reference group among the three categories of census tracts; $\beta _{kw}$ is the interaction term for variable *k* and weekends for the reference group among the three categories of census tracts; $\beta _{kd}$ is the interaction term for variable *k* on weekdays for racial category *d*; and $\beta _{kdw}$ is the interaction term for variable *k* on weekends for racial category *d*.

Select variables with noteworthy associations with Black-White segregation and large differences in their associations between weekdays and weekends are presented. Individual coefficient plots for all independent variables are included in the Appendix.

## Results

### Black-White segregation is higher on weekends

**Figure 1:**
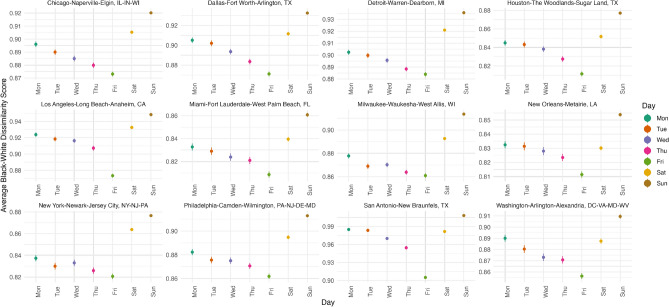
Weighted average Black-White dissimilarity ratios by day.

Figure 1 shows the 95% confidence intervals with standard errors of ratios between residential segregation and weighted, daily segregation for 12 metropolitan areas. A graph with all 49 metropolitan areas can be found in Section D of the Appendix. All metropolitan areas have segregation levels on Sunday that are significantly larger than any other day of the week. Excluding New Orleans, LA; San Antonio, TX; and Washington, DC, segregation levels are higher on Saturdays and Sundays for all other metropolitan areas. Also notable is that segregation levels decrease incrementally between Monday through Friday, and on Friday, all metropolitan areas are most integrated.

**Figure 2:**
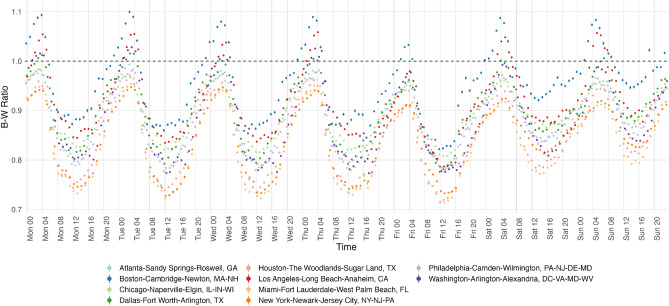
Hourly, weighted Black-White dissimilarity.

Figure 2 shows 95% confidence intervals with standard errors of the ratios between residential segregation and 100 imputed Black-White Dissimilarity scores for the 10 most populous metropolitan areas, although standard errors are noticeably smaller relative to the means. Hourly fluctuations in Black-White Dissimilarity corroborate the findings of Athey et al.^17^ for Monday through Friday, in which levels are lowest around 12PM, and are highest around 3AM. Figure 2 shows that higher daily average segregation levels over weekends are driven by higher levels of segregation over daytime hours on weekends compared to that of weekdays. I also find that on Sundays, segregation levels peak between 4 AM and 8 AM, as opposed to 12 AM and 4 AM, as is the case on weekdays. This difference signals differences in late evening travel patterns over weekends.

**Figure 3:**
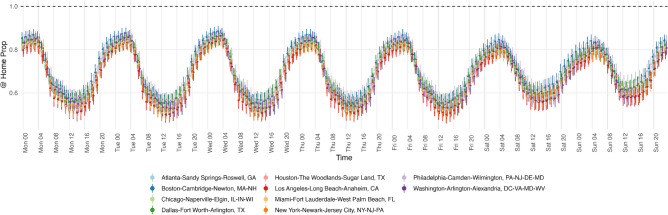
Weighted average proportions of travel to home census tracts.

Figure 3 shows the 95% confidence intervals with standard errors of the proportion of people who traveled to their home census tracts for every hour of the week. Patterns are comparable with that of Black-White Dissimilarity, with higher proportions of people being found in their home census tracts over the weekend during daytime hours. Black-White Dissimilarity Scores reach their peak when the largest proportions of visits are to people’s home census tracts. Section E of the Appendix has additional analysis of how the demographic characteristics of residents and time are associated with proportions of visits to home census tracts by hour. I find that proportions of Black residents have a larger association than proportions of White residents, which shows that residents of predominantly Black neighborhoods stay within their neighborhoods at higher rates than residents of predominantly White neighborhoods. I also find interaction effects between hour of day and an indicator variable for the weekend are almost always significantly positive, which shows that people stay within their home neighborhoods at higher rates on weekends.

### Geographic heterogeneity is observed in the counts and contributions to Black-White segregation by neighborhoods with different industries

**Figure 4:**
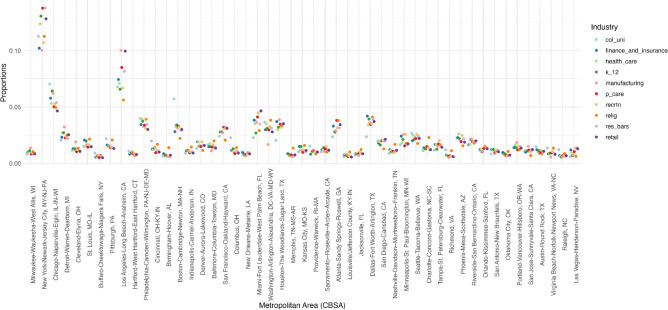
Proportion of industries by metropolitan area.

**Figure 5:**
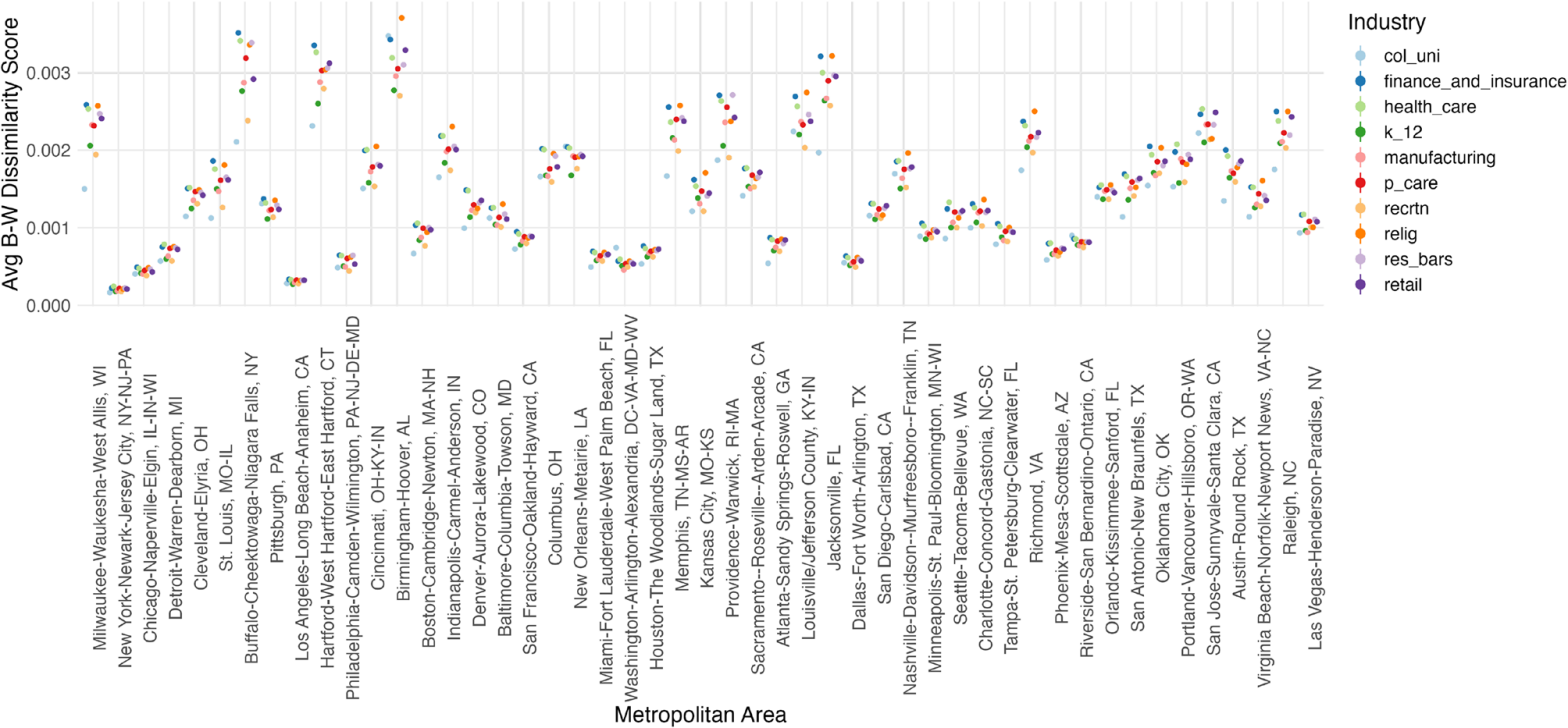
Average B–W dissimilarity scores of neighborhoods by place.

Figures 4 and 5 show geographic heterogeneity of the distribution of businesses and the racial segregation of the neighborhoods, in which they are embedded. Metropolitan areas are listed according to their rank by Black-White Dissimilarity scores based on the ACS 2018, such that Milwaukee, WI is the most segregated, and Las Vegas, NV is the least.

Specifically, Fig. 4 shows the proportion of businesses in a metropolitan area for a sample of industries. Several cities have larger proportions of businesses than the expected percentage of 2%. These cities include New York, NY; Chicago, IL; Los Angeles, CA; Philadelphia, PA; Miami, FL; Boston, MA; Houston, TX; Washington, DC; and Dallas, TX. Given the concentration of businesses in the largest cities, subsequent modeling strategies will standardize business counts by metropolitan area to isolate the average contribution of businesses within metropolitan areas, as opposed to capturing disparities between metropolitan areas.

Figure 5 shows the average contribution to the respective metropolitan area’s Black-White segregation among census tracts with different types of industries between the hours of 8 AM and 8 PM from Monday through Friday. Surprisingly, greater racial segregation levels for the broader metropolitan area does not mean greater average contributions to racial segregation among census tracts with different industries. While religious organizations and financial businesses have the highest average racial segregation by neighborhood for most metropolitan areas, colleges and universities have the smallest averages. Whether these trends hold net of demographic characteristics by residents of neighborhoods and the presence of other businesses is examined in the next section.

### A majority of industries have significantly different associations with Black-White segregation between weekdays and weekends

**Figure 6:**
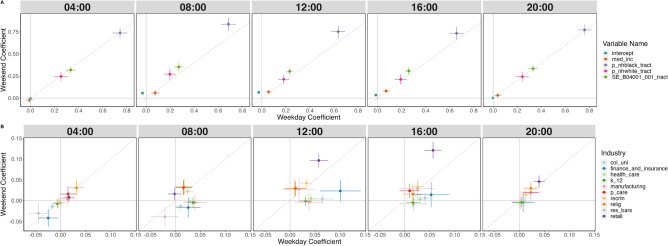
Coefficients for models with two-way interactions and B–W dissimilarity decompositions as the dependent variable.

**Figure 7:**
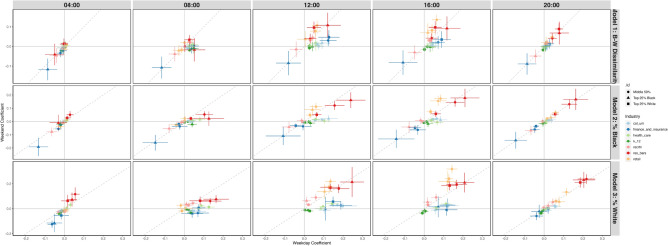
Coefficients for models with three-way interactions.

Figure 6 plots coefficients that are derived from models that account for temporal heterogeneity by using two-way interaction terms to gauge how industries and the census tracts they are in contribute to the metropolitan area’s overall Black-White segregation differently between weekdays and weekends. These models account for geographic heterogeneity by scaling variables by metropolitan area.

Figure 6a shows coefficients for all demographic characteristics included in the models, which have the strongest associations with Black-White segregation compared to categories of businesses regardless of the day of the week. Specifically, among all 41 independent variables, proportions of Black residents have the largest associations with contributions to Black-White Dissimilarity scores, followed by the population sizes of census tracts, and proportions of white residents, and median income. All demographic variables have significantly positive weekend effects. The intercept, which represents the average contribution to the metropolitan area’s Black-White Dissimilarity score for census tracts with average levels of all the independent variables, is higher on weekends than weekdays.

Figure 6b shows coefficients for industries for weekdays and weekends net of residential demographics and other businesses and organizations. Figure 6b showcases two groups of businesses: those that have larger associations with racial segregation over the weekend compared to associations over weekdays, and those that have larger associations over weekdays.

Figure 7 shows coefficients from three-way interaction models among industries, weekends, and categories of census tracts by percentage of Black or White residents. Each row has different outcome variables, but the independent variables and interaction terms used in the models are identical. In general between 8 AM and 4 PM, while there are industries that have significantly negative associations with proportions of Black people present, none of the industries have significantly negative associations with proportions of White people.

#### Ten industries, including retail, recreation, and religious organizations have significantly greater associations with Black-White segregation over weekends

Although associations are not as large as that of residential characteristics, there are notable relationships between businesses and Black-White segregation that contribute to elevated segregation levels over weekends. Retail businesses, which include clothing, electronics, and jewelry stores have the largest association with Black-White segregation across all 35 categories of organizations, and these numbers become significantly larger on weekends from the afternoon on wards. Other types of retail, including food and home retail, do not share this trend, and their coefficient plots can be found in Section H of the Appendix. This finding is surprising given prior work that finds retail trade has the largest levels of racial integration among its employees, which suggests that the higher segregation levels are driven in part by customers. Figure 7 shows that the associations between retail businesses and proportions of Black people are significantly larger in census tracts with larger proportions of non-White residents.

Recreation, including zoos and botanical gardens and casinos; restaurants and bars; religious organizations; and personal care businesses, which includes beauty salons and laundry services, have significantly larger associations with Black-White segregation compared to that of weekdays. As shown in Fig. 7, while recreation has a significantly positive association with proportions of White people across all census tract categories, recreation has a significantly negative association with proportions of Black people in census tracts with the largest proportions of Black residents. Restaurants and bars also have a significantly positive association with proportions of White people across all census tract categories without preference, yet with proportions of Black people as the outcome, restaurants and bars have significantly higher associations in census tracts that have larger proportions of non-White residents.

While not shown in Fig. 6b, accommodation, which includes hotels; construction; information, which includes radio stations, and television broadcasting; professional, scientific, and technical services; other services, which include appliance repair and funeral homes, have several hours when segregation levels on weekends are significantly higher than that of weekdays.

#### Twelve industries, including finance and insurance, colleges and universities, and health care, have significantly greater associations with Black-White segregation on weekdays

Finance and insurance businesses have the largest association with Black-White segregation among all categories of institutions on weekdays between 9 AM and 3 PM. As Fig. 7 shows, finance and insurance businesses are associated with average and below average proportions of Black people in all categories of census tracts. Surprisingly, the coefficient is most negative for institutions in census tracts with the largest proportions of Black residents. Prior work reports that minority neighborhoods have less access to banks than White neighborhoods^42^. In addition to lower supply, the fact that proportions of Black people are lowest even in census tracts in the 25th percentile for proportions of Black residents suggests less demand for these services.

Academic institutions are associated with higher contributions to Black-White segregation on weekdays, and significantly smaller associations on weekends. After accounting for demographic variables and the presence of other businesses, colleges and universities have larger associations with Black-White segregation than K-12 schools. This is a surprising finding, given that uncontrolled results from Fig. 2 show that neighborhoods with colleges and universities have the lowest average contributions to several metropolitan areas’ overall Black-White Dissimilarity scores. Other industries in the educational sector, which includes driving and cosmetology schools, have significantly negative associations with racial segregation, and do not have significant differences in associations between weekdays and weekends for most hours.

Health care and manufacturing sectors also have notable differences in their associations with segregation during weekdays and weekends. Health care has positive associations with Black-White segregation that lasts from 7 AM to 5 PM on weekdays, and all three categories of census tracts have an increase in their associations. Meanwhile, manufacturing has positive associations between 8 AM and 12 PM on weekdays, and census tracts in the top 25th percentile for proportions of White residents are the only category with significantly positive associations. Both sectors have insignificant associations with racial segregation on weekends.

Several other categories have significantly higher associations with Black-White segregation on weekdays compared to that of weekends, but the difference is not as stark as the aforementioned sectors. They include public administration; social assistance; culture, which includes bookstores, libraries, and museums; home retail; real estate; residential care; and wholesale trade. Their coefficient plots can be found in Section H of the Appendix.

#### Thirteen industries, including accommodations and agriculture, do not have significant weekend interaction effects

Other accommodation, which include RV campgrounds; agriculture, forestry, fishing, and hunting; management companies; and transportation and warehousing have significantly positive associations with racial segregation that are consistent between weekdays and weekends.

Administrative, support, waste management, and remediation services; other cultural businesses, which include promoters without facilities; other schools, which include driving and cosmetology schools have consistently and significantly negative associations with racial segregation during daytime hours throughout the week.

Several industries have insignificant associations with Black-White segregation during daytime hours. They are: civil and social organizations; food retail; mining; other retail, which includes car dealers, and used merchandise stores; other restaurants and bars, which includes catering and mobile food services; and utilities.

## Discussion

This study shows the pervasiveness of racial segregation, an unrelenting feature of American society that has been typically studied in the context of residential neighborhoods and schools, and demonstrates its temporality throughout the week. Specifically, this study shows three main findings. First, this study shows that the distinction between weekdays and weekends is important in terms of racial segregation, with racial segregation levels being higher over weekends than on weekdays for all metropolitan areas. Hourly trends slightly shift, such that segregation levels are highest between 12 AM and 4 AM on weekdays, and between 4 AM and 8 AM on weekends, and are lowest at later hours in the afternoon on weekends compared to weekdays. Hourly segregation levels during the daytime are also much higher on weekends compared to weekdays. These findings allude to variances in racial segregation throughout the day and week, and raise questions on how periodic fluctuations in racial segregation affects racial dynamics.

Second, elevated segregation levels over the weekend can be attributed to larger proportions of people being home during the day, and relatedly, larger associations between Black-White segregation and residential demographic characteristics over weekends. Proportions of Black residents have the largest associations net of the organizational landscape of census tracts. Moreover, proportions of Black people are found to increase more notably in non-White neighborhoods, while proportions of White people increase at similar rates across all categories of census tracts. Hence, Black people seem to travel to places with larger proportions of non-White residents for the majority of organizational resources.

Third, most categories of businesses have associations with racial segregation that differ between weekdays and weekends, and a large proportion has a significantly positive association with racial segregation during weekday, daytime hours. While much attention has been given to racial segregation among K-12 schools, I find that financial institutions, retail, and colleges and universities have greater associations with Black-White segregation than that of K-12 schools on weekdays. Additionally, for many categories of businesses, the racial distribution of the people who are present during business hours differs from that of after business hours and over weekends, which suggests that the demographic makeup of daytime visitors, which include employees and customers, differ from that of residents.

While this study cannot definitively make causal claims due to data limitations, this study shows that race remains salient in the local environments when businesses are most active for a broader range of sectors than previously understood. The dissimilar associations among organizations and Black-White segregation during daytime hours between weekdays and weekends show a strong relationship exists. Several mechanisms could explain these trends. A large body of literature finds persistent racial discrimination of Black people as candidates for jobs, which means that racially segregated employment opportunities could partially explain these associations^43–45^. Many studies also describe poor treatment of racial minorities in retail and consumer markets in neighborhoods with predominantly White residents, which could deter racial minorities from spending time in spaces with larger proportions of White people^46,47^. Racial minorities could also prefer to visit businesses and organizations with larger proportions of co-ethnic customers and members for social solidarity and availability of ethnic products^48–50^. Alternatively, non-business related visitors, especially for businesses with publicly accessible spaces, could also have a role in promoting racial integration^51^.

Prior studies identify the benefits of ethnic diversity on innovation, influence, and profit^52–56^. Future studies can not only distinguish the roles of employees, customers, and passersby in the fluctuating levels of racial segregation, but can also explore the effects of diversity of the broader spaces in which companies are embedded on company outcomes. Findings from this paper suggest that racial segregation is not a feature isolated to residential neighborhoods and schools, and that businesses should consider ways to reduce racial segregation within their local surroundings to collectively diminish the reaches of racial segregation and its role in perpetuating racial inequality.

### Supplementary Information

Supplementary Information.

## Data Availability

Code materials can be found at https://github.com/jjc09/rhythms_week. Mobile phone location data from Fysical cannot be shared under a data-sharing agreement. 2018 Historical Business Data cannot be shared publicly, but can be accessed through Wharton Data Research Services hosted by the University of Pennsylvania. The 2018 American Community Survey and data on national transit stops from the US Department of Transportation are publicly available.
