# Supplementary material for: 7-day patterns in Black-White segregation in 49 metropolitan areas
Source: Sci Rep. 2024 Mar 20;14:6740. doi: 10.1038/s41598-024-56257-1 (PMC10954647; doi:10.1038/s41598-024-56257-1)

Online Appendix:
7-Day Patterns in Black-White Segregation in 49 Metropolitan Areas

Joanna Chae, *Columbia University in the City of New York*

Appendix A: Clustering Location Data

I use DBSCAN* for R to cluster data points. Clusters had at least 4 points that were at minimum 0.0001 degrees apart from each other. I used the KNN algorithm to identify epsilon. The KNN distance plot is below. Data that were not included in a cluster were excluded from further analysis. Mobile devices without any clusters were also removed from further analysis.

Figure A1:KNN Distance Plot

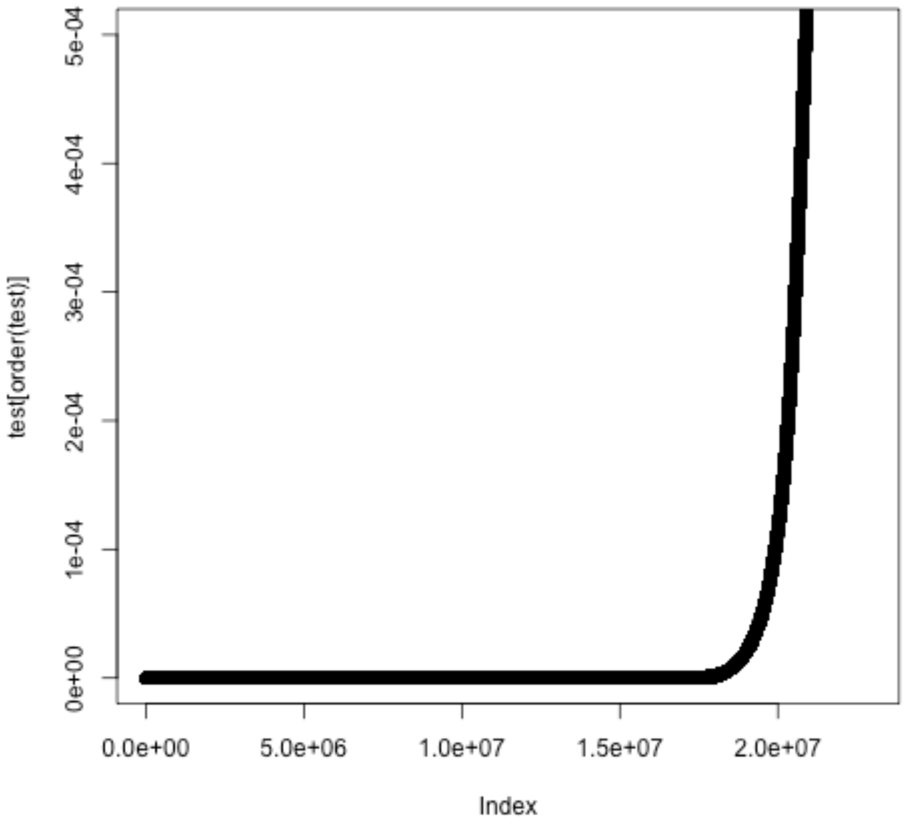

I found the center of every cluster and identified the corresponding block group. I found the block group for the cluster that had the most data points for every hour, and identified the most frequently occurring block group as the home block group.

Appendix B: Sample Statistics

Figure B1: Sample Sizes by Metropolitan Areas

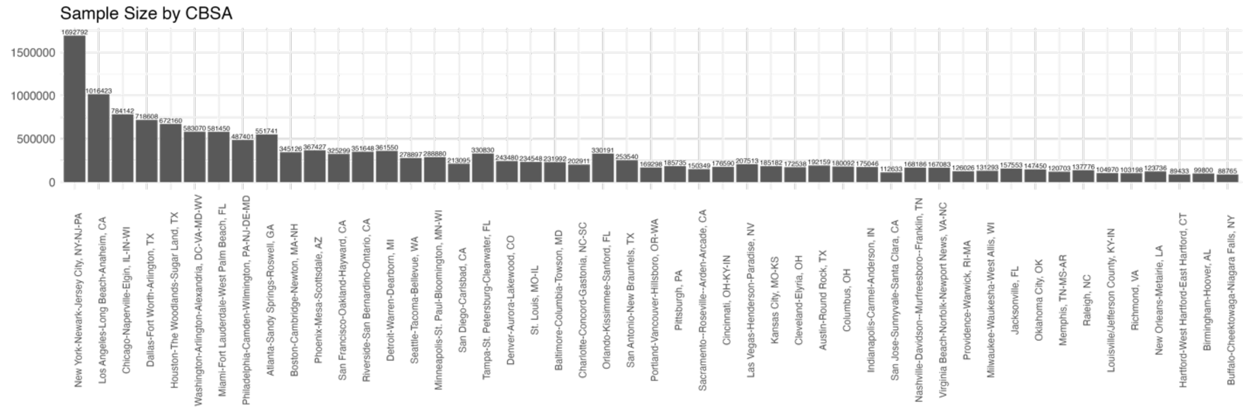

Figure B1 shows the counts of unique devices with an identifiable home location in every metropolitan area. Metropolitan areas are listed in order of rank by population size, such that New York, NY has the largest population and Buffalo, NY has the smallest.

Figure B2. Proportions of Sample Size vs Resident Population

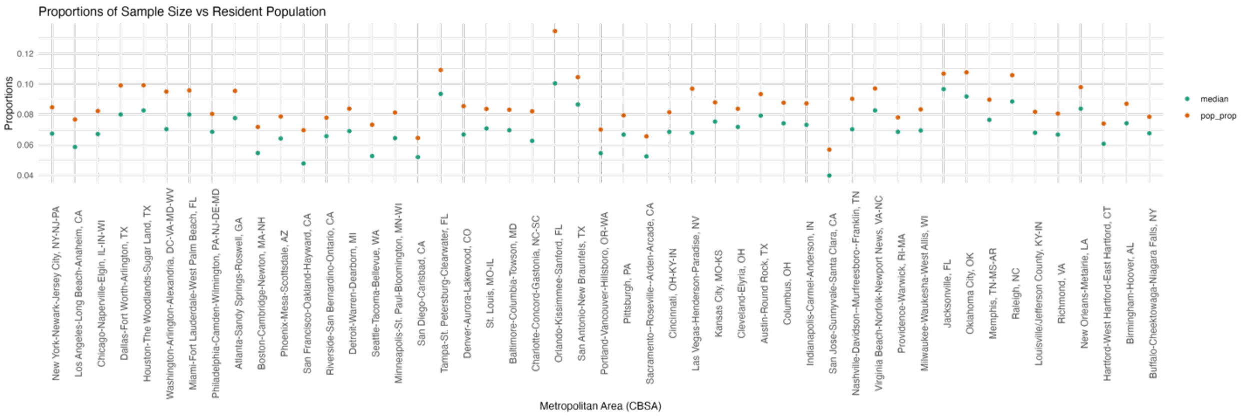

Pop_prop in Figure B2 shows the proportion of the sample in metropolitan areas with the total population of metropolitan areas. San Jose, CA has the smallest proportion at approximately 6-percent, and Orlando, FL has the largest proportion at approximately 15-percent. Median shows the median proportion of the sample of block groups with the population of block groups. Similar to pop_prop, San Jose, CA has the lowest median at a little above 4-percent, and Orlando, FL has the largest median at 11-percent.

Figure B3. Numbers of Unique Devices and Combinations of Unique Devices and Census Tracts by Hour

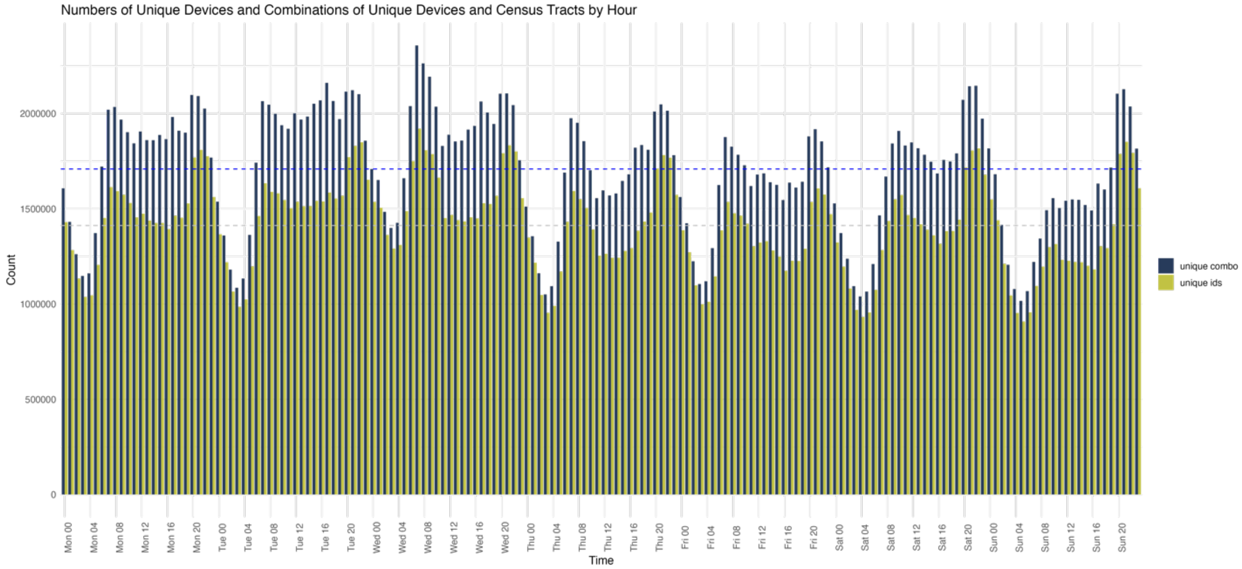

Figure B3 shows the unique numbers of active devices in green, and the unique number of devices and census tracts in which those mobile devices are found. The gray dashed line is the average number of active devices for all hours and days, and the blue dashed line is the average number of combinations in devices and census tracts. Fewer than average numbers of devices and combinations of devices and unique destinations are observed on Sundays, which suggests that elevated levels of racial segregation on Sunday are the result of less mobility. Yet, elevated levels on Saturday cannot be explained by less movement, as there are above average numbers of unique devices and unique combinations of devices and destinations observed throughout most of the day.

Appendix C: Representativeness of the Data
Representativeness of the data is important for imputation, as how well the entire metropolitan area is represented in the data will affect how accurate the estimates are. One assumption in my imputation strategy is that people of all racial categories have equal probabilities of owning smartphones and consenting to their data being shared. The [Pew Research Center] estimates around 77% to 81% of the US population owned smartphones near the end of 2018, and in 2021, differences by racial categories were relatively small.

The Pearson's correlation coefficient between the population of census block groups that had at least one resident in the ACS 2018 and the sample size of residents found in the mobile phone location data is 0.397, which shows moderate correlation between the mobile phone location data and the ACS 2018.

Figure C1: Summary Statistics by Demographic Variables

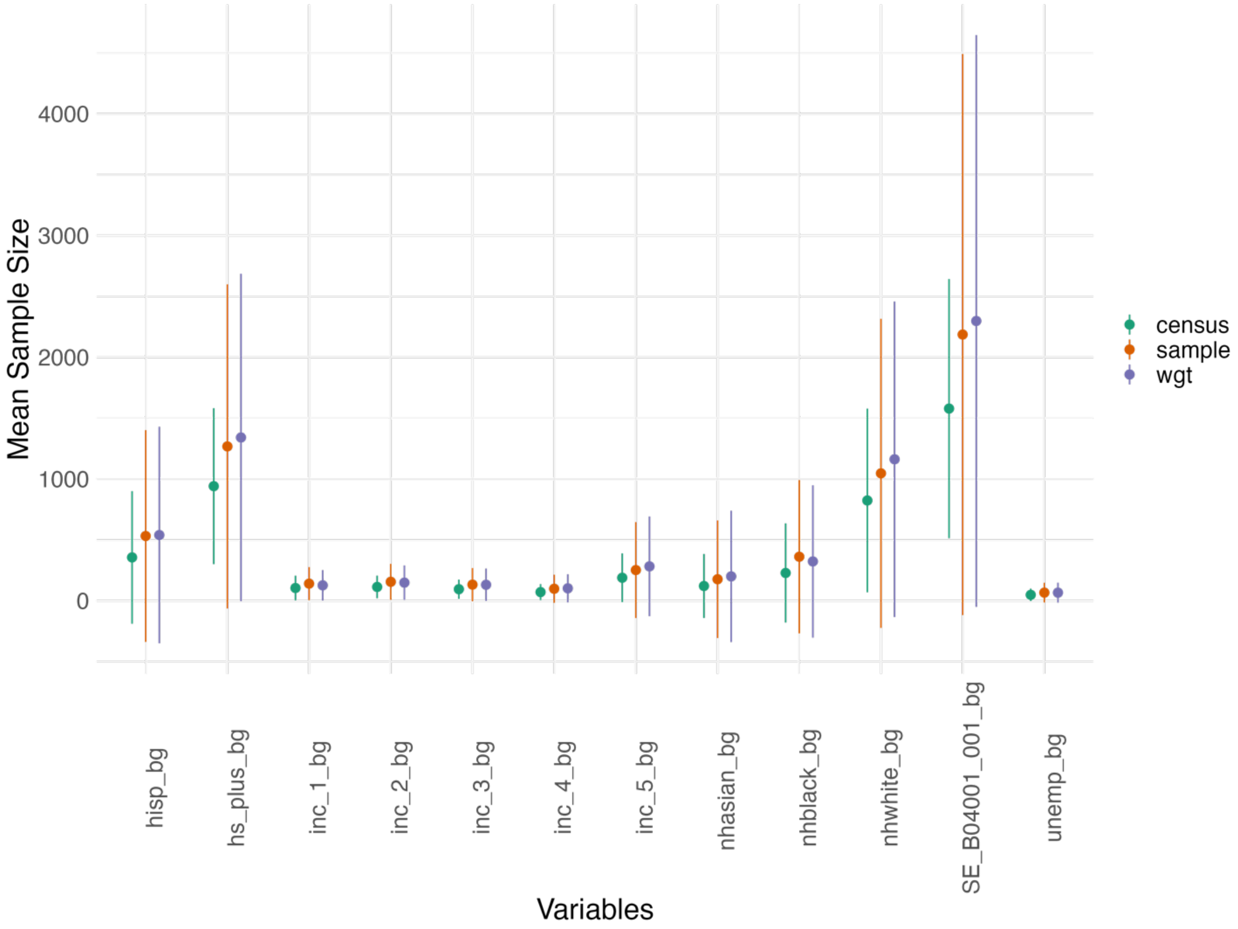

I compute average counts of various demographic variables from the ACS 2018. I compare these averages with the averages from the mobile phone data, and present the averages in Figure C1, as well as 1 standard deviation above and below the average. While the mobile phone data have larger average counts in all the demographic variables, there is strong overlap among the distribution of counts.

Table C2: Regression Models of Number of Devices by Census Block Group

	# Devices/ Z-Score	# Devices/Population Z-Score
inc_1_bg	0.145***	0.020***
	(0.004)	(0.004)
inc_2_bg	0.090***	0.017***
	(0.004)	(0.005)
inc_3_bg	0.101***	0.008*
	(0.004)	(0.005)
inc_4_bg	0.074***	0.004
	(0.004)	(0.005)
inc_5_bg	0.202***	-0.014
	(0.008)	(0.009)
unemp_bg	0.021***	0.007*
	(0.003)	(0.004)
hs_plus_bg	-0.227***	0.008
	(0.013)	(0.014)
nhwhite_bg	0.212***	0.195***
	(0.008)	(0.009)
nhblack_bg	0.179***	0.082***
	(0.005)	(0.005)
nhasian_bg	0.081***	0.052***
	(0.004)	(0.004)
hisp_bg	0.214***	0.121***
	(0.005)	(0.005)
ln_pop	-0.155***	-0.350***
	(0.005)	(0.006)
Observations	112,941	112,941
R2	0.212	0.034
Adjusted R2	0.212	0.034
F Statistic (df = 61; 112879)	498.301***	65.633***
CBSA Fixed Effects	Yes	Yes
Note: *p<0.1; **p<0.05; ***p<0.001		

I regressed counts and proportions of sample sizes to the census block group population with demographic characteristics with fixed effects for metropolitan areas. Excluding metropolitan areas, all variables have been scaled. I use these models to gauge which subgroups of the population are present at greater levels in the mobile phone data. These models show that the mobile phone data have larger proportions of people from lower income categories and education levels. White people are present at larger levels than non-White people.

Figure C3. Residential vs Location Data Segregation Scores

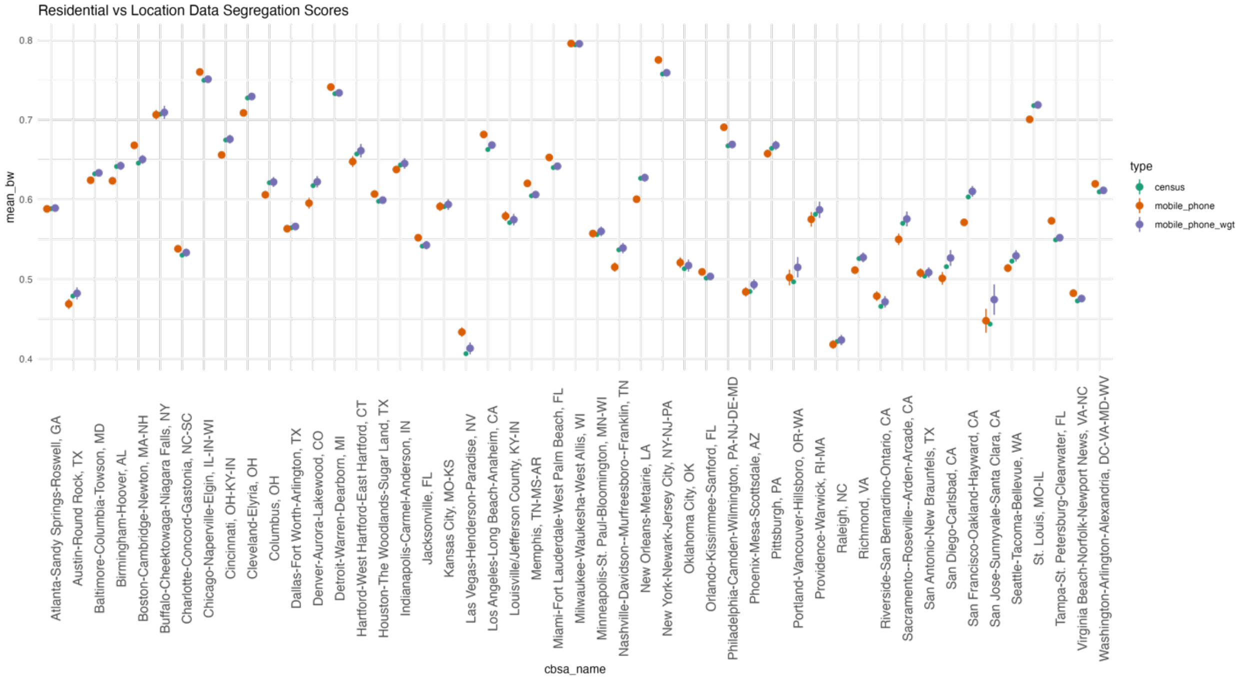

I first computed weighted and unweighted Black-White Dissimilarity scores for every imputation of race. I took the means of these scores and compared them with residential segregation in Figure C3. Figure C3 shows that if all mobile phone owners were found in their home census tracts, weighted experienced segregation would be fairly similar to residential segregation. This figure shows that all variations seen throughout the day and week are the result of movement. The overlap also shows that the mobile phone data has sufficient coverage of the metropolitan area, such that the sorting of members of different racial categories by census tracts are very similar.

Appendix D: Variations in Experienced Segregation

Figure D1. Daily Averages of Weighted Black-White Dissimilarity Scores

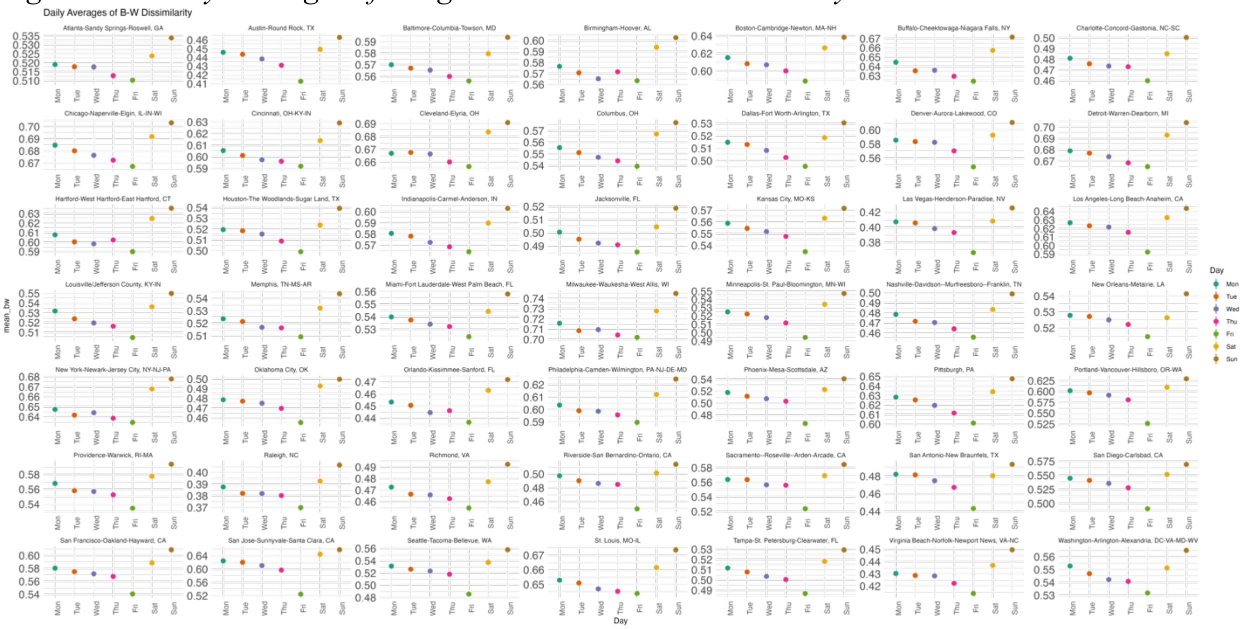

Figure D2. Daily Averages of Unweighted Black-White Dissimilarity Scores

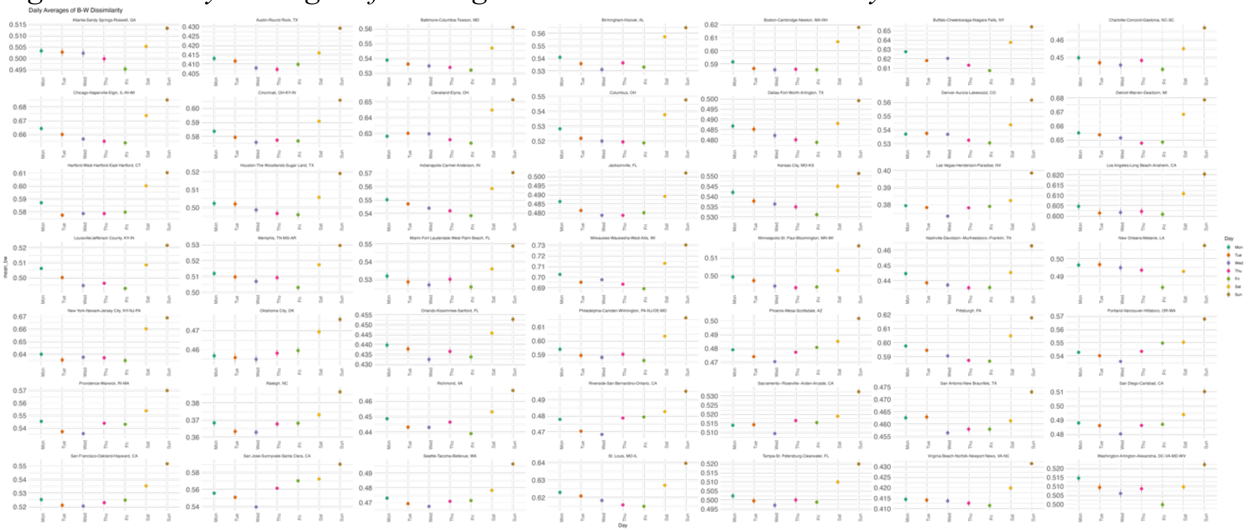

Figure D3. Unweighted Black-White Dissimilarity Residents Only

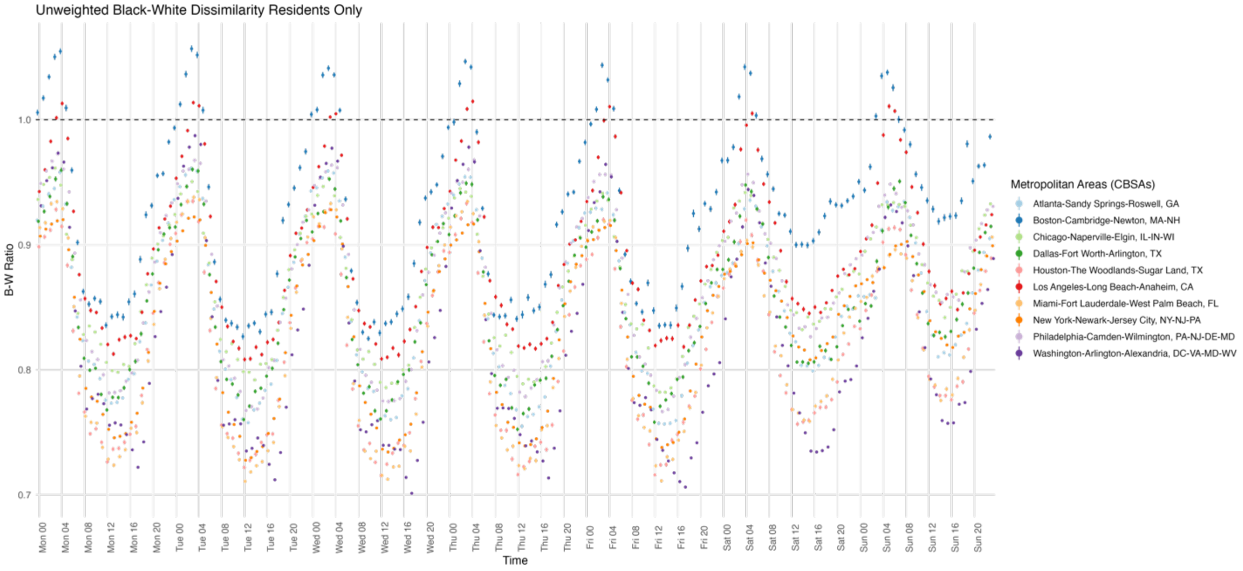

Figure D4. Weighted Black-White Dissimilarity with Nighttime (8PM-12AM) Travel Patterns

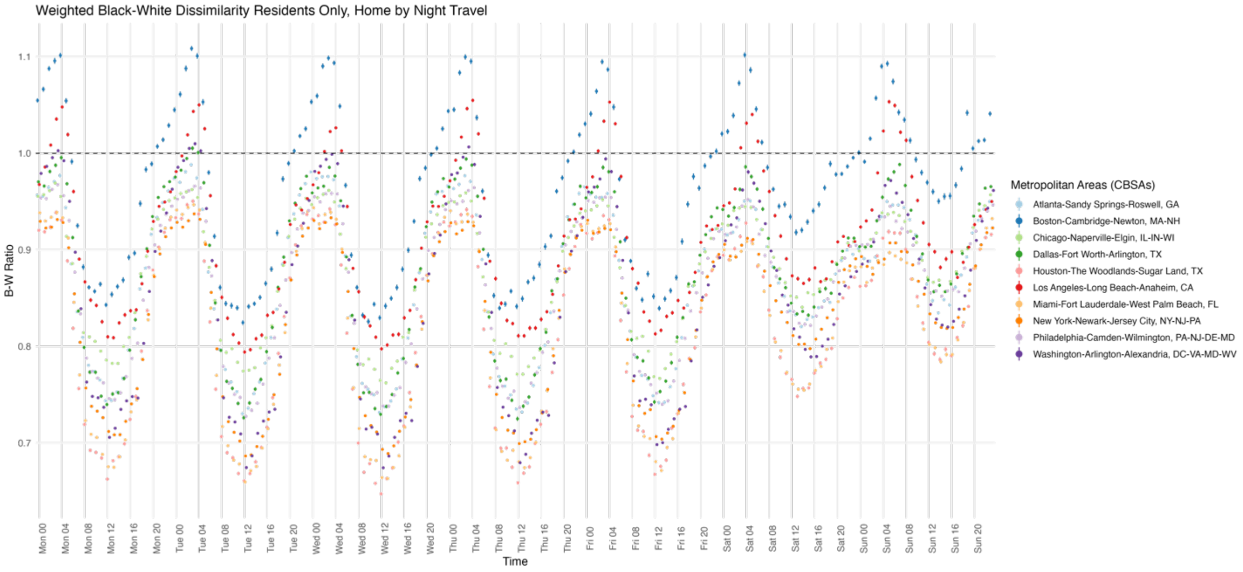

Appendix E: % At Home

	% At Home/Z-Score by CBSA
p_nhblack_tract	0.032*** (0)
p_nhwhite_tract	0.016*** (0.001)
SE_B04001_001_tract	0.286*** (0)
transp_count	-0.132*** (0)
count	-0.341*** (0)
med_inc	-0.006*** (0.001)
hr00	0.427*** (0.006)
weekend	-0.303*** (0.004)
hr01	0.472*** (0.006)
hr01:weekend	0.003 (0.006)
hr02	0.495*** (0.006)
hr02:weekend	0.023*** (0.006)
hr03	0.524*** (0.006)
hr03:weekend	0.07*** (0.006)
hr04	0.488*** (0.006)
hr04:weekend	0.174 (0.006)
hr05	0.328*** (0.006)
hr05:weekend	0.344***(0.006)
hr06	0.054*** (0.006)
hr06:weekend	0.551*** (0.006)
hr07	-0.322*** (0.006)
hr07:weekend	0.837*** (0.006)
hr08	-0.557*** (0.006)
hr08:weekend	0.926*** (0.006)
hr09	-0.636*** (0.006)
hr09:weekend	0.804*** (0.006)
hr10	-0.722*** (0.006)
hr10:weekend	0.702*** (0.006)
hr11	-0.849*** (0.006)
hr11:weekend	0.649*** (0.006)
hr12	-0.941*** (0.006)
hr12:weekend	0.637*** (0.006)
hr13	-0.939*** (0.006)
hr13:weekend	0.606*** (0.006)
hr14	-0.957*** (0.006)
hr14:weekend	0.632*** (0.006)
hr15	-0.931*** (0.006)
hr15:weekend	0.622*** (0.006)
hr16	-0.849*** (0.006)
hr16:weekend	0.570*** (0.006)
hr17	-0.763*** (0.006)
hr17:weekend	0.540*** (0.006)
hr18	-0.610*** (0.006)
hr18:weekend	0.448*** (0.006)
hr19	-0.390*** (0.006)
hr19:weekend	0.358*** (0.006)
hr20	-0.123*** (0.006)
hr20:weekend	0.293*** (0.006)

hr21	0.078*** (0.006)
hr21:weekend	0.252*** (0.006)
hr22	0.218*** (0.006)
hr22:weekend	0.216*** (0.006)
hr23	0.308*** (0.006)
hr23:weekend	0.198*** (0.006)
Observations	3775248
R2	0.491
Adjusted R2	0.4909
F-Statistic (df=99; 3775149)	3.678e+04***
CBSA Fixed Effects	Yes
Note: *p<0.1; **p<0.05; ***p<0.001	

The above OLS model has the proportion of people at home as the dependent variable. All demographic variables have been scaled by CBSA. "Count" is the total count of businesses and organizations that have also been scaled by CBSA. The model was computed with CBSA fixed effects.

I find that census tracts with more than the proportions of Black residents have higher than average proportions of people at home. Census tracts with more White residents also have higher than average proportions of people at home, but this association is not as large as that of the proportion of Black residents. The population size of the census tract has the largest association with the proportion of people who are home. Number of public transportation stops, median income, and counts of businesses have negative associations with proportions of people being at home.

The model also includes variables for hours of the day, which were all interacted with an indicator variable for weekend. I find that during weekdays, hours have a negative association with proportions of people who are home between 7AM and 8AM. With the exception of 12AM and 1AM on weekends, interaction coefficients are significantly positive, meaning that for most hours of the day, larger proportions of people are found in their home census tracts. On weekends, there is a negative association with proportions of people at home from 10AM to 7PM, which is a shorter range of time compared to weekdays.

These spans of hours confirms that the strategy of inferring the most frequently visited census tract as the home census tract does not mean I find the largest proportions of people are at home at non-evening hours.

Appendix F: Industry Grouping

Category	Top 10 Unique NAICS8 Descriptions	Count	Proportion	Rank
Health Care	all other misc ambulatory health care services, freestanding ambulatory surgical & emergency ctrs, offices of all other misc health practitioners, offices of chiropractors, offices of dentists, offices of optometrists, offices of physicians (exc mental health specs), offices-mental health practitioners (exc physcns), offices-physical, occptnl/speech thrpsts/audlgsts, pharmacies & drug stores	1579978	20.00%	1
Professional, Scientific, and Technical Services	all other professional, scientific/technical svcs, architectural services, engineering services, marketing consulting services, offices of certified public accountants, offices of lawyers, other management consulting services, process, physical distr/logistics consulting svcs, tax preparation services, veterinary services	771795	9.80%	2
Finance and Insurance	all other nondepository credit intermediation, commercial banking, consumer lending, credit unions, direct property & casualty insurance carriers, financial trnsctns procng, reserve/clrnghs actvts, insurance agencies & brokerages, investment advice, miscellaneous intermediation, real estate credit	524164	6.60%	3
Construction	all other specialty trade contractors, commercial & institutional building construction, electrical contr & other wiring installation contr, new single-family hsng constr (exc for-sale bldrs), painting & wall covering contractors, plumbing htg & air-conditioning contractors, poured concrete foundation & structure contractors, residential remodelers, roofing contractors, site preparation contractors	482620	6.10%	4
Restaurants and Bars	drinking places alcoholic beverages, full-service restaurants, limited-service restaurants, cafeterias, grill buffets & buffets, snack & nonalcoholic beverage bars	402041	5.10%	5
Real Estate, Rental, and Leasing	general rental centers, lessors of miniwarehouses & self-storage units, lessors of residential buildings & dwellings, lessors-nonresidential bldgs (exc miniwarehouses), offices of real estate agents & brokers, offices of real estate appraisers, other activities related to real estate, passenger car rental, residential property managers, truck, utility trailer & rv rental & leasing	336589	4.30%	6
Retail	electronic stores, family clothing stores, florists, gift, novelty & souvenir stores, jewelry stores, other clothing stores, other gasoline stations, shoe stores, sporting goods stores, women's clothing stores	289202	3.70%	7
Other Services	all other personal services, appliance repair & maintenance, automotive body, paint & interior repair/maint, car washes, coml/ind mach/equip (exc auto/elctrnc) rpr/maint, computer & office machine repair & maintenance, funeral homes & funeral services, general automotive repair, other personal & household goods repair & maint, other personal care services	279018	3.50%	8
Administrative and Support, and Waste Management and	all other support services, carpet & upholstery cleaning services, employment placement agencies, exterminating & pest control services, janitorial services, landscaping	269910	3.40%	9

Remediation Services	services, locksmiths, office administrative services, security guards & patrol services, travel agencies			
Personal Care	all other health & personal care stores, barber shops, beauty salons, coin-operated laundries & drycleaners, cosmetics, beauty supplies & perfume stores, diet & weight reducing centers, drycleaning & laundry svcs (except coin-operated), nail salons, pet care (except veterinary) services	242378	3.10%	10
Wholesale Trade	brick, stone/related constr material mrchnt whlsrs, electrical apparatus/wiring supls/rel equip whlsrs, industrial machinery & equipment merchant whlsrs, medical, dental/hospital equip/supls mrchnt whlsrs, motor vehicle supplies & new parts merchant whlsrs, other grocery & related products merchant whlsrs, other miscellaneous durable goods merchant whlsrs, other petroleum merchant wholesale, recyclable material merchant wholesalers, wholesale trade agents & brokers	217676	2.80%	11
Manufacturing	all other misc fabricated metal product mfg, all other miscellaneous manufacturing, all other plastics product manufacturing, commercial printing (except screen & books), commercial screen printing, dental laboratories, machine shops, sign manufacturing, surgical & medical instrument manufacturing, wood kitchen cabinet & countertop manufacturing	207568	2.60%	12
Social Assistance	child & youth services, child day care services, community food services, emergency & other relief services, other community housing services, other individual & family services, services for the elderly/persons with disabilities, temporary shelters, vocational rehabilitation services	190189	2.40%	13
Other Retail	all other misc store retailers (exc tobacco strs), automotive parts & accessories stores, boat dealers, fuel dealers, motorcycle, atv & all other motor vehicle dealers, new car dealers, other direct selling establishments, tire dealers, used car dealers, used merchandise stores	164866	2.10%	14
Religious Organizations	religious organizations	149475	1.90%	15
Food Retail	all other specialty food stores, beer, wine & liquor stores, confectionery & nut stores, convenience stores, fish & seafood markets, food (health) supplement stores, fruit & vegetable markets, meat markets, retail bakeries, supermarkets/other grocery (exc convenience) strs	135191	1.70%	16
Public Administration	courts, executive offices, fire protection, legislative bodies, national security, other general government support, other human resource programs administration, police protection, public finance activities, regulation & administration-transportation prgrms	130727	1.70%	17
Transportation and Warehousing	all other transit & ground passenger trnsprtn, freight transportation arrangement, general freight trucking, local, general warehousing & storage, limousine service, motor vehicle towing, postal service, specialized freight (exc used gds) trckng lng-dist, support activities for rail transportation, taxi service	126976	1.60%	18
Home Retail	all other home furnishings stores, floor covering stores, furniture stores, hardware stores, home centers,	125822	1.60%	19

	household appliance stores, nursery, garden center & farm supply stores, other building material dealers, paint & wallpaper stores, window treatment stores			
Civil and Social Organizations	business associations, civil & social organizations, environment, conservation & wildlife organizations, human rights organizations, labor unions & similar labor organizations, other grantmaking & giving services, other similar organizations, other social advocacy organizations, political organizations, professional organizations	118567	1.50%	20
Information	all other information services, all other publishers, all other telecommunications, data processing, hosting & related services, motion picture & video production, newspaper publishers, periodical publishers, radio stations, software publishers, sound recording studios	102623	1.30%	21
K-12 Schools	elementary & secondary schools	80970	1.00%	22
Recreation	all other amusement & recreation industries, amusement & theme parks, bowling centers, casinos (except casino hotels), fitness & recreational sports centers, golf courses & country clubs, marinas, nature parks & other similar institutions, racetracks, sports teams & clubs	79125	1.00%	23
Other School	all other miscellaneous schools & instruction, automobile driving schools, cosmetology & barber schools, educational support services, exam preparation & tutoring, fine art schools, language schools, other technical & trade schools, professional & management devmnt training, sports & recreation instruction	77292	1.00%	24
Culture	art dealers, book stores, libraries & archives, motion picture theaters (except drive-ins), museums, musical groups & artists, musical instrument & supplies stores, other performing arts companies, promoters with facilities, theater companies & dinner theaters	73600	0.90%	25
Accommodation	hotels (except casino hotels) & motels, bed-&-breakfast inns	31849	0.40%	26
Residential Care	assisted living facilities for the elderly, continuing care retirement communities, nursing care facilities (skilled nursing fclts), other residential care facilities, residential mental hlth/substance abuse facilities	30854	0.40%	27
Agriculture, Forestry, Fishing, and Hunting	all other animal production, all other miscellaneous crop farming, dairy cattle & milk production, grape vineyards, hunting & trapping, nursery & tree production, other noncitrus fruit farming, soil preparation planting & cultivating, support activities for animal production, support activities for forestry	18256	0.20%	28
Other Restaurants and Bars	food service contractors, caterers, mobile food services	15656	0.20%	29
Other Culture	promoters without facilities, agents & managers for public figures, independent artists, writers & performers	14797	0.20%	30
Management of Companies and Enterprises	offices of bank holding companies, offices of other holding companies	9125	0.10%	31
Other Accommodations	all other traveler accommodation, rv (recreational vehicle) parks & campgrounds, recreational & vacation camps (except campgrounds), rooming & boarding houses-dormitories-worker camps	8231	0.10%	32

Utilities	electric power distribution, natural gas distribution, other electric power generation, sewage treatment facilities, steam & air-conditioning supply, water supply & irrigation systems	7432	0.10%	33
Colleges and Universities	junior colleges, colleges, universities & professional schools	7429	0.10%	34
Mining, Quarrying, and Oil, and Gas Extraction	all other nonmetallic mineral mining, bituminous coal & lignite surface mining, clay ceramic & refractory minerals mining, construction sand & gravel mining, crushed & broken limestone mining & quarrying, dimension stone mining & quarrying, drilling oil & gas wells, industrial sand mining, other crushed & broken stone mining & quarrying, support activities for oil & gas operations	4245	0.10%	35

Appendix G: Regression Statistics

Sample size 1 has sample sizes for the regression models I computed that include the number of public transportation stops per census tract. Given that there are a total of 37,397 census tracts in the 46 metropolitan areas that have readily available data on the number of public transportation stops, and every census tract can have a maximum of 7 measurements of contributions to Black-White Dissimilarity for every day of the week, the maximum possible sample size is 269,955. This means that for every hour around 98.8% of the maximum possible combinations is included in the regression models. Regression models do not have all 100% of the maximum possible combinations because some census tracts may be missing measurements for and are excluded from the data.

Sample size 2 has sample sizes of the regression models that are included in the Appendix that include Louisville, KY; New Orleans, LA; and San Antonio, TX, but exclude the number of public transportation stops as an independent variable. There are a total of 38,565 census tracts in all 49 metropolitan areas, which makes the maximum possible sample size to be 269,955. Here also, around 98.8% of the maximum possible sample size is included in the regression models.

Also presented are the average adjusted R-squared values across the 100 regression models computed for every hour and the standard deviations. Adjusted R-Squared 1 is for the regression models that exclude Louisville, KY; New Orleans, LA; and San Antonio, TX, and Adjusted R-Squared 2 is for the regression models that include Louisville, KY; New Orleans, LA; and San Antonio, TX.

Hour	Sample Size 1	Adjusted R-Squared 1	Sample Size 2	Adjusted R-Squared 2
0	258579	0.478 (0.002)	266612	0.474 (0.002)
1	258542	0.471 (0.002)	266576	0.468 (0.002)
2	258513	0.46 (0.002)	266548	0.456 (0.002)
3	258483	0.451 (0.003)	266518	0.448 (0.003)
4	258489	0.45 (0.002)	266525	0.447 (0.002)
5	258536	0.449 (0.002)	266572	0.447 (0.002)
6	258586	0.452 (0.003)	266622	0.449 (0.003)
7	258618	0.45 (0.003)	266653	0.446 (0.003)
8	258632	0.44 (0.003)	266668	0.436 (0.003)
9	258626	0.433 (0.003)	266662	0.43 (0.003)
10	258629	0.425 (0.002)	266665	0.421 (0.002)
11	258619	0.409 (0.002)	266655	0.405 (0.002)
12	258626	0.399 (0.003)	266662	0.395 (0.002)
13	258628	0.396 (0.002)	266664	0.392 (0.002)
14	258625	0.393 (0.002)	266661	0.389 (0.002)
15	258617	0.394 (0.003)	266653	0.391 (0.002)
16	258622	0.398 (0.003)	266656	0.394 (0.003)
17	258625	0.416 (0.002)	266658	0.412 (0.002)
18	258626	0.424 (0.002)	266661	0.419 (0.002)

19	258608	0.437 (0.002)	266644	0.434 (0.002)
20	258623	0.468 (0.002)	266659	0.465 (0.002)
21	258625	0.48 (0.002)	266661	0.477 (0.002)
22	258610	0.486 (0.002)	266646	0.483 (0.002)
23	258610	0.483 (0.002)	266644	0.479 (0.002)

Linear Regression Assumptions

The following graphs test four assumptions for linear regression. The Residuals vs Fitted plot tests linearity. No pattern is observed, meaning linearity is upheld. The Scale-Location plot tests homogeneity of variance. There is a trend in this plot which shows that heteroskedasticity is likely. The Q-Q Residuals plot tests whether residuals are normally distributed. While the majority of points follow a linear trend, a quarter of the points do not follow this trend. The Residuals vs Leverage plot shows outliers and leverage points, which do exist, but the plot does not have a visible trend.

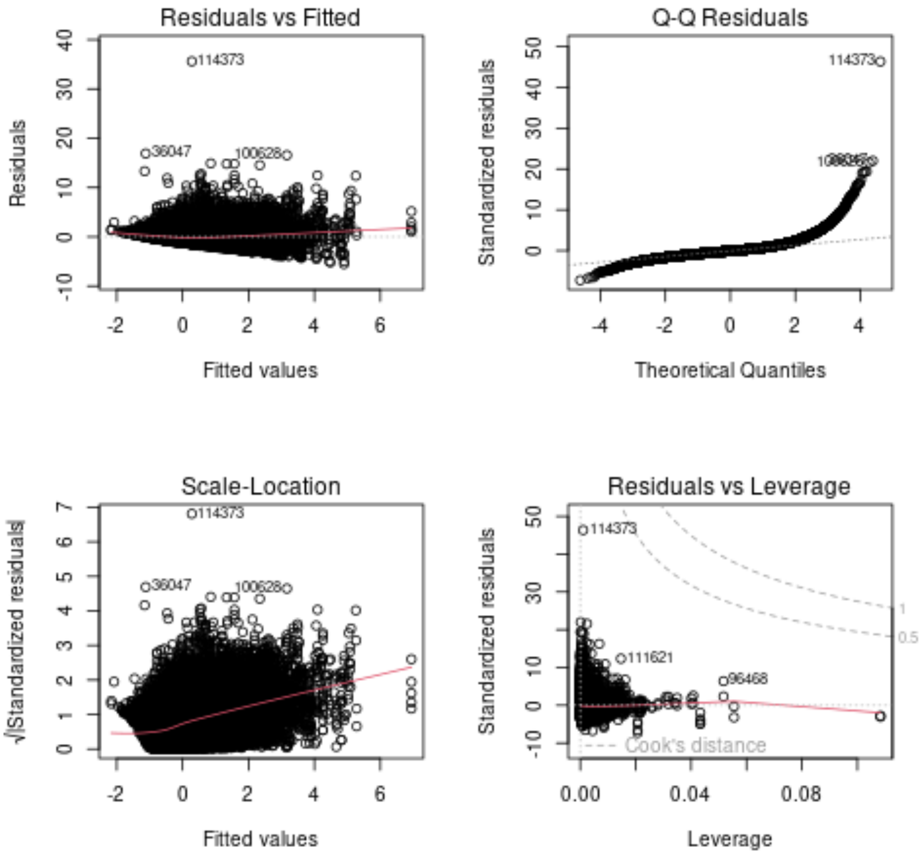

Metropolitan Area	Census Tract Counts
New York-Newark-Jersey City, NY-NJ-PA	4700
Los Angeles-Long Beach-Anaheim, CA	2929
Chicago-Naperville-Elgin, IL-IN-WI	2215
Dallas-Fort Worth-Arlington, TX	1324
Houston-The Woodlands-Sugar Land, TX	1072
Washington-Arlington-Alexandria, DC-VA-MD-WV	1359
Miami-Fort Lauderdale-West Palm Beach, FL	1219
Philadelphia-Camden-Wilmington, PA-NJ-DE-MD	1477
Atlanta-Sandy Springs-Roswell, GA	951
Boston-Cambridge-Newton, MA-NH	1007
Phoenix-Mesa-Scottsdale, AZ	991
San Francisco-Oakland-Hayward, CA	980
Riverside-San Bernardino-Ontario, CA	822
Detroit-Warren-Dearborn, MI	1301
Seattle-Tacoma-Bellevue, WA	721
Minneapolis-St. Paul-Bloomington, MN-WI	789
San Diego-Carlsbad, CA	628
Tampa-St. Petersburg-Clearwater, FL	746
Denver-Aurora-Lakewood, CO	621
St. Louis, MO-IL	615
Baltimore-Columbia-Towson, MD	683
Charlotte-Concord-Gastonia, NC-SC	539
Orlando-Kissimmee-Sanford, FL	390
San Antonio-New Braunfels, TX	457
Portland-Vancouver-Hillsboro, OR-WA	491
Pittsburgh, PA	711
Sacramento--Roseville--Arden-Arcade, CA	486
Cincinnati, OH-KY-IN	498
San Juan-Carolina-Caguas, PR	598
Las Vegas-Henderson-Paradise, NV	487
Kansas City, MO-KS	530
Cleveland-Elyria, OH	638
Austin-Round Rock, TX	350
Columbus, OH	433
Indianapolis-Carmel-Anderson, IN	397
San Jose-Sunnyvale-Santa Clara, CA	383
Nashville-Davidson--Murfreesboro--Franklin, TN	380
Virginia Beach-Norfolk-Newport News, VA-NC	422

Providence-Warwick, RI-MA	370
Milwaukee-Waukesha-West Allis, WI	431
Jacksonville, FL	262
Oklahoma City, OK	363
Memphis, TN-MS-AR	314
Raleigh, NC	224
Louisville/Jefferson County, KY-IN	306
Richmond, VA	295
New Orleans-Metairie, LA	405
Hartford-West Hartford-East Hartford, CT	290
Birmingham-Hoover, AL	264
Buffalo-Cheektowaga-Niagara Falls, NY	299

Appendix H: Coefficient Plots

Each variable has three coefficient plots. The first one is from the model with two-way interactions, and the second plot is from the models with three-way interactions. The last plot is from models without public transportation stops as an independent variable, which includes three additional metropolitan areas: Louisville, KY; New Orleans, LA; and San Antonio, TX.

Accommodation

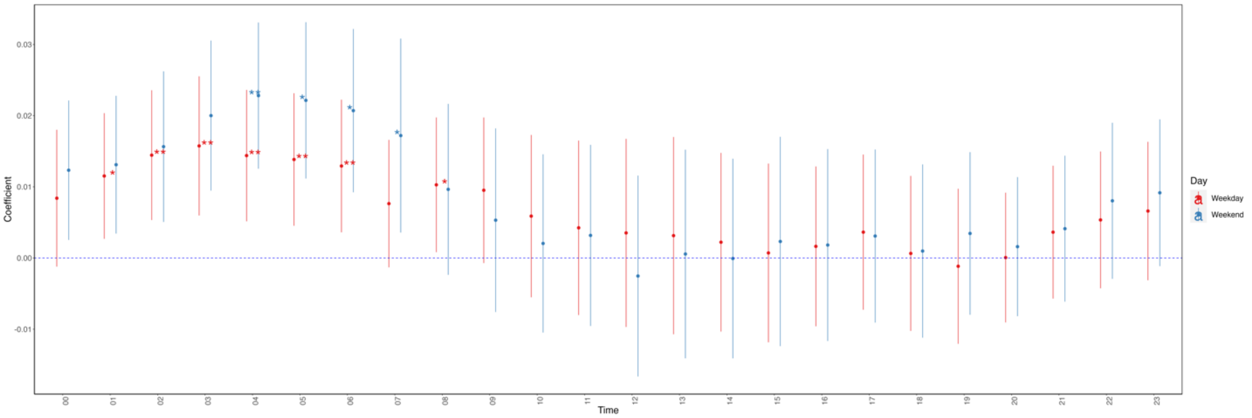

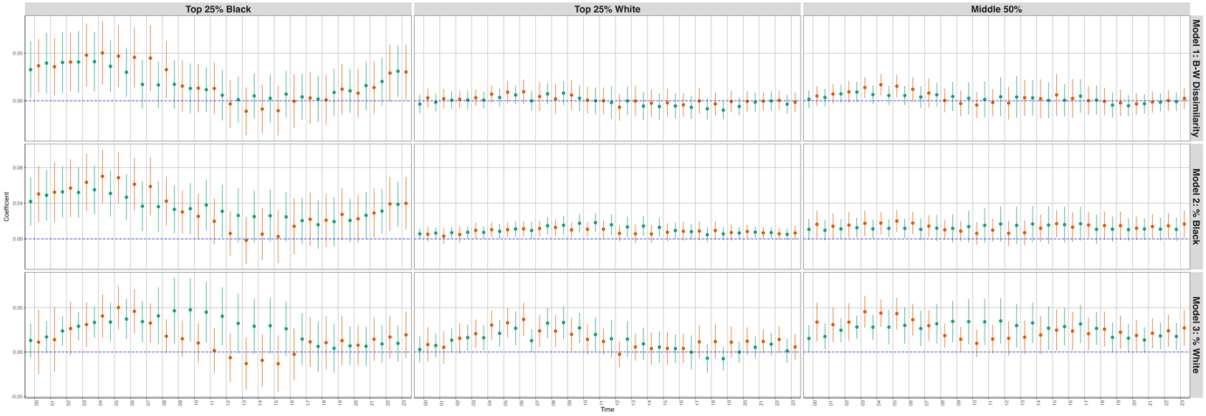

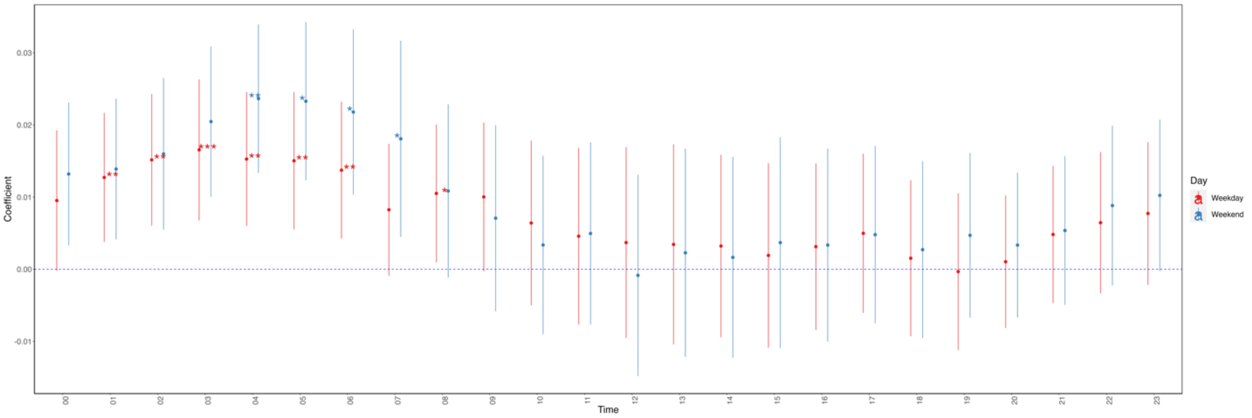

Administrative and Support and Waste Management and Remediation Services

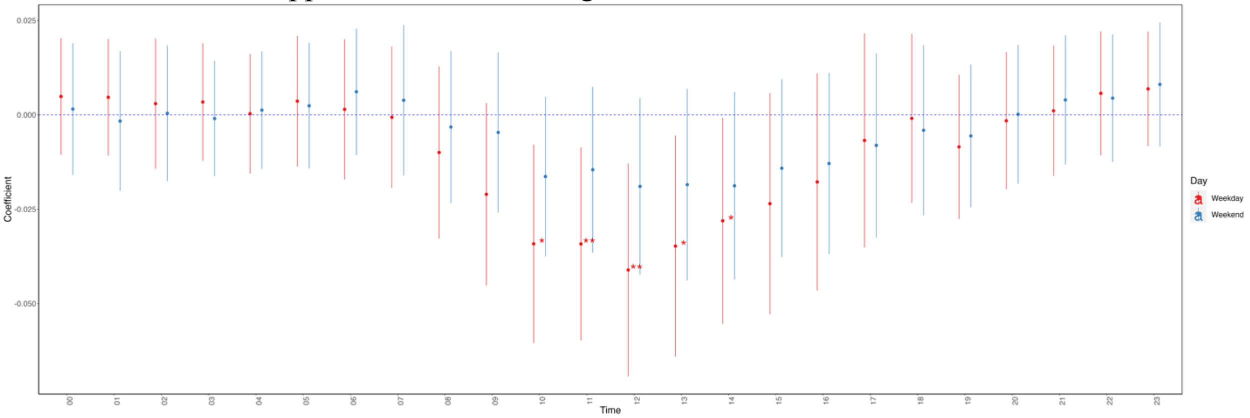

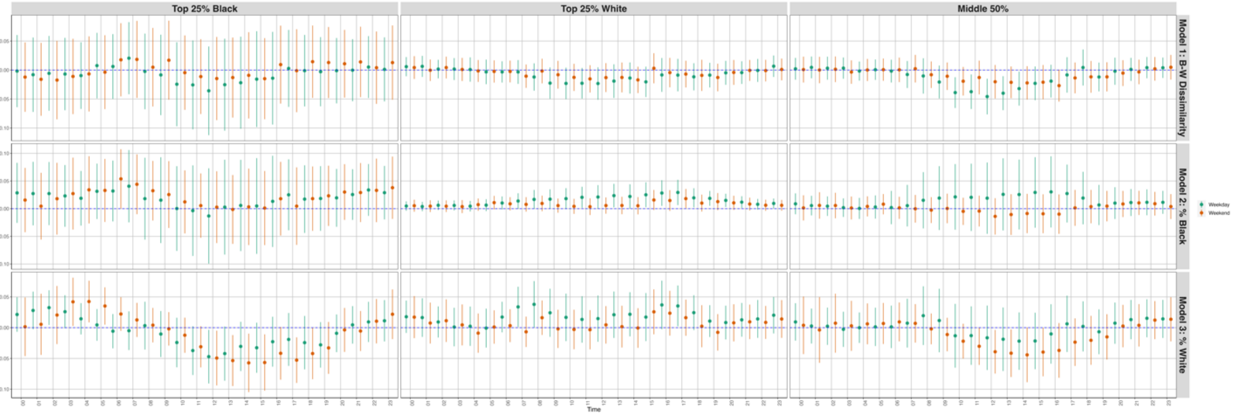

Models without public transportation stops as an independent variable, with three additional metropolitan areas (Louisville, KY; New Orleans, LA; and San Antonio, TX)

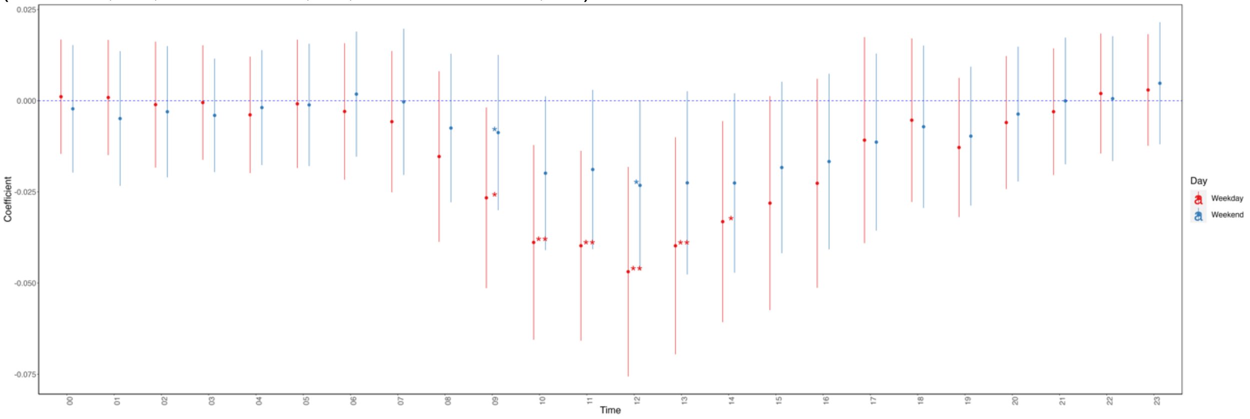

Agriculture, Forestry, Fishing, and Hunting

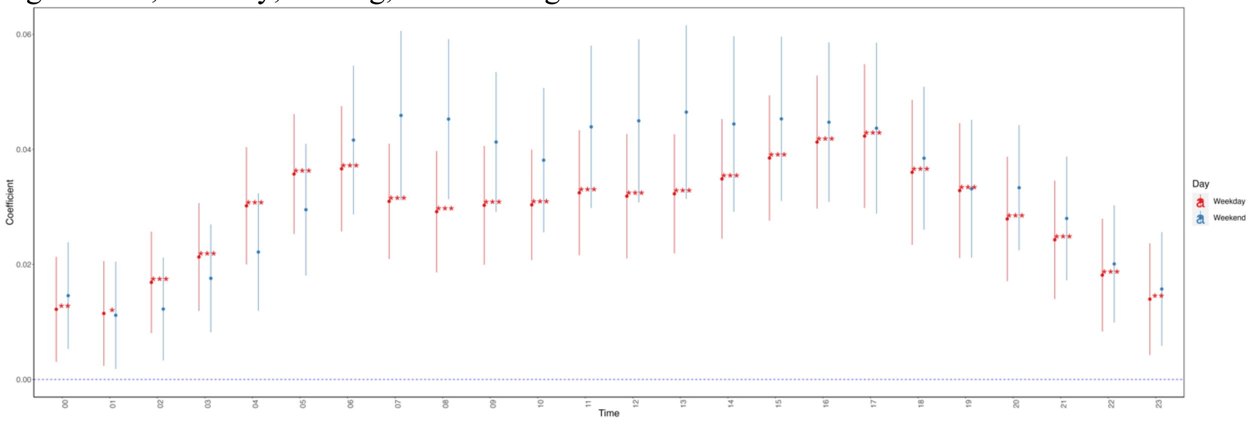

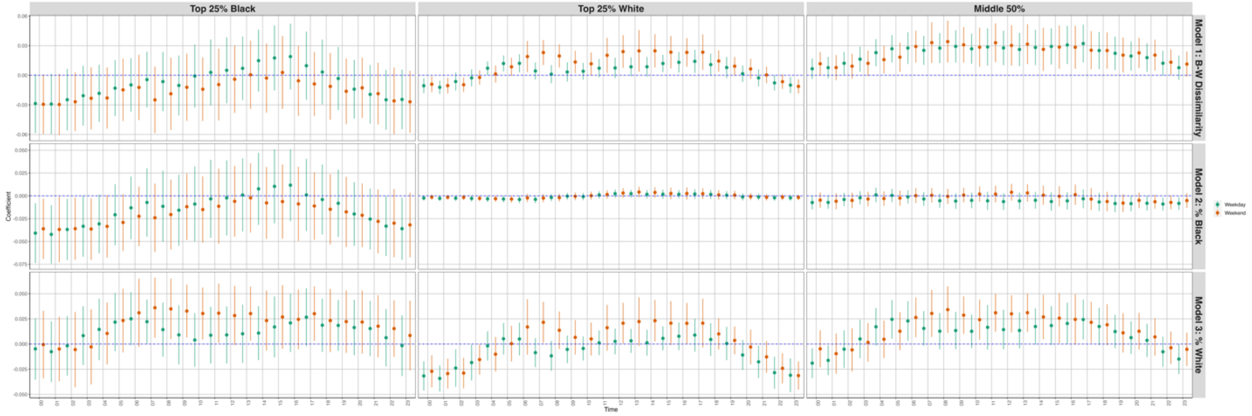

Models without public transportation stops as an independent variable, with three additional metropolitan areas (Louisville, KY; New Orleans, LA; and San Antonio, TX)

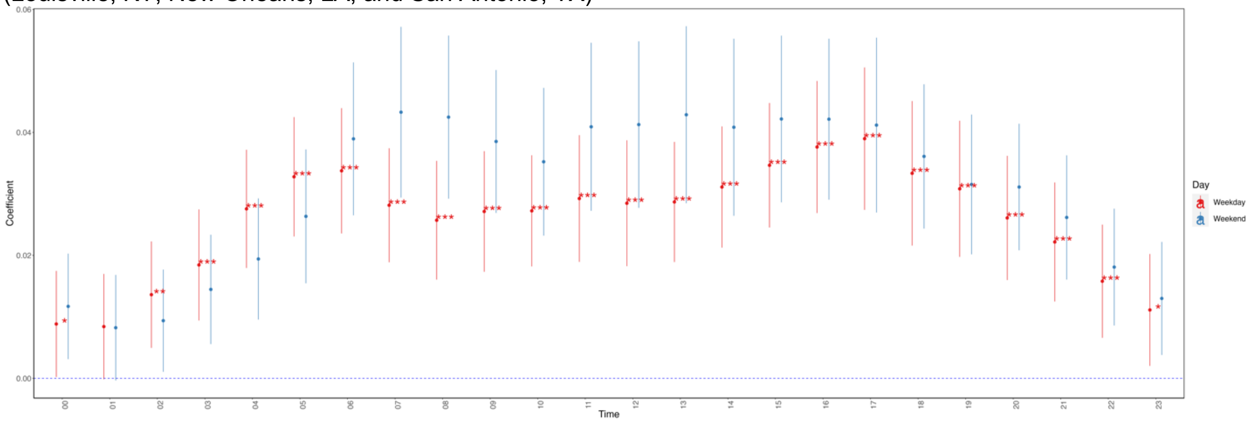

Civil and Social Organizations

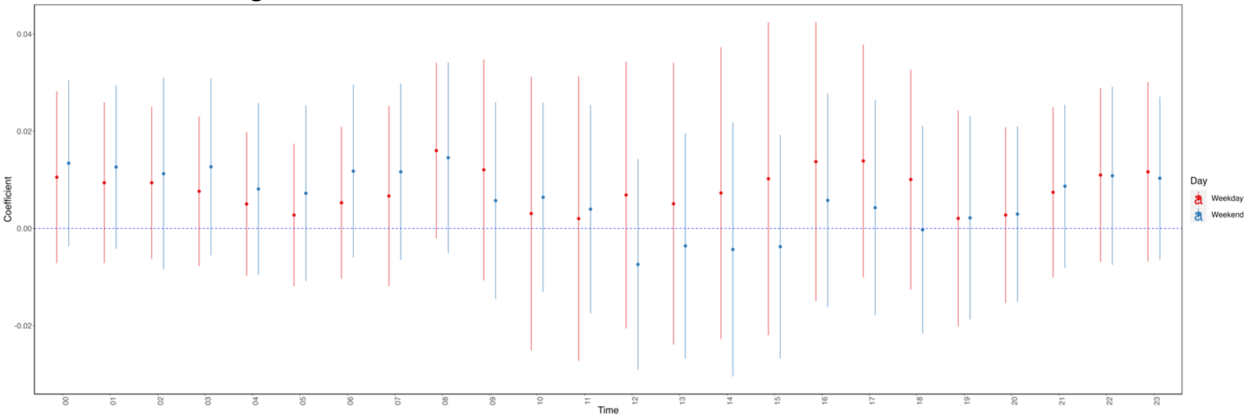

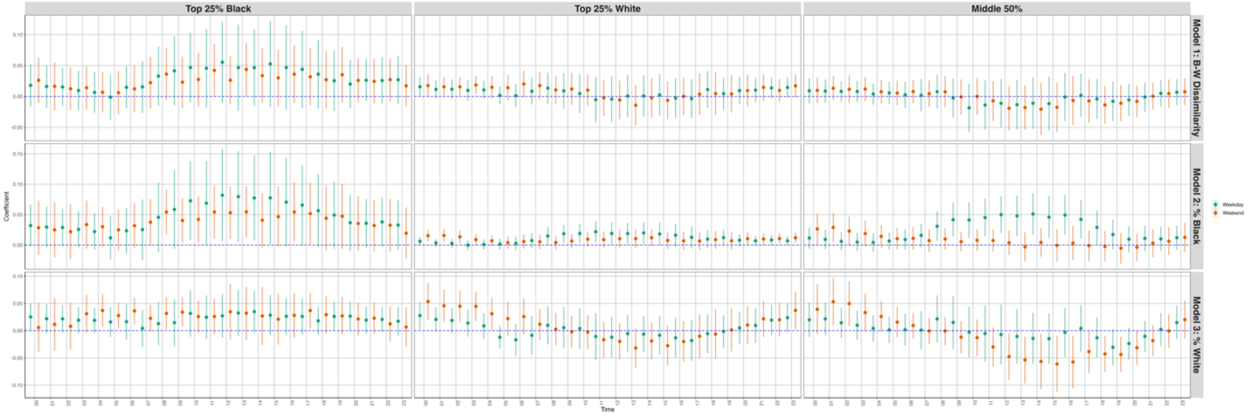

Models without public transportation stops as an independent variable, with three additional metropolitan areas (Louisville, KY; New Orleans, LA; and San Antonio, TX)

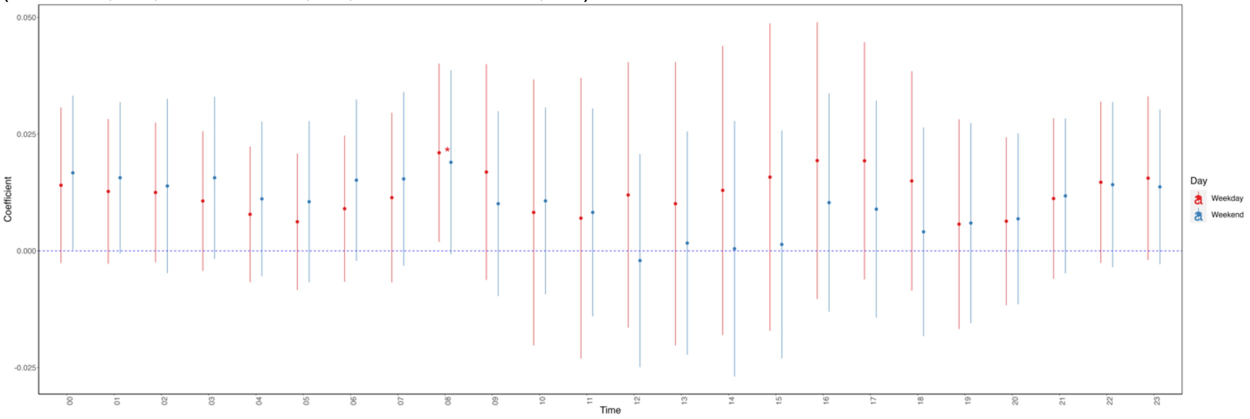

Culture

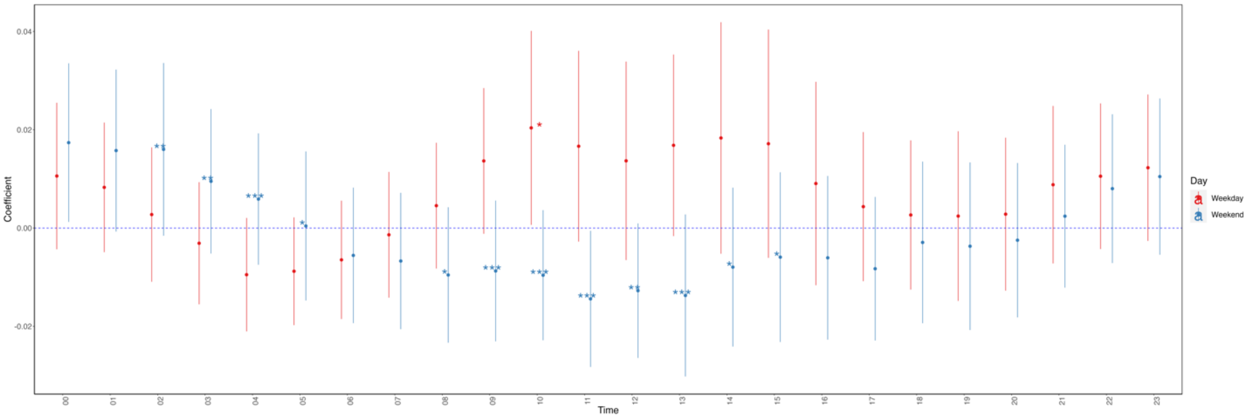

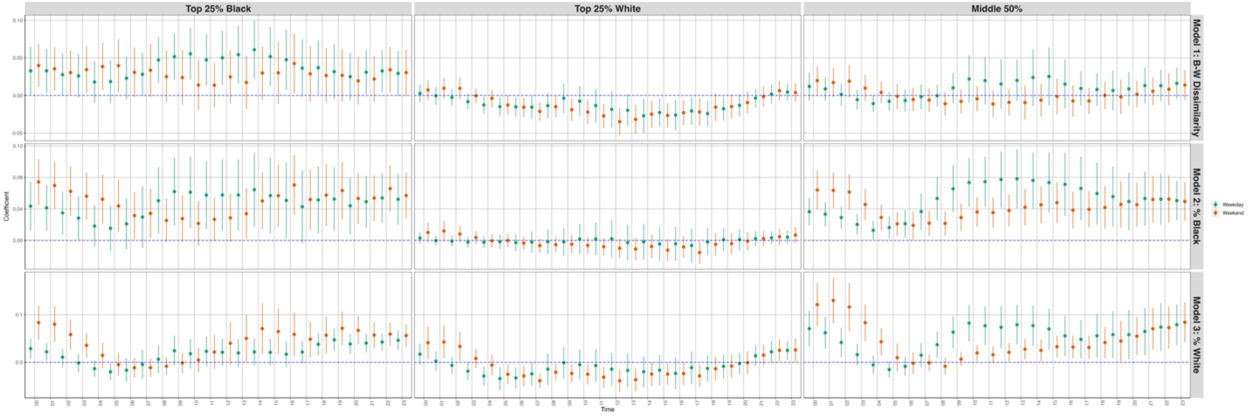

Models without public transportation stops as an independent variable, with three additional metropolitan areas (Louisville, KY; New Orleans, LA; and San Antonio, TX)

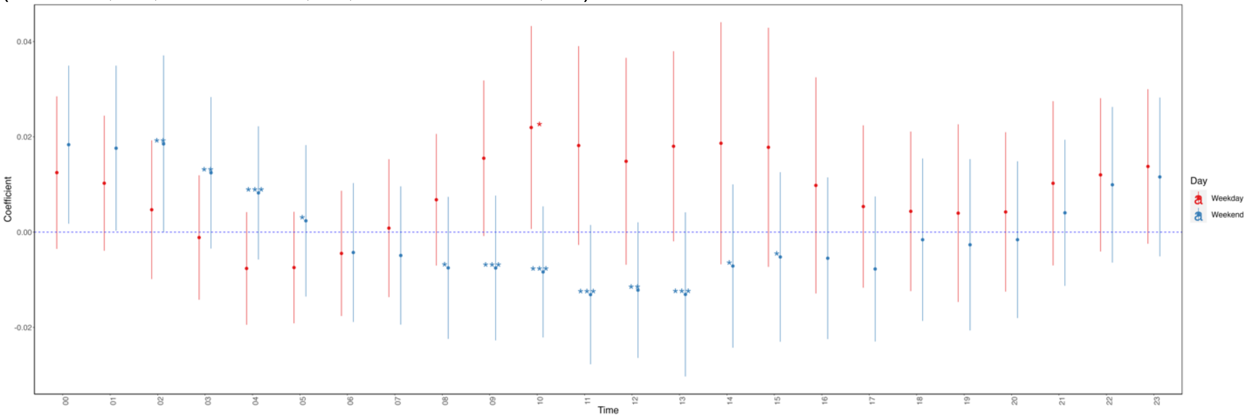

Colleges and Universities

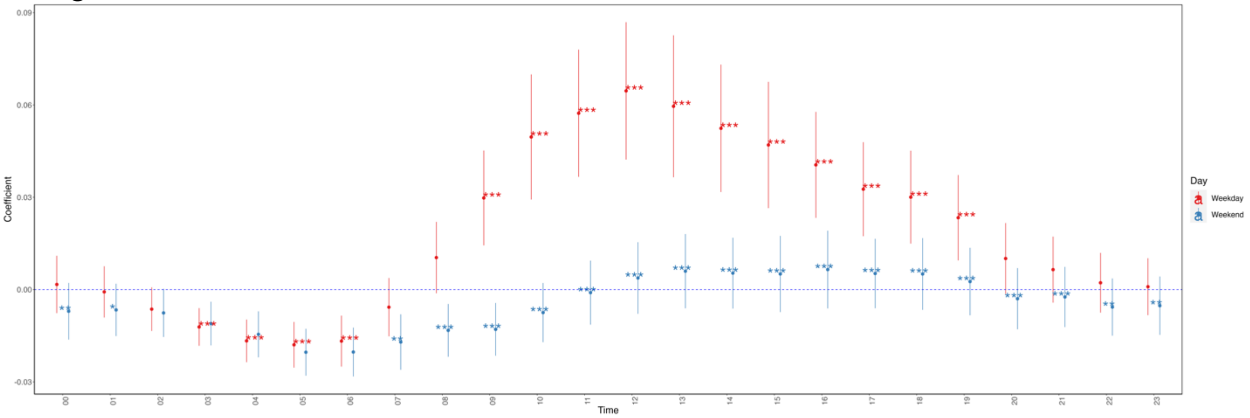

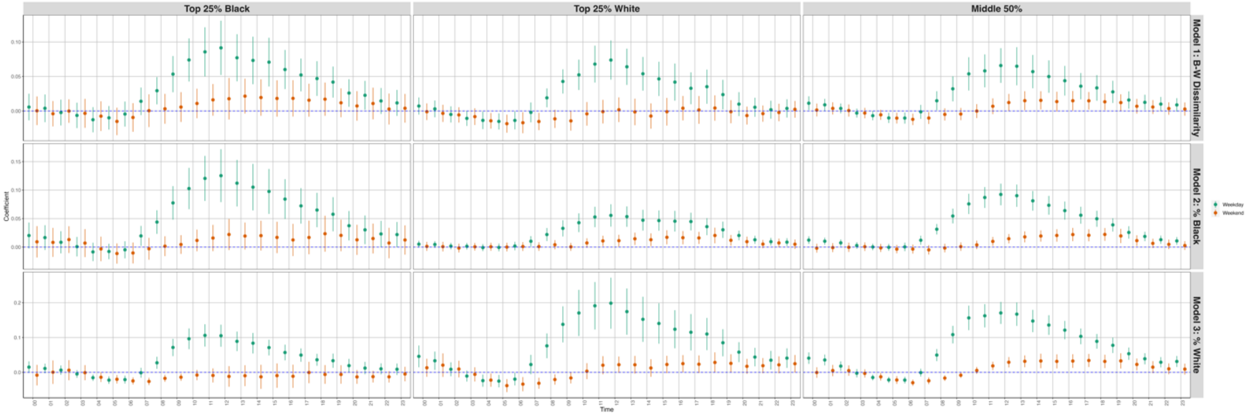

Models without public transportation stops as an independent variable, with three additional metropolitan areas (Louisville, KY; New Orleans, LA; and San Antonio, TX)

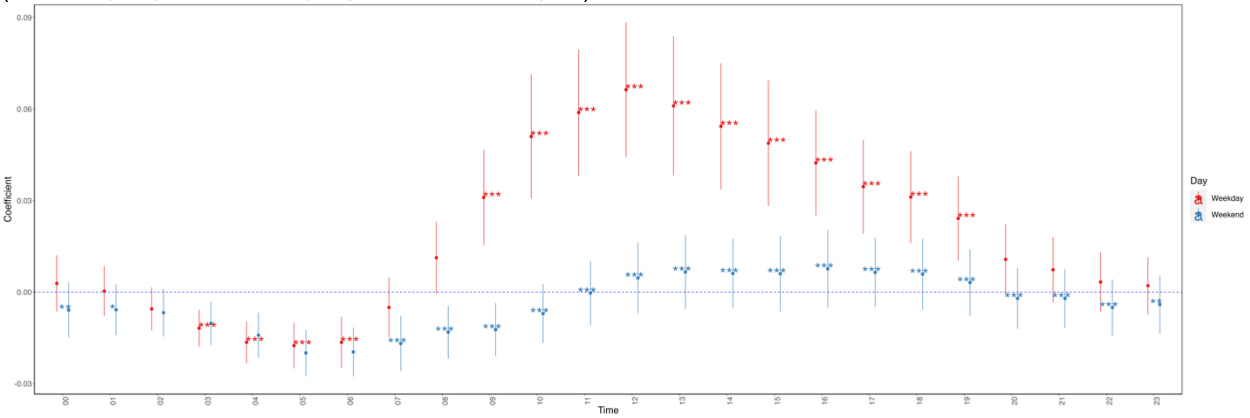

Construction

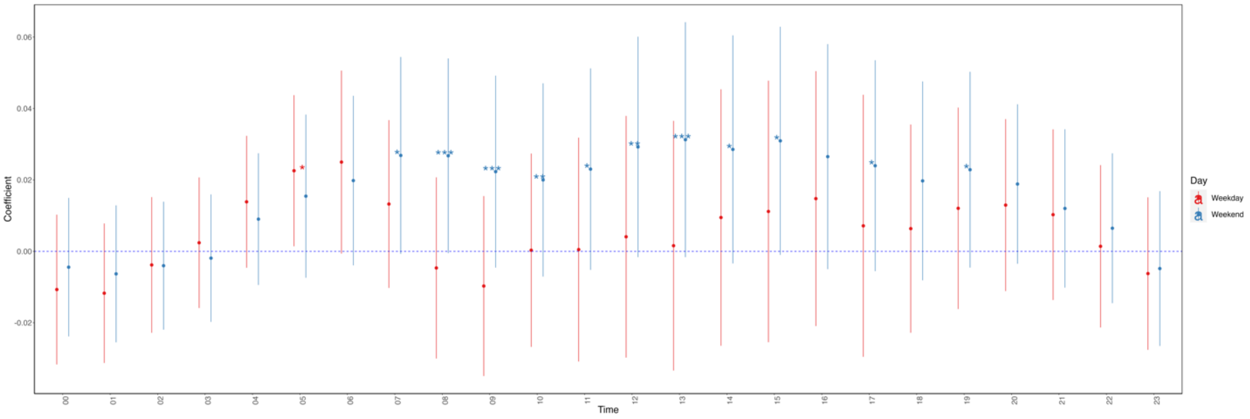

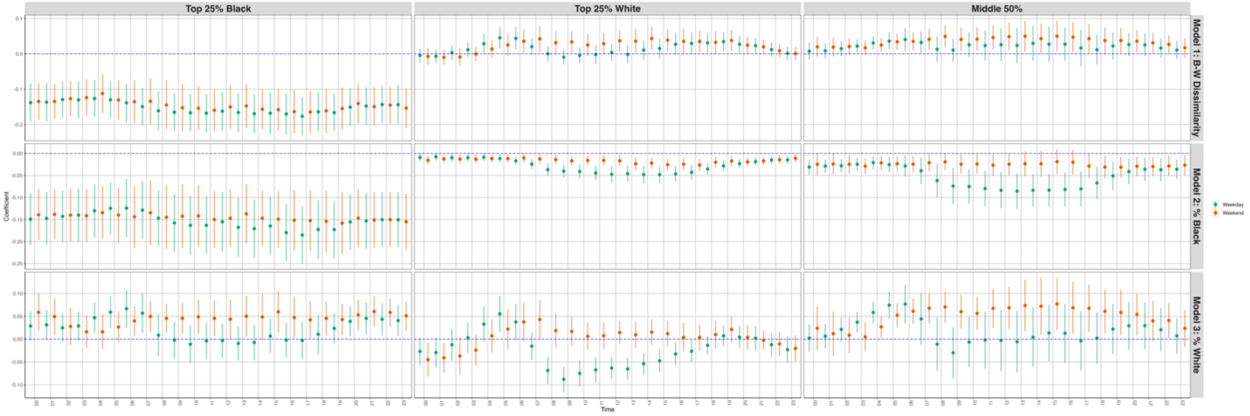

Models without public transportation stops as an independent variable, with three additional metropolitan areas (Louisville, KY; New Orleans, LA; and San Antonio, TX)

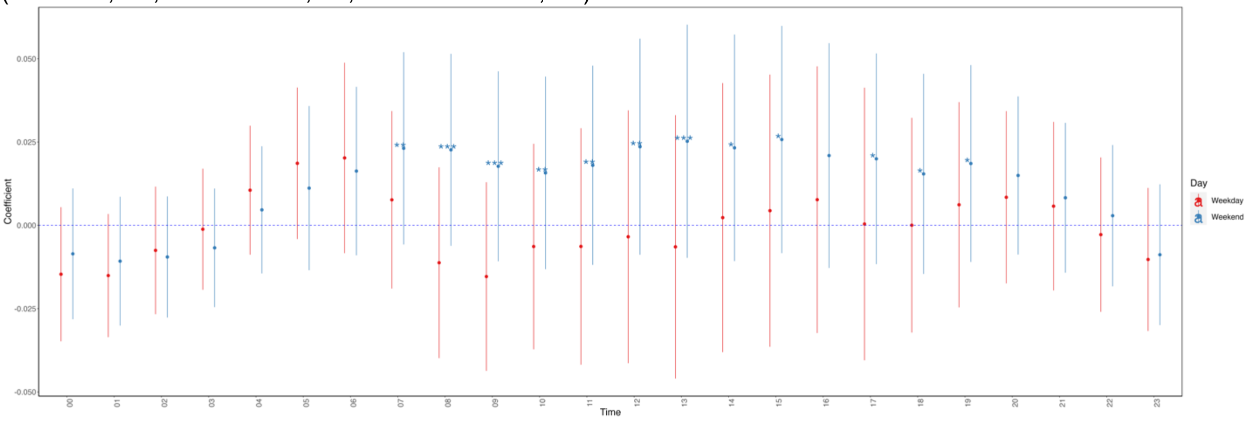

Finance and Insurance

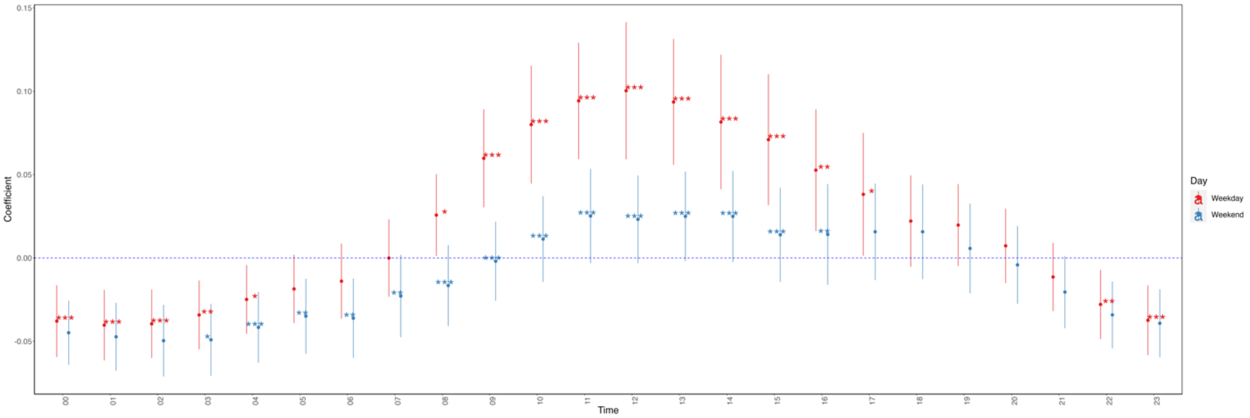

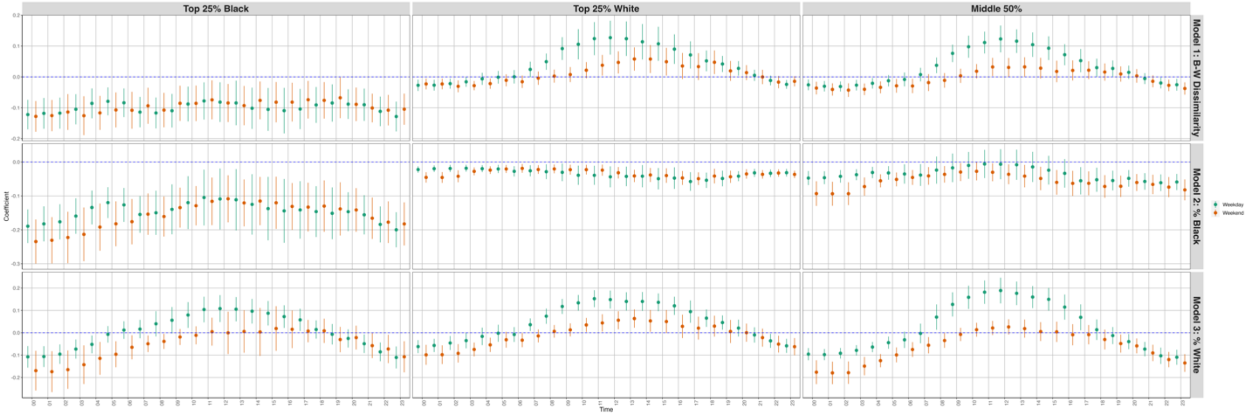

Models without public transportation stops as an independent variable, with three additional metropolitan areas (Louisville, KY; New Orleans, LA; and San Antonio, TX)

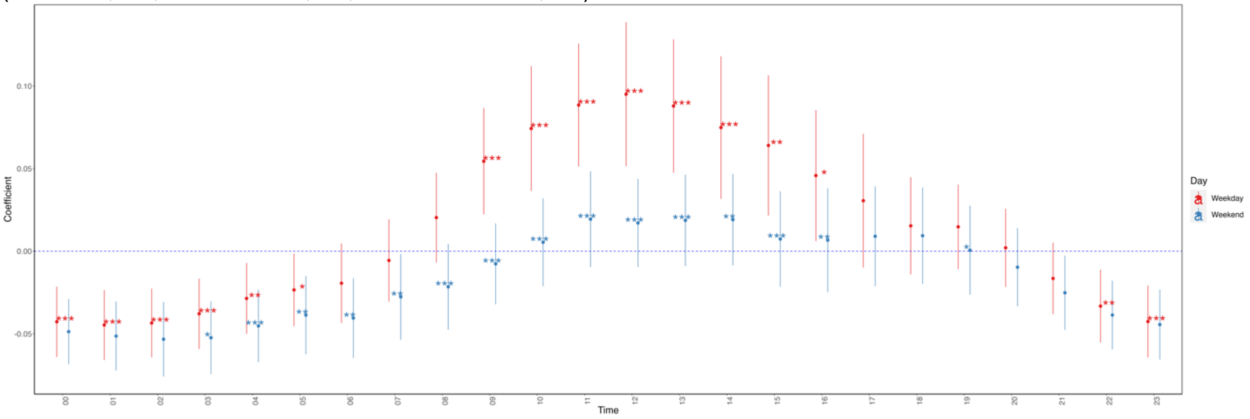

Food Retail

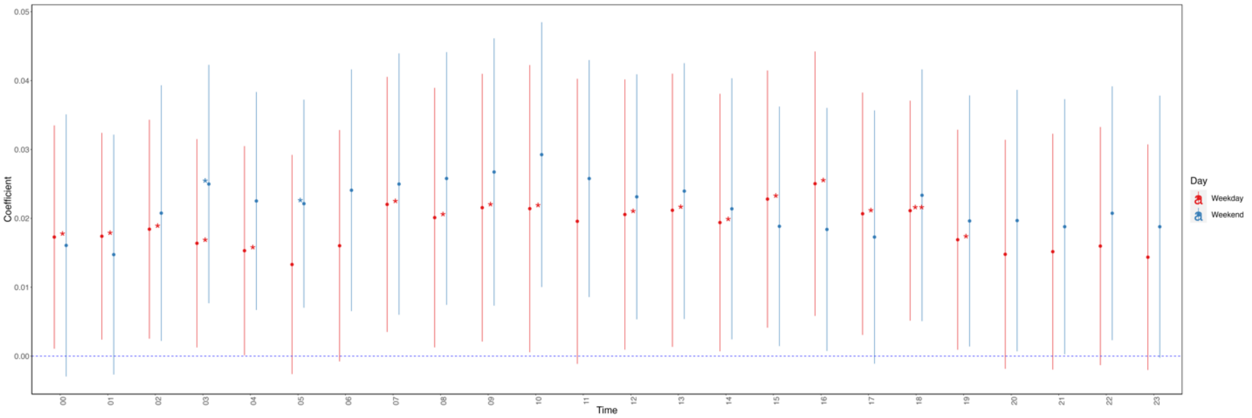

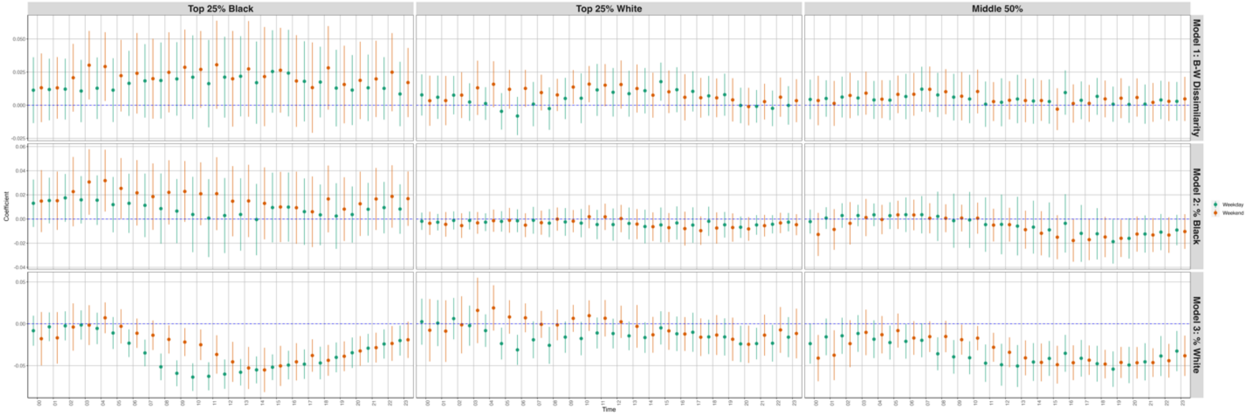

Models without public transportation stops as an independent variable, with three additional metropolitan areas (Louisville, KY; New Orleans, LA; and San Antonio, TX)

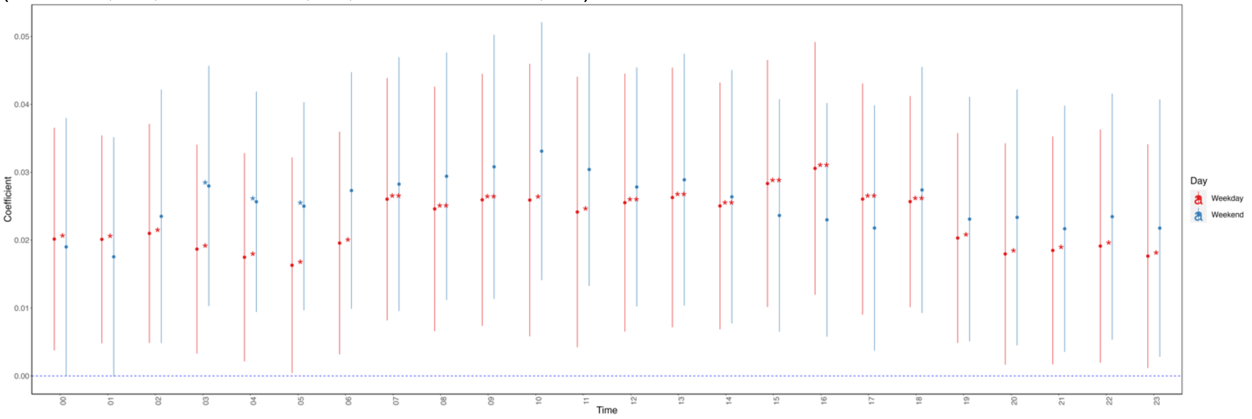

Health Care

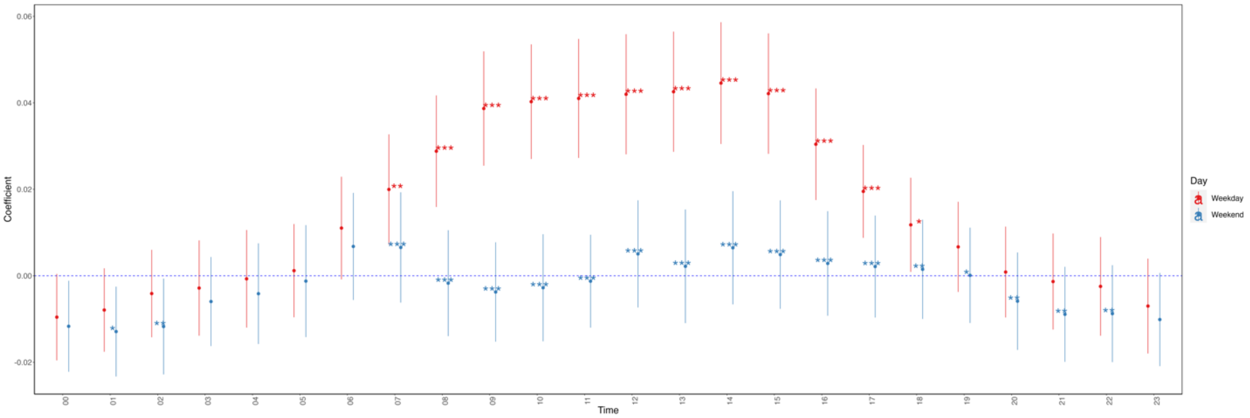

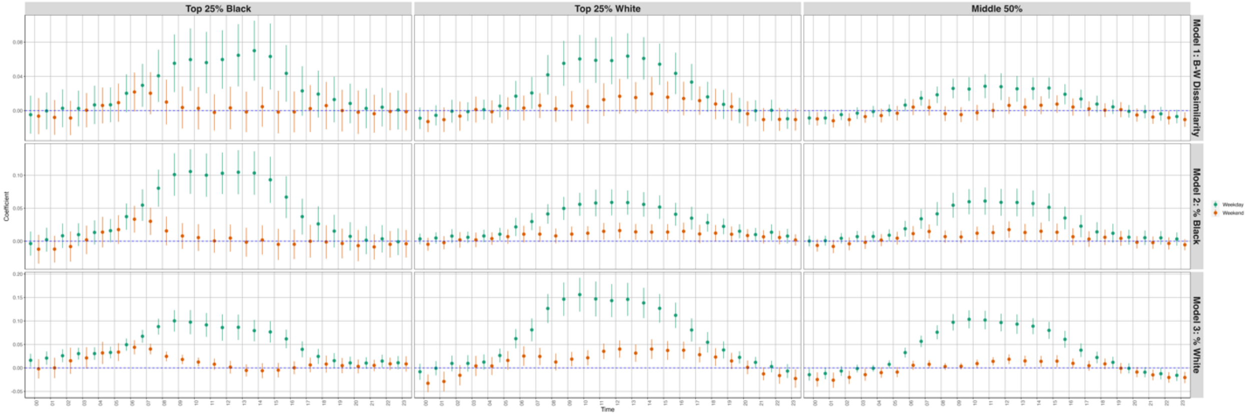

Models without public transportation stops as an independent variable, with three additional metropolitan areas (Louisville, KY; New Orleans, LA; and San Antonio, TX)

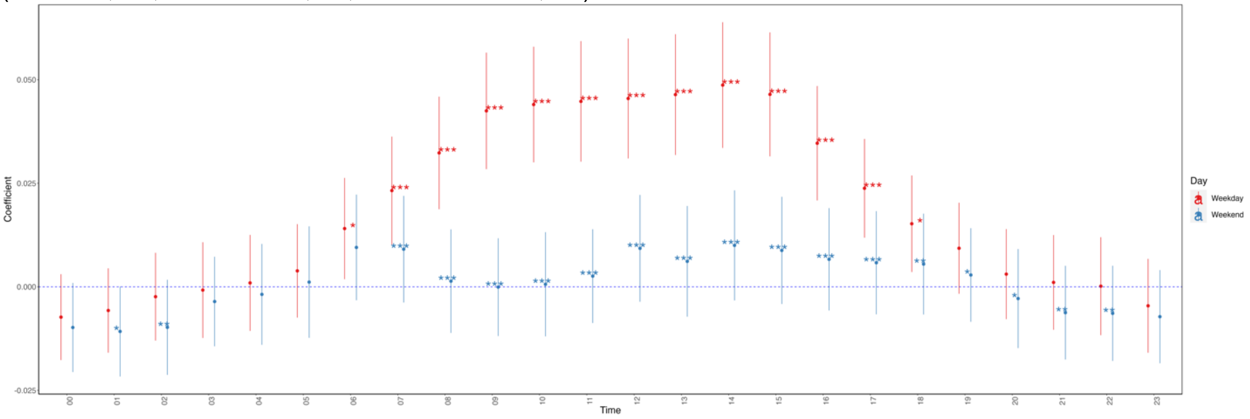

Home Retail

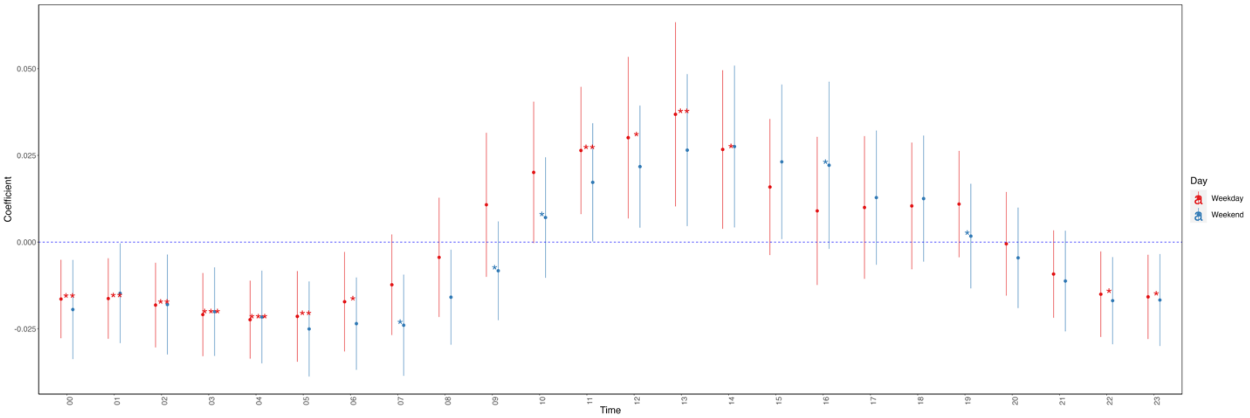

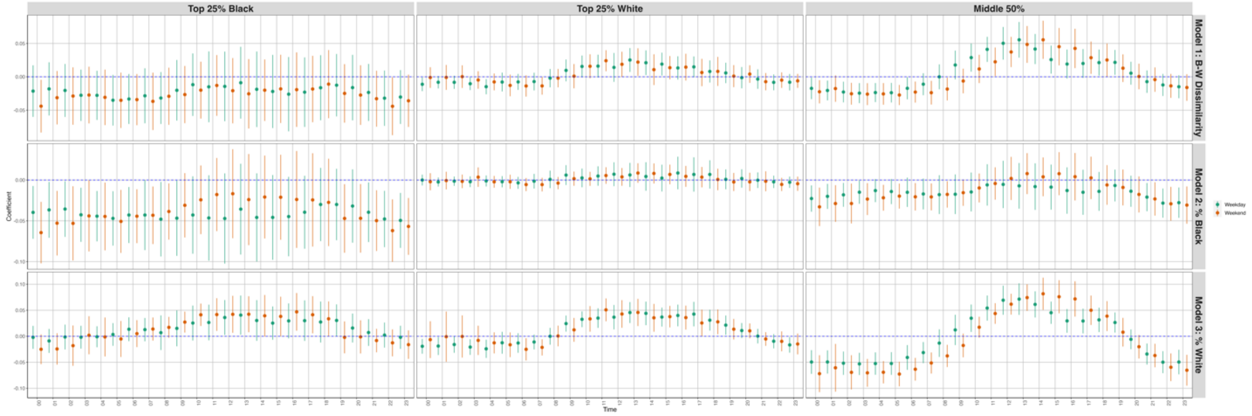

Models without public transportation stops as an independent variable, with three additional metropolitan areas (Louisville, KY; New Orleans, LA; and San Antonio, TX)

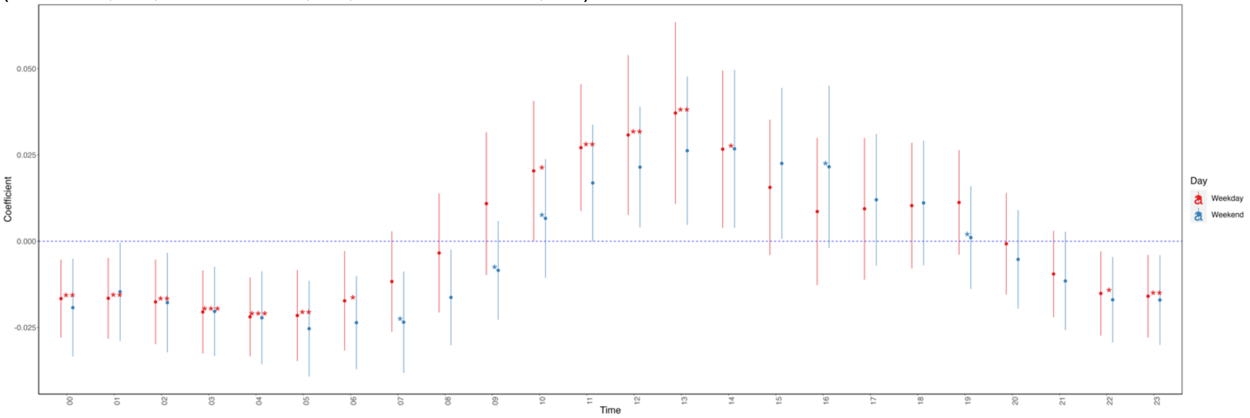

Information

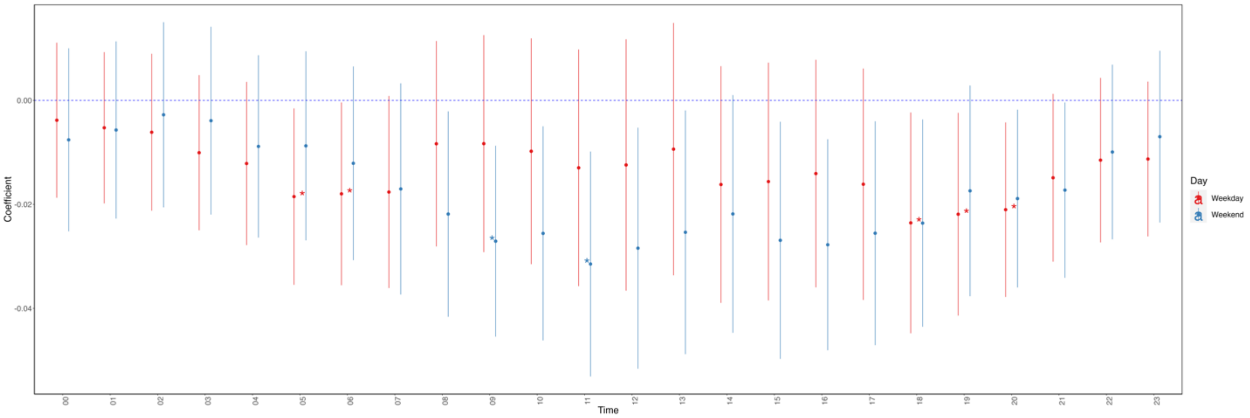

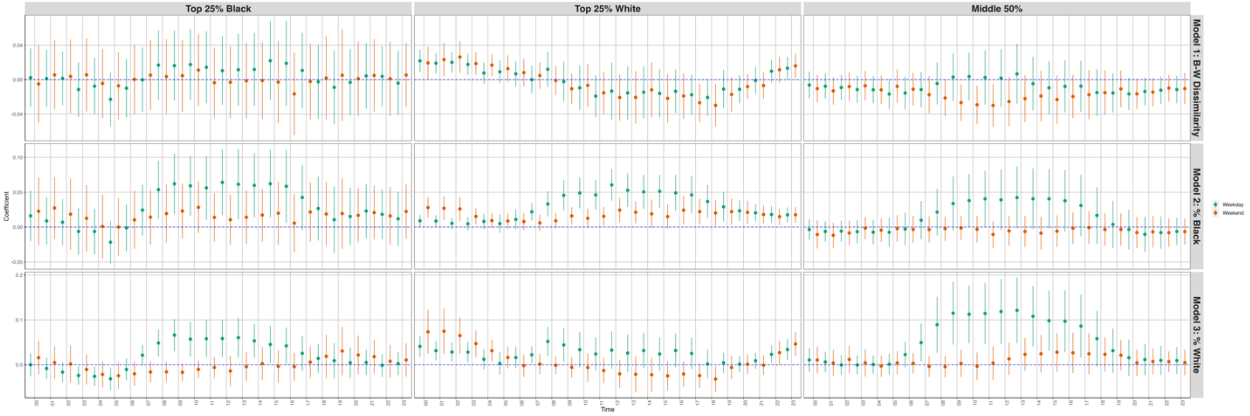

Models without public transportation stops as an independent variable, with three additional metropolitan areas (Louisville, KY; New Orleans, LA; and San Antonio, TX)

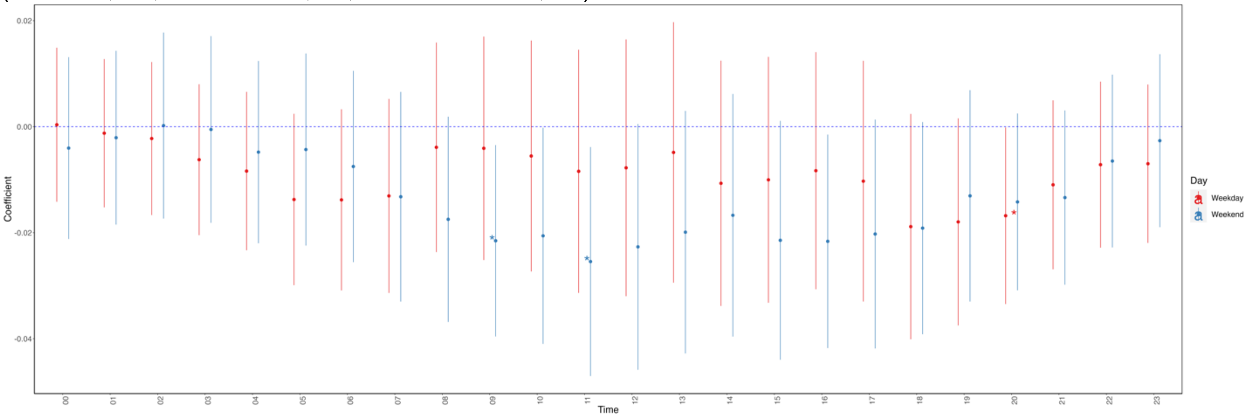

K-12 Schools

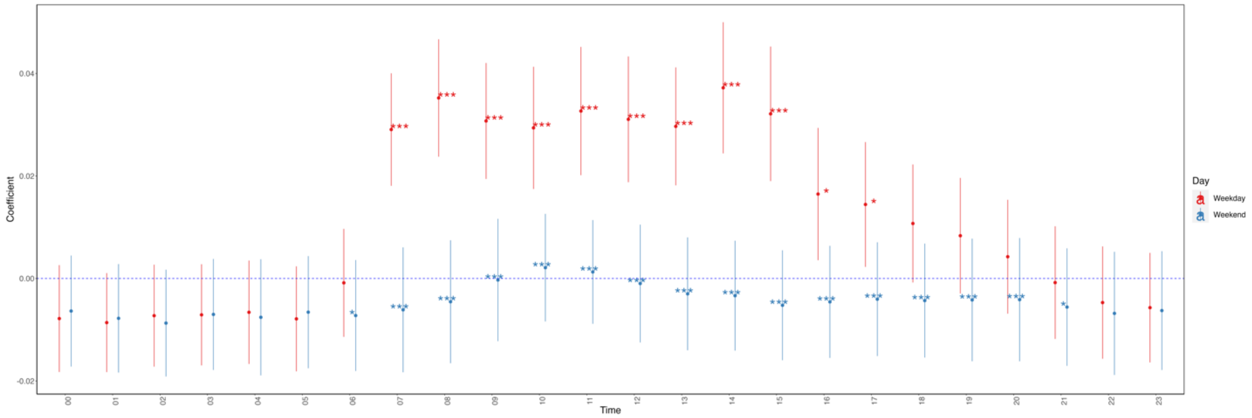

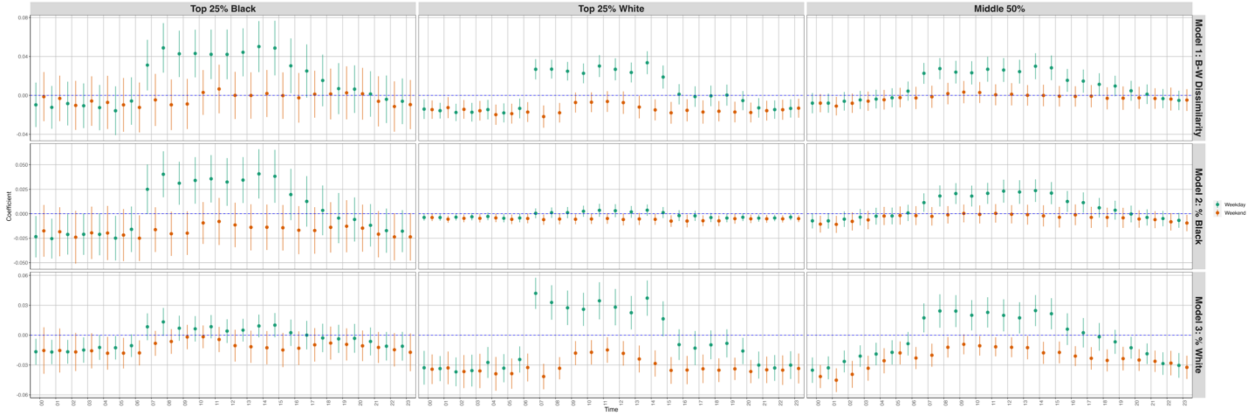

Models without public transportation stops as an independent variable, with three additional metropolitan areas (Louisville, KY; New Orleans, LA; and San Antonio, TX)

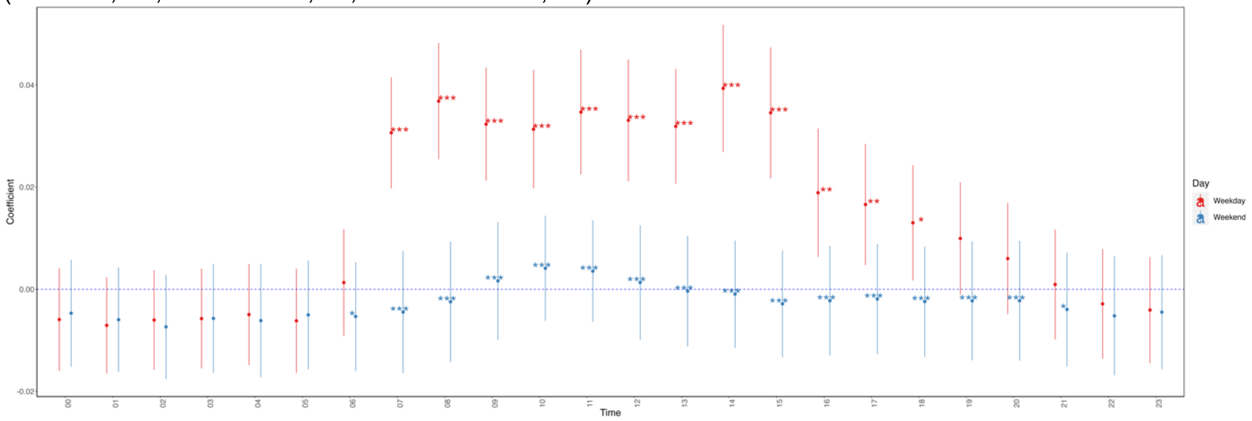

Management of Companies and Enterprises

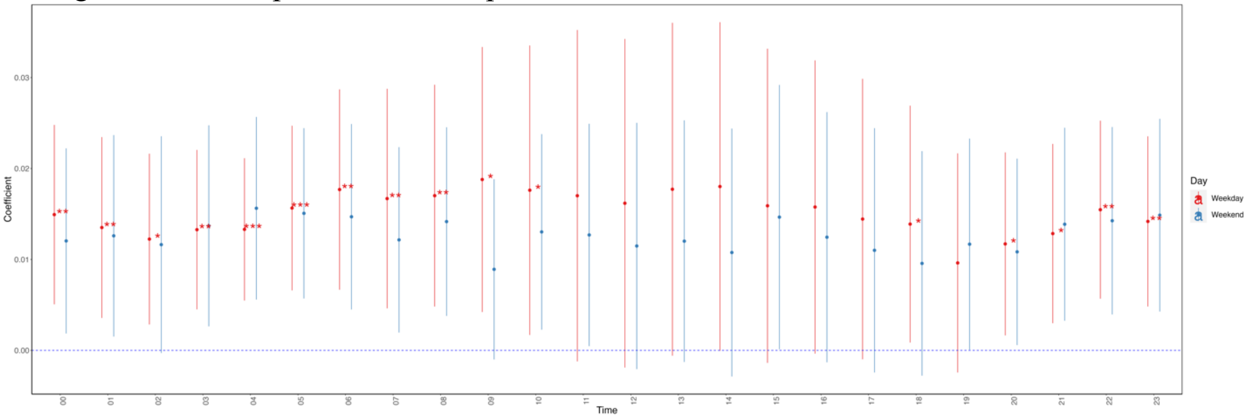

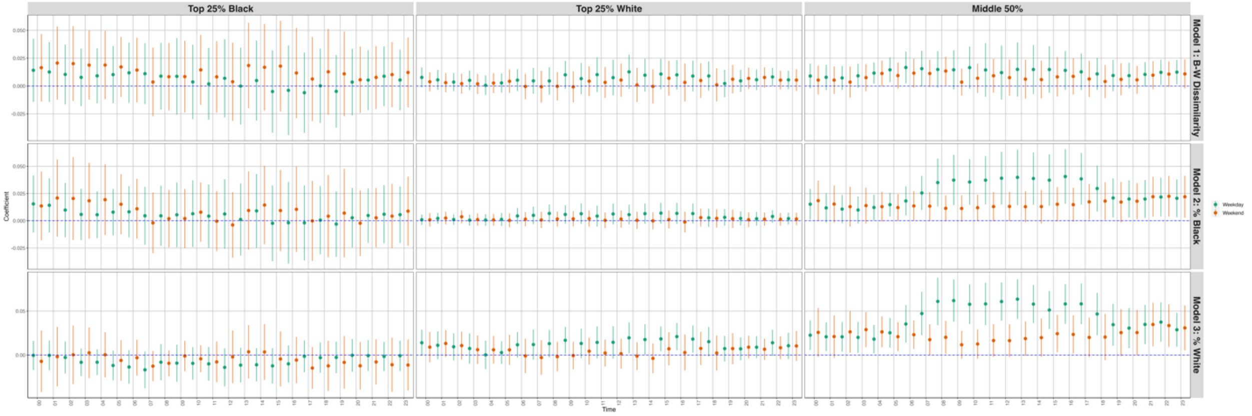

Models without public transportation stops as an independent variable, with three additional metropolitan areas (Louisville, KY; New Orleans, LA; and San Antonio, TX)

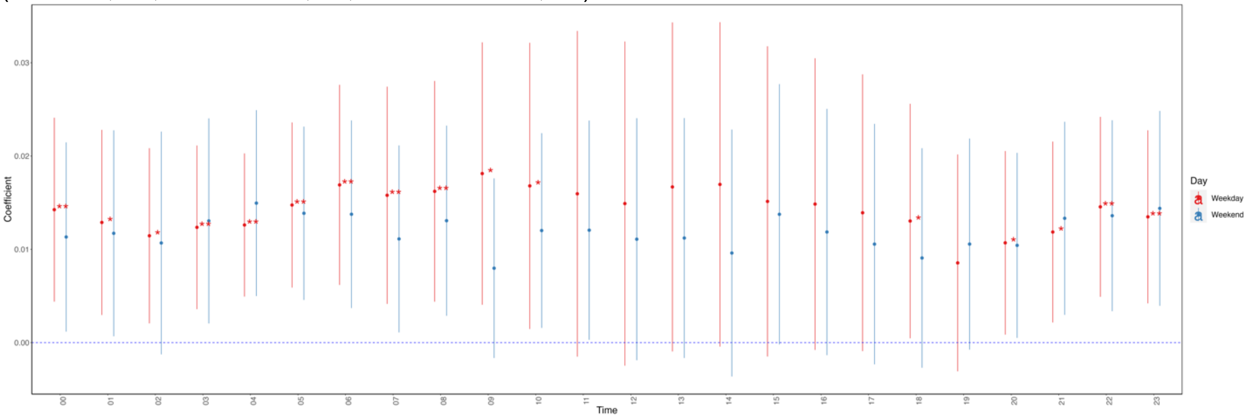

Manufacturing

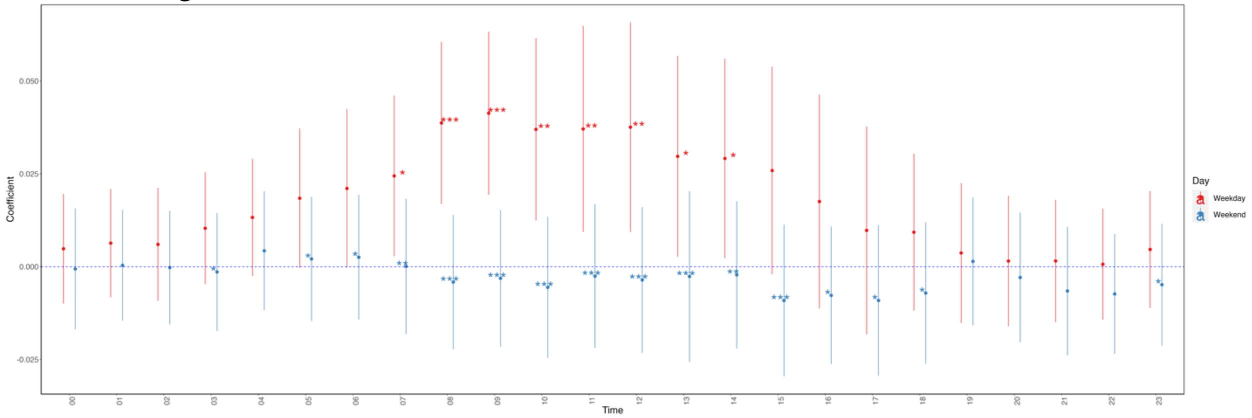

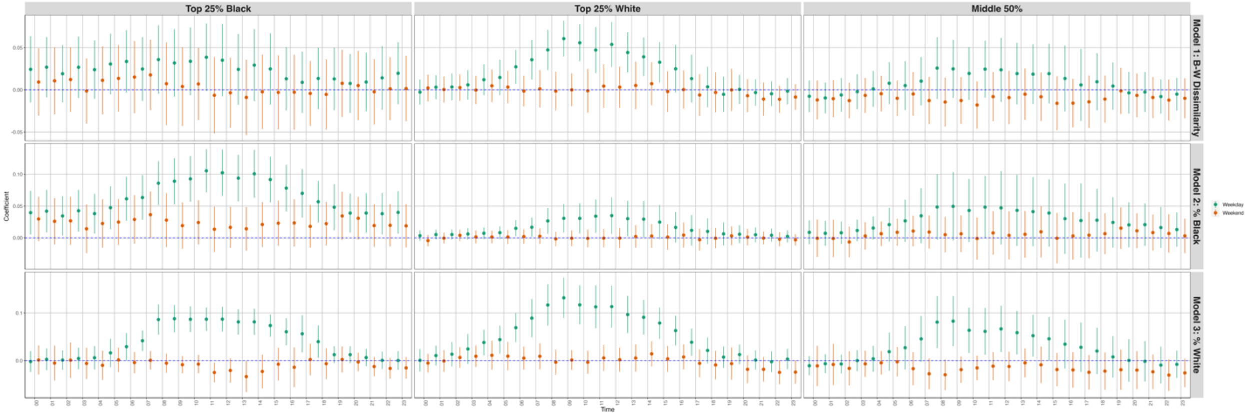

Models without public transportation stops as an independent variable, with three additional metropolitan areas (Louisville, KY; New Orleans, LA; and San Antonio, TX)

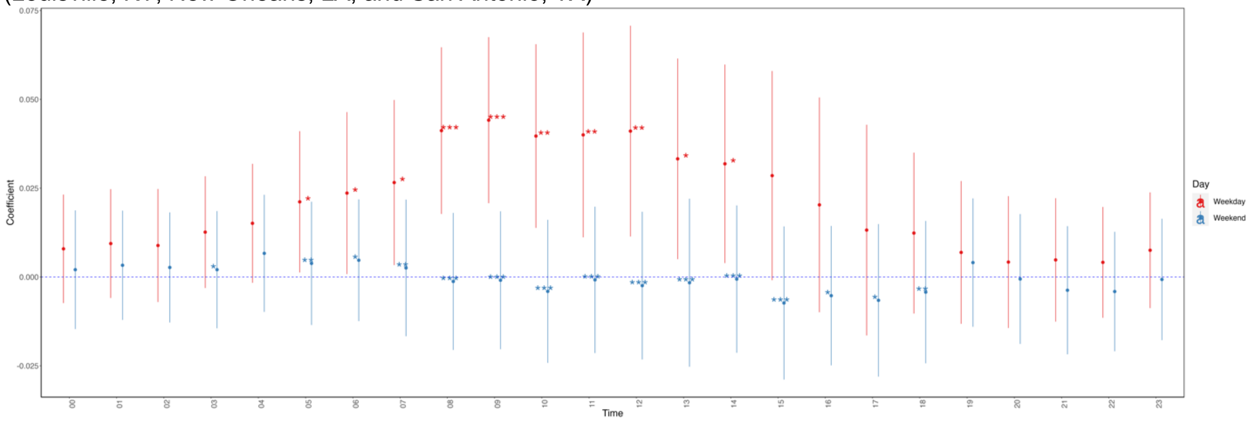

Median Household Income by Census Tract

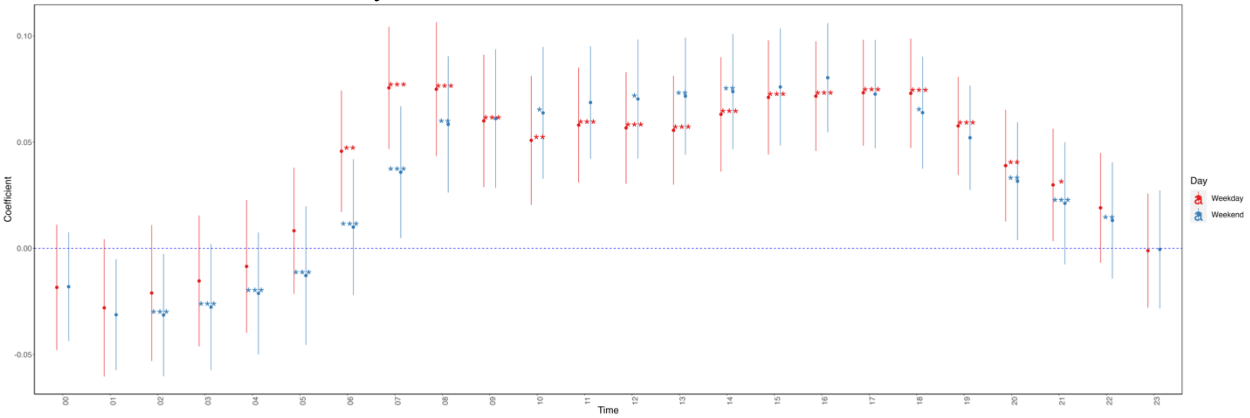

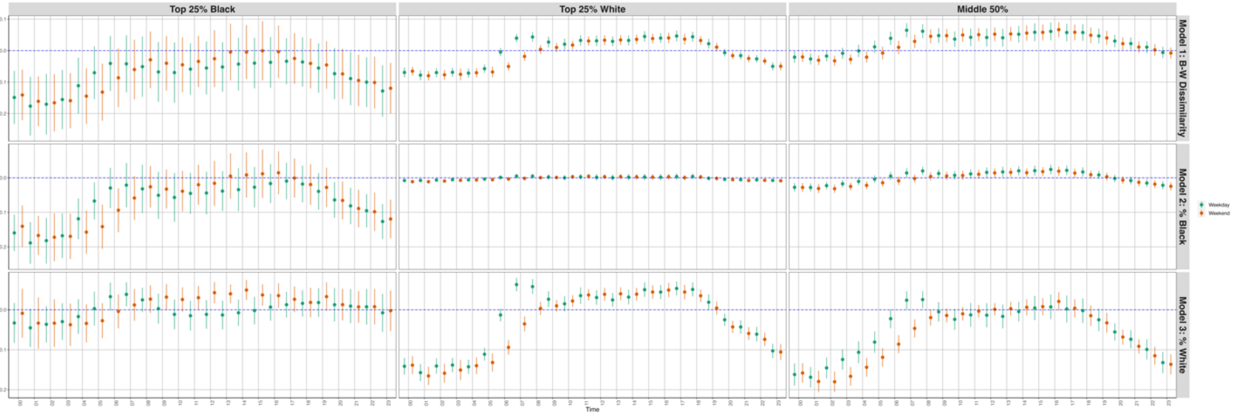

Models without public transportation stops as an independent variable, with three additional metropolitan areas (Louisville, KY; New Orleans, LA; and San Antonio, TX)

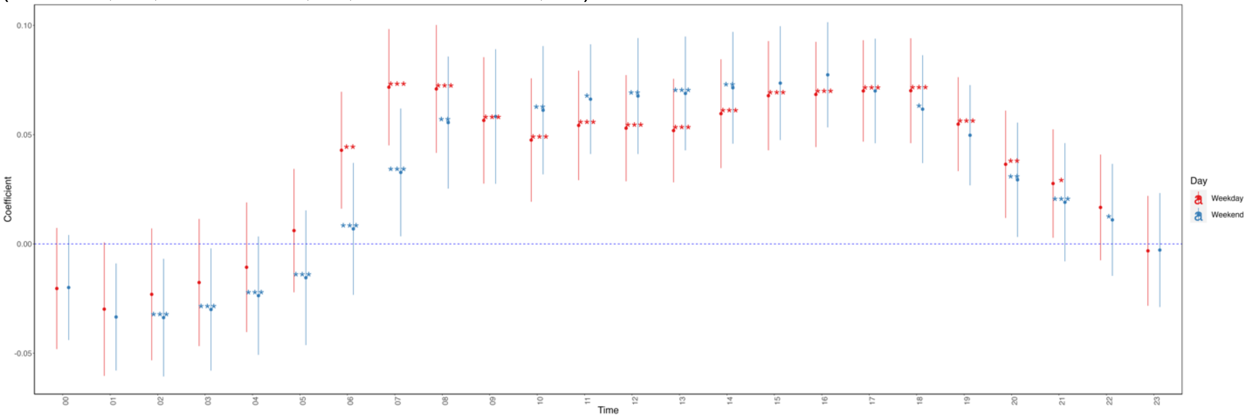

Mining, Quarrying, Oil, and Gas Extraction

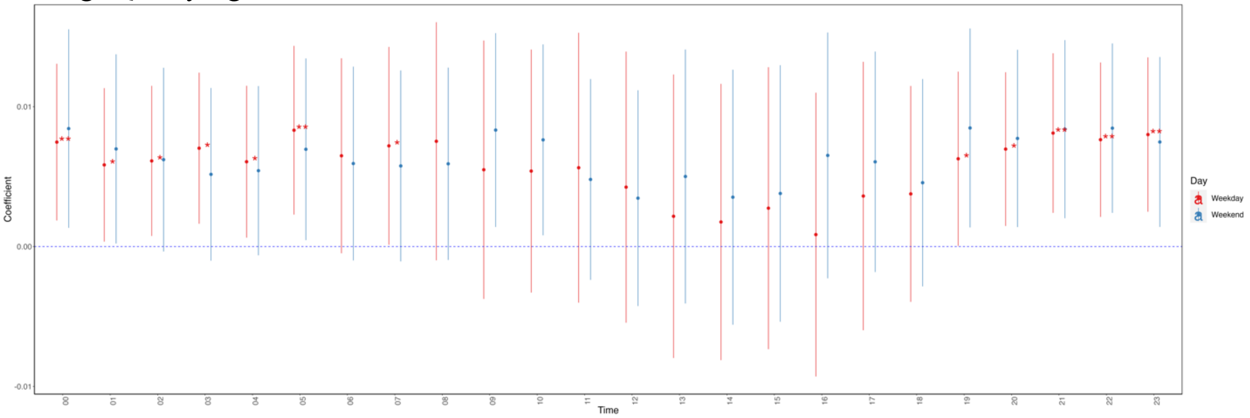

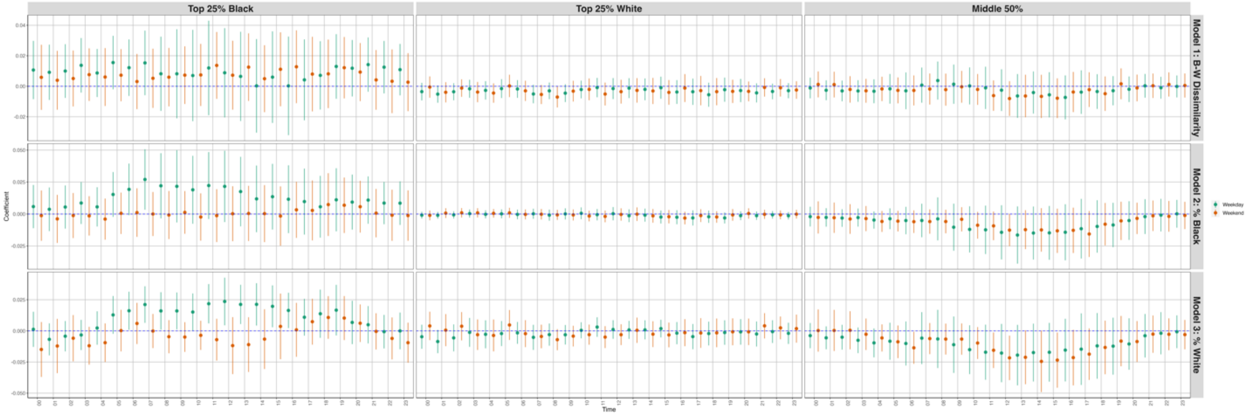

Models without public transportation stops as an independent variable, with three additional metropolitan areas (Louisville, KY; New Orleans, LA; and San Antonio, TX)

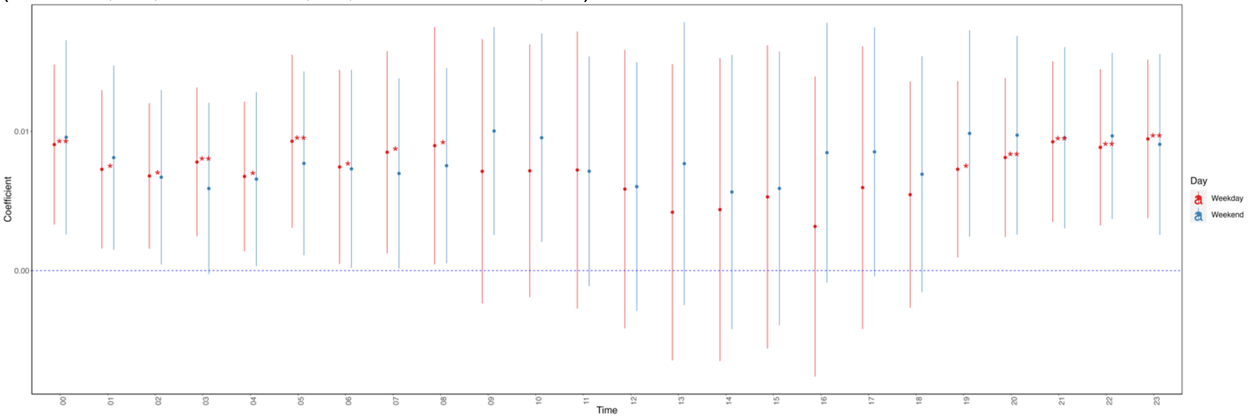

Other Accommodation

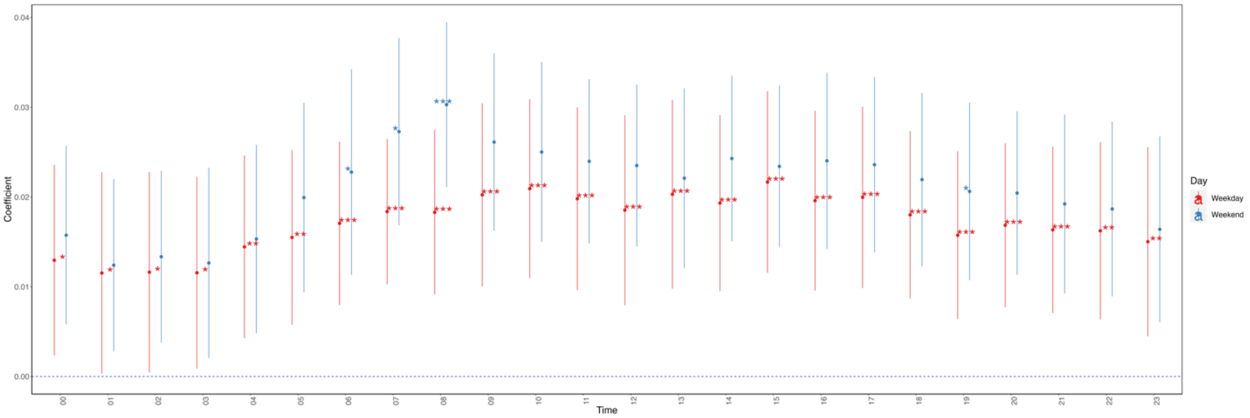

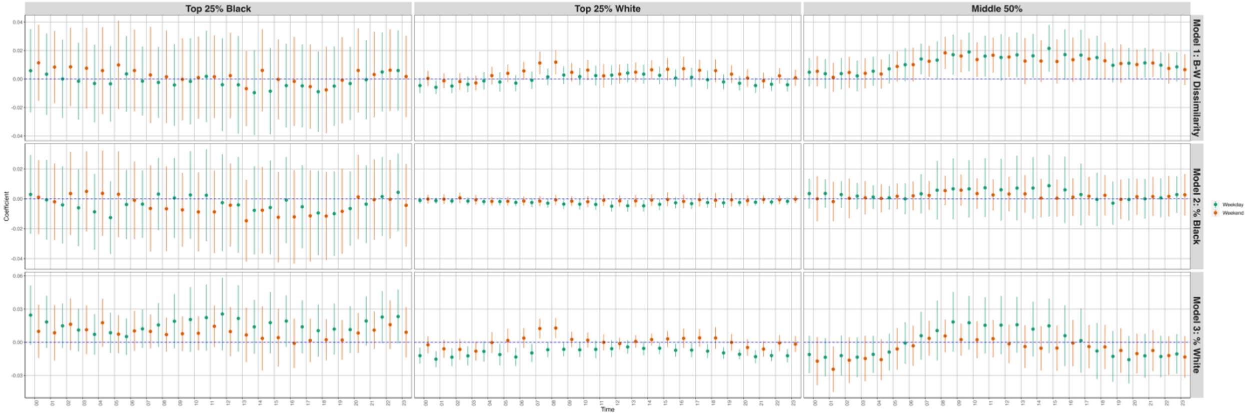

Models without public transportation stops as an independent variable, with three additional metropolitan areas (Louisville, KY; New Orleans, LA; and San Antonio, TX)

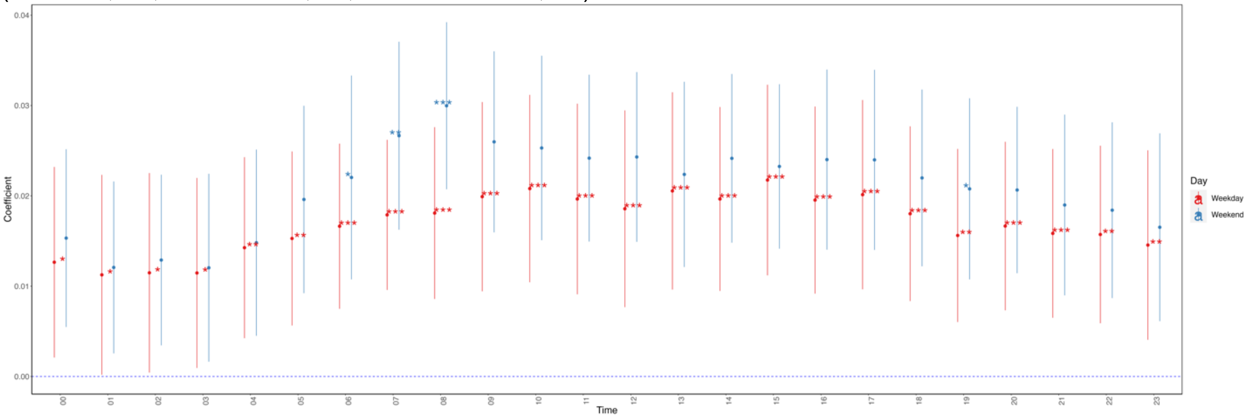

Other Restaurants and Bars

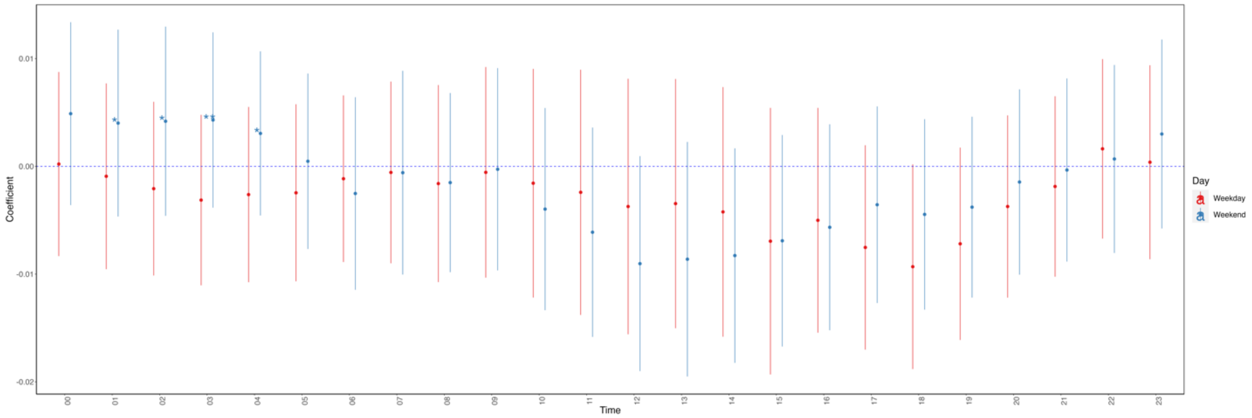

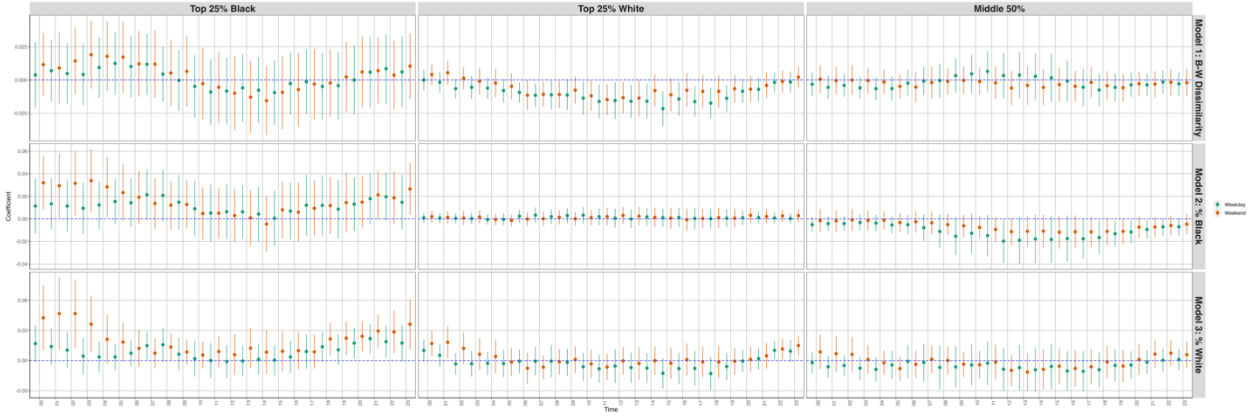

Models without public transportation stops as an independent variable, with three additional metropolitan areas (Louisville, KY; New Orleans, LA; and San Antonio, TX)

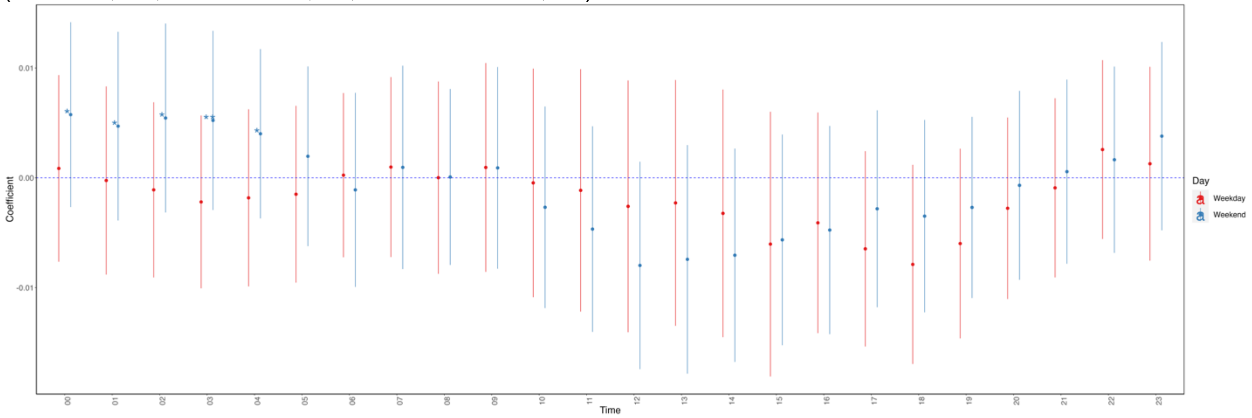

Other Culture

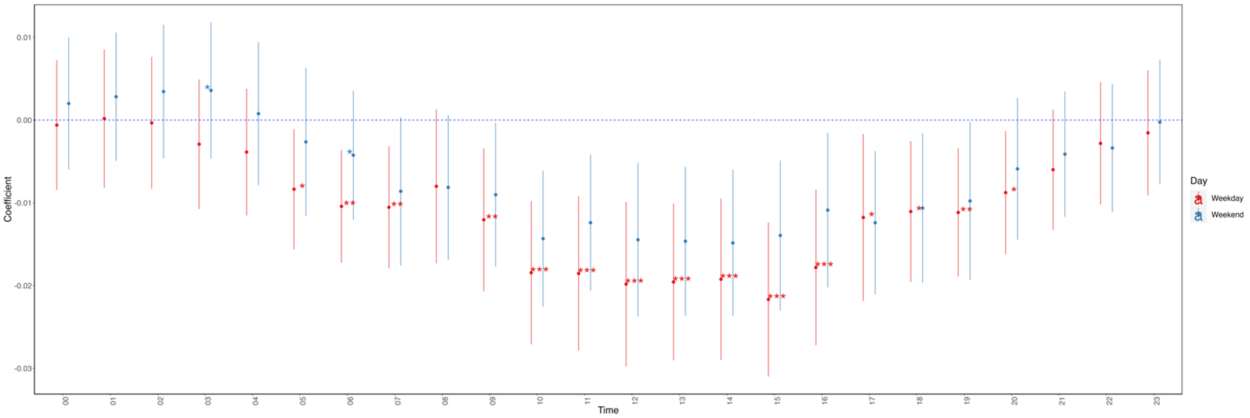

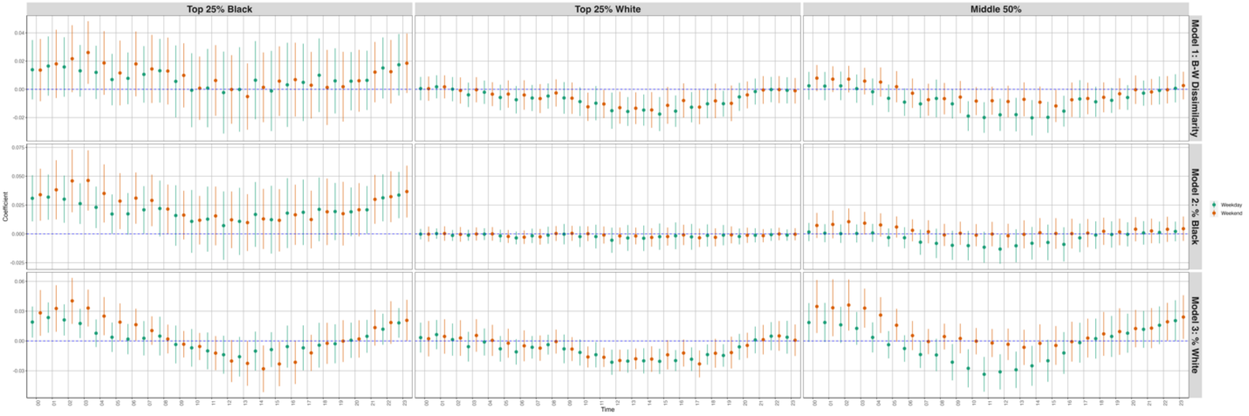

Models without public transportation stops as an independent variable, with three additional metropolitan areas (Louisville, KY; New Orleans, LA; and San Antonio, TX)

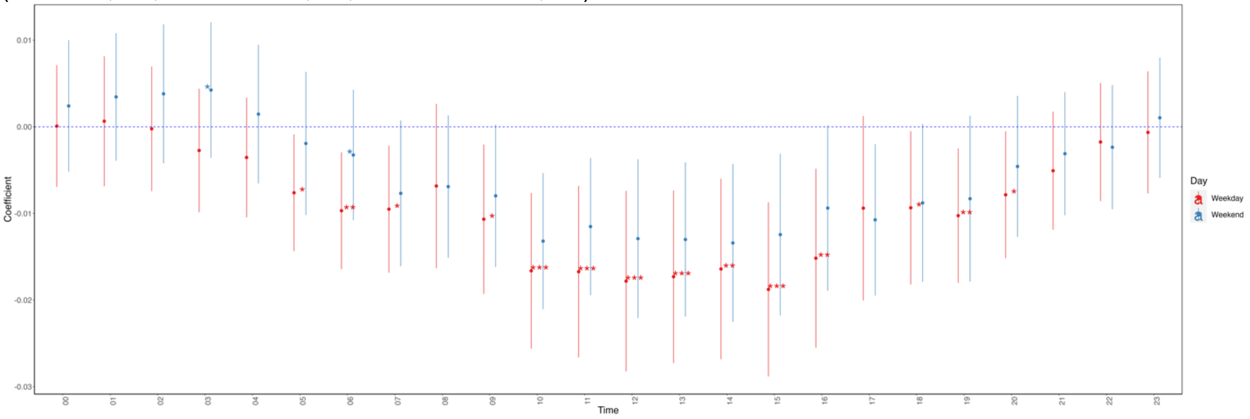

Other Retail

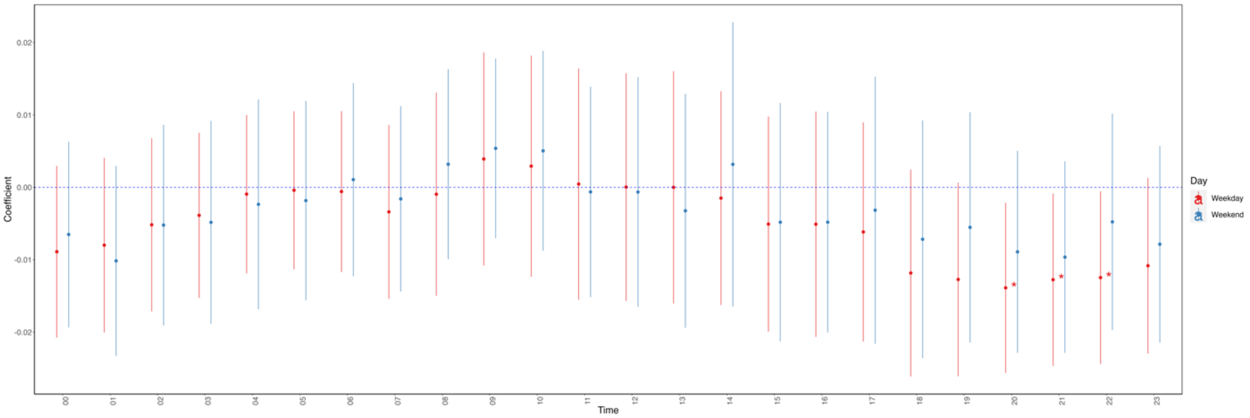

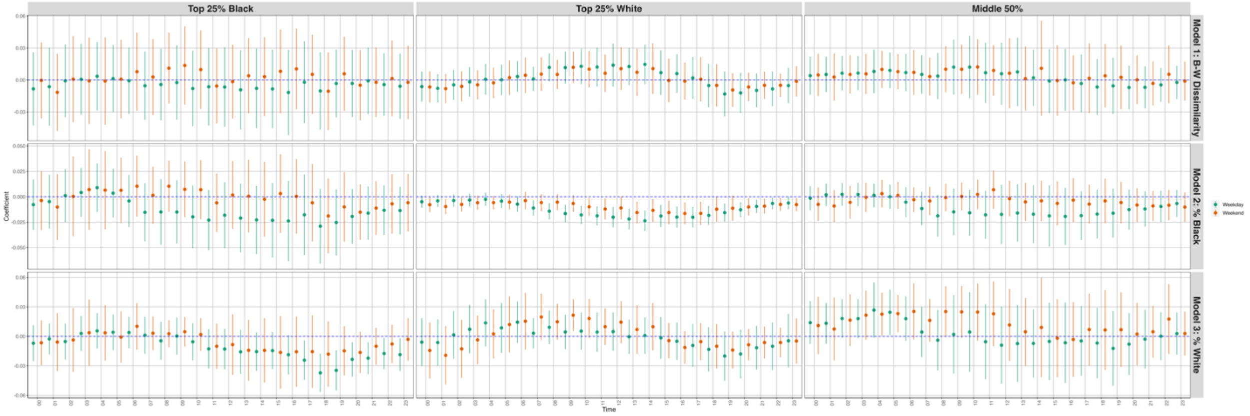

Models without public transportation stops as an independent variable, with three additional metropolitan areas (Louisville, KY; New Orleans, LA; and San Antonio, TX)

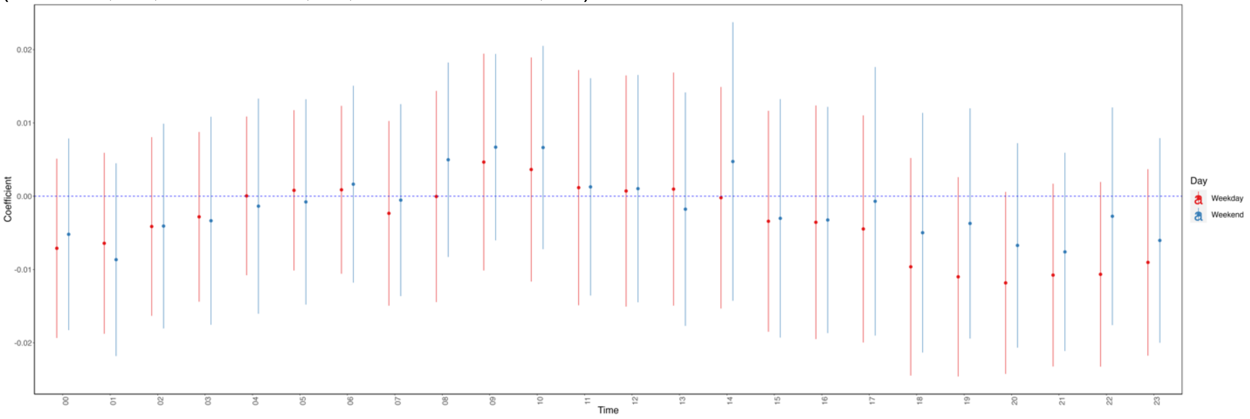

Other Schools

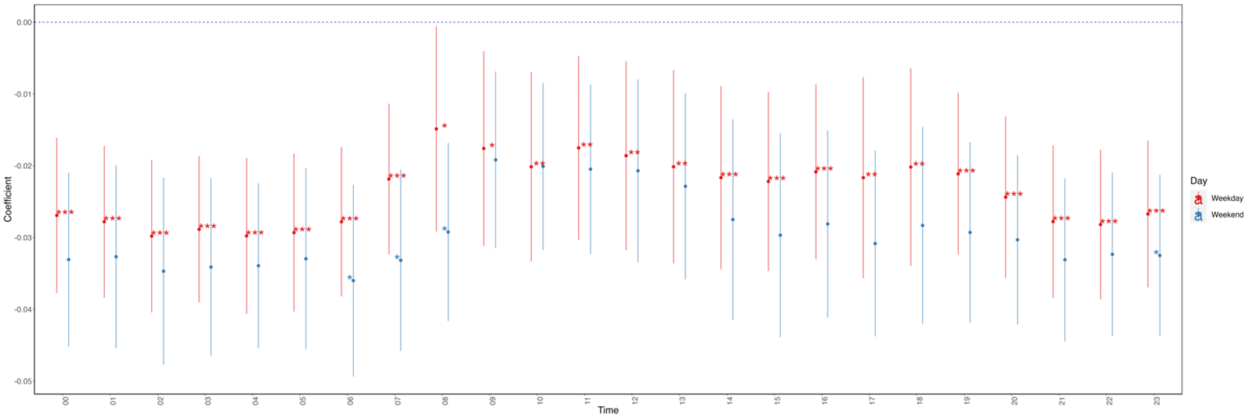

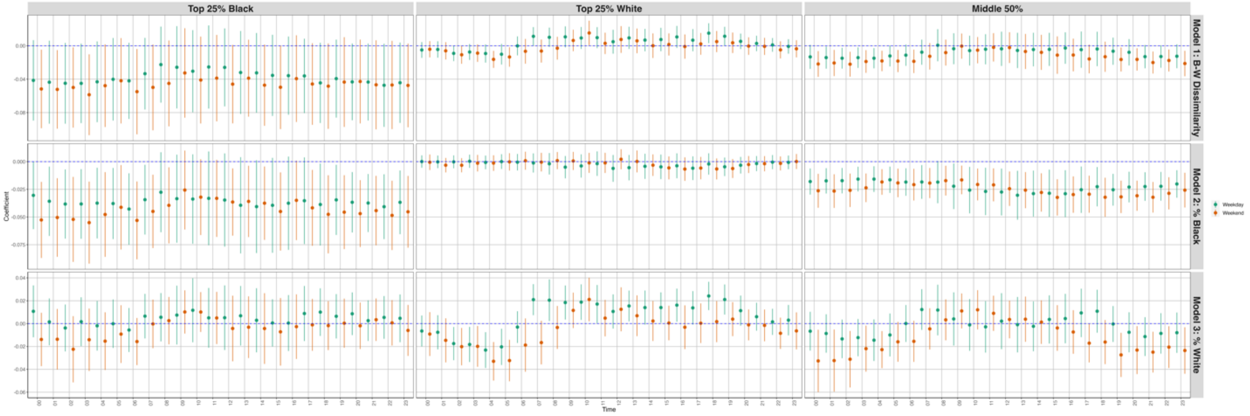

Models without public transportation stops as an independent variable, with three additional metropolitan areas (Louisville, KY; New Orleans, LA; and San Antonio, TX)

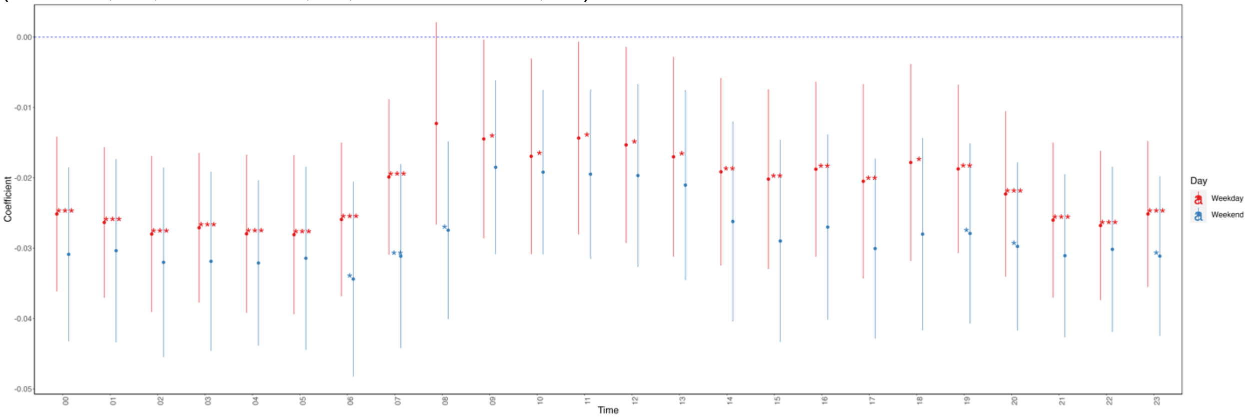

Other Services

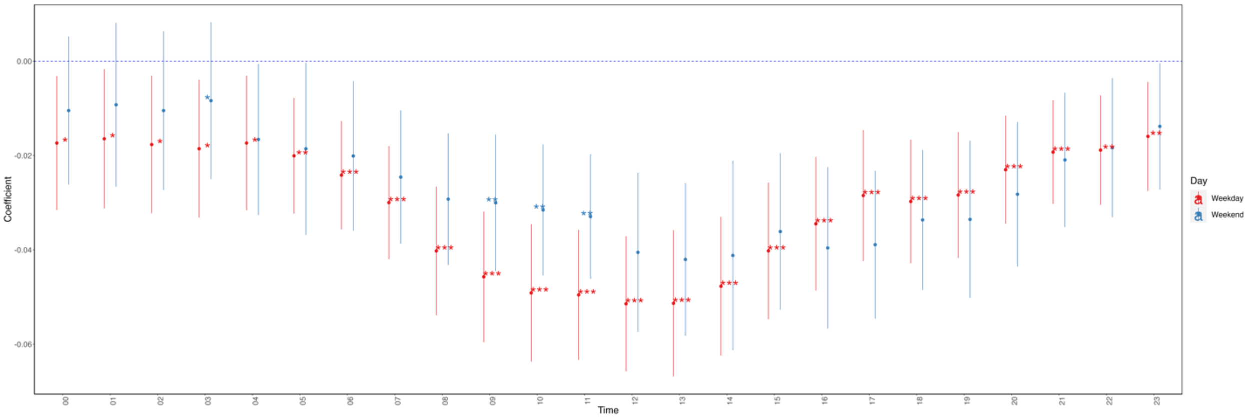

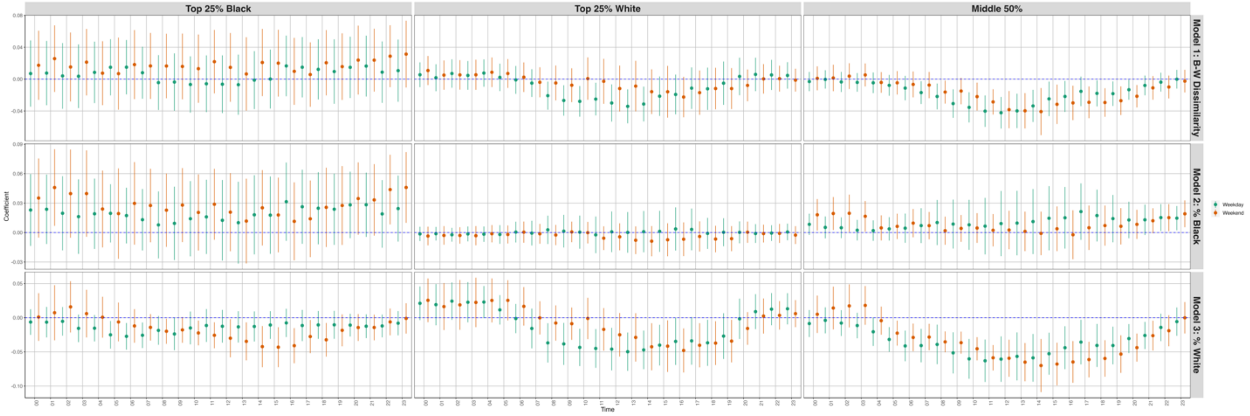

Models without public transportation stops as an independent variable, with three additional metropolitan areas (Louisville, KY; New Orleans, LA; and San Antonio, TX)

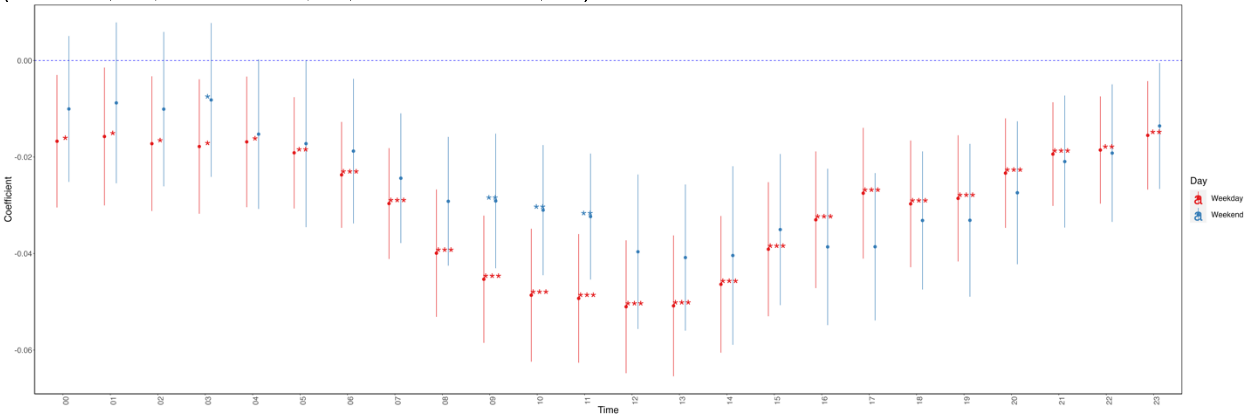

Personal Care

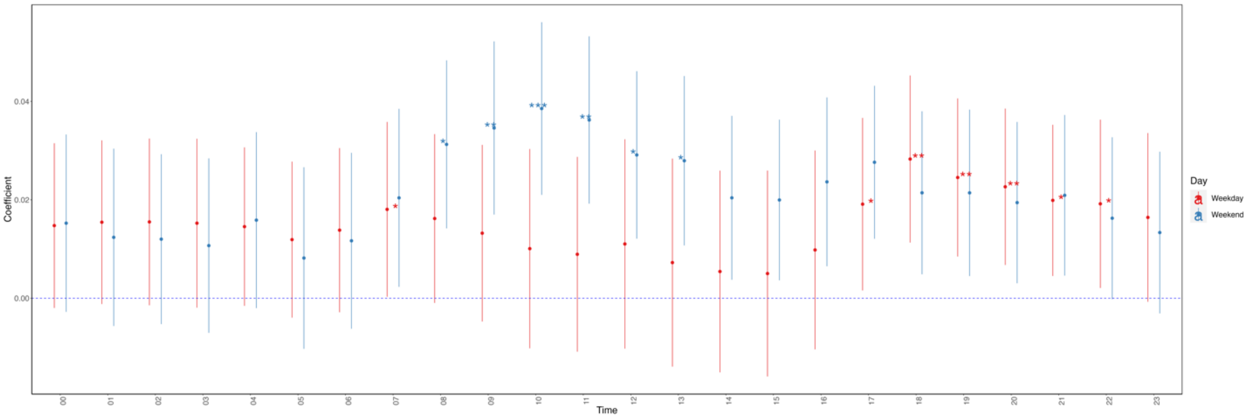

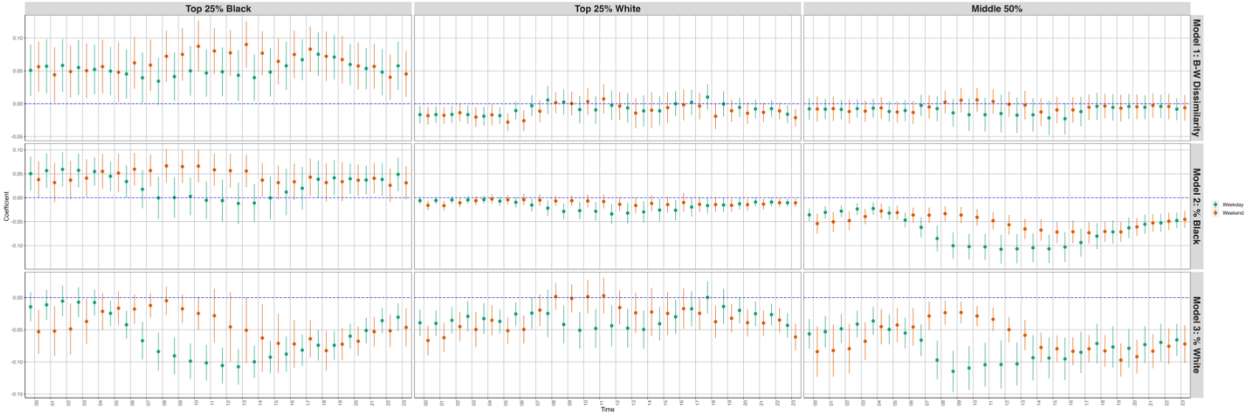

Models without public transportation stops as an independent variable, with three additional metropolitan areas (Louisville, KY; New Orleans, LA; and San Antonio, TX)

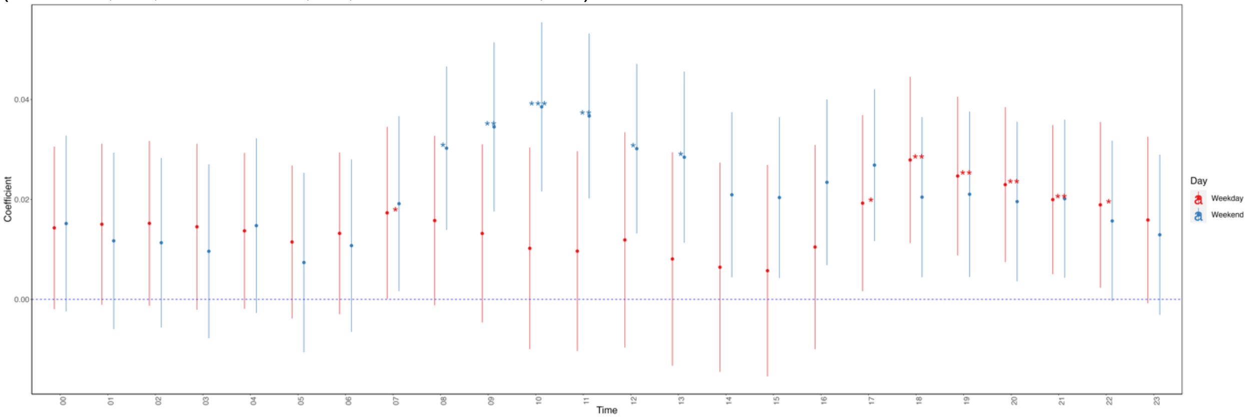

% Black Residents by Census Tract

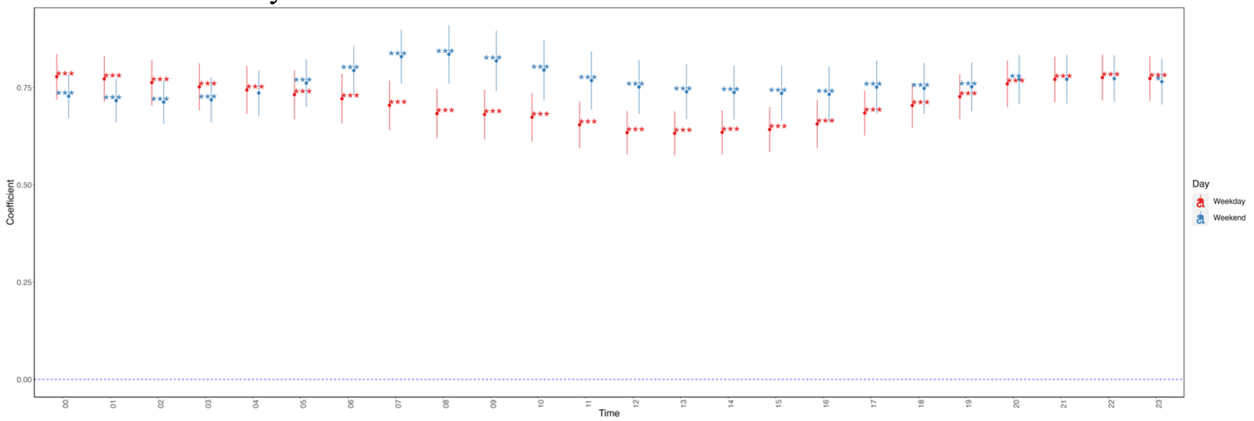

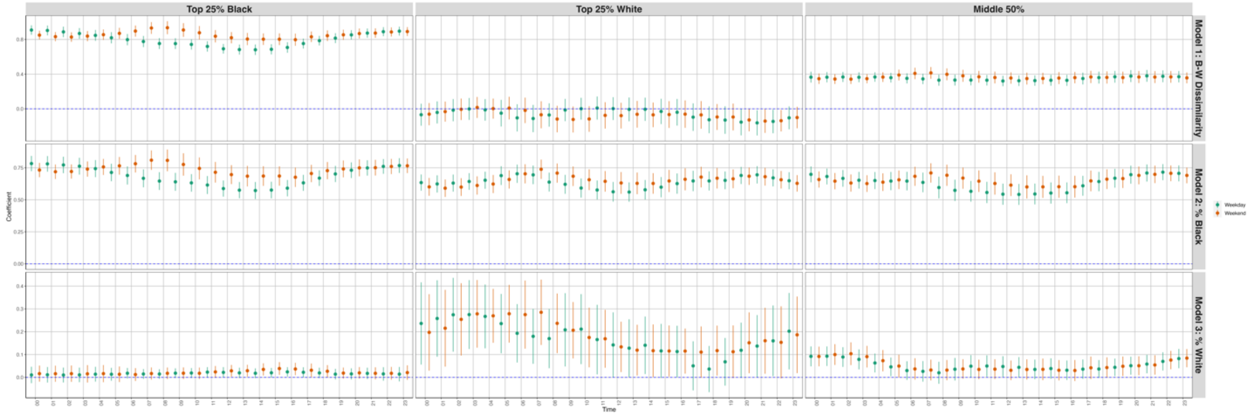

Models without public transportation stops as an independent variable, with three additional metropolitan areas (Louisville, KY; New Orleans, LA; and San Antonio, TX)

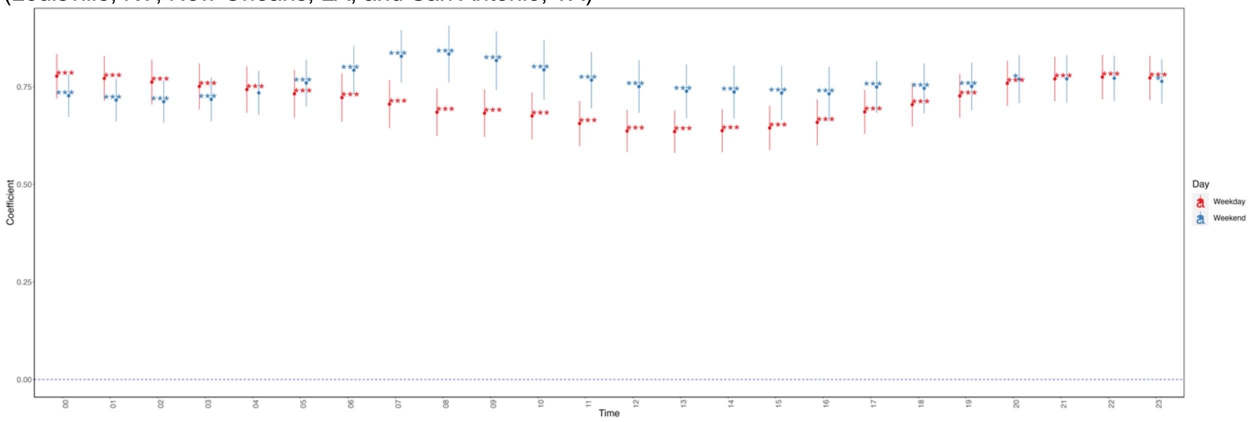

% White Residents by Census Tract

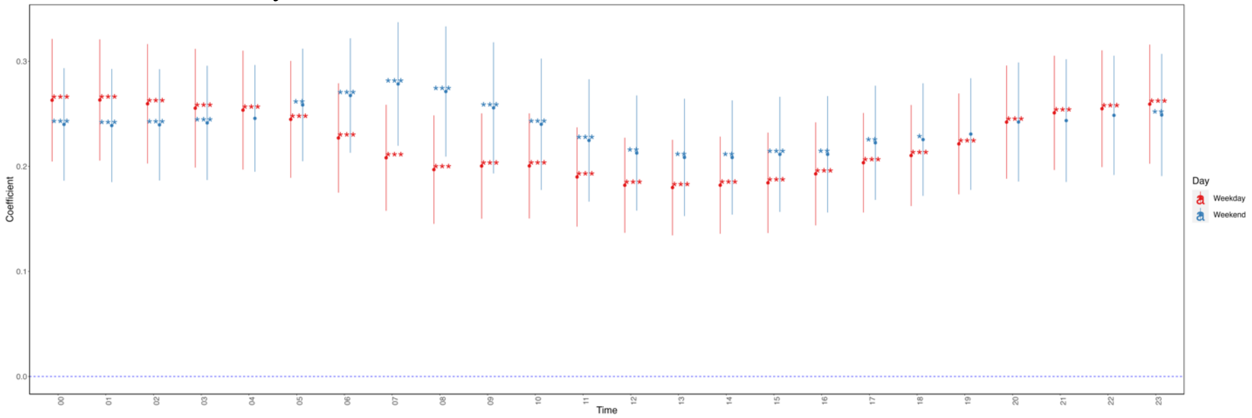

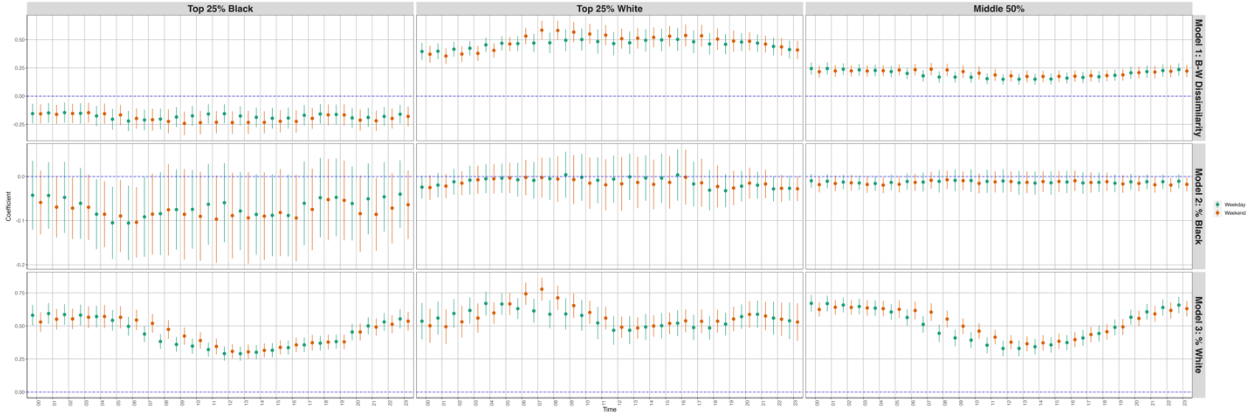

Models without public transportation stops as an independent variable, with three additional metropolitan areas (Louisville, KY; New Orleans, LA; and San Antonio, TX)

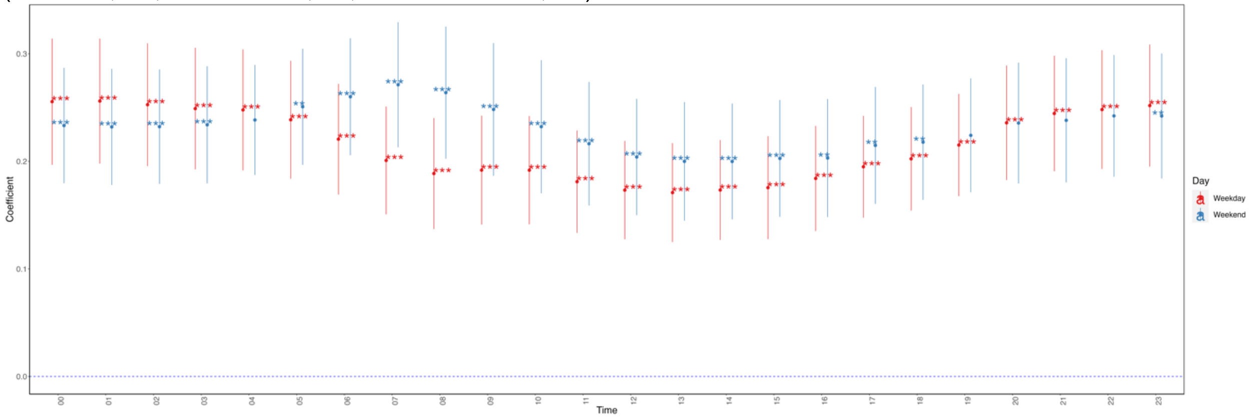

Professional, Scientific, and Technical Services

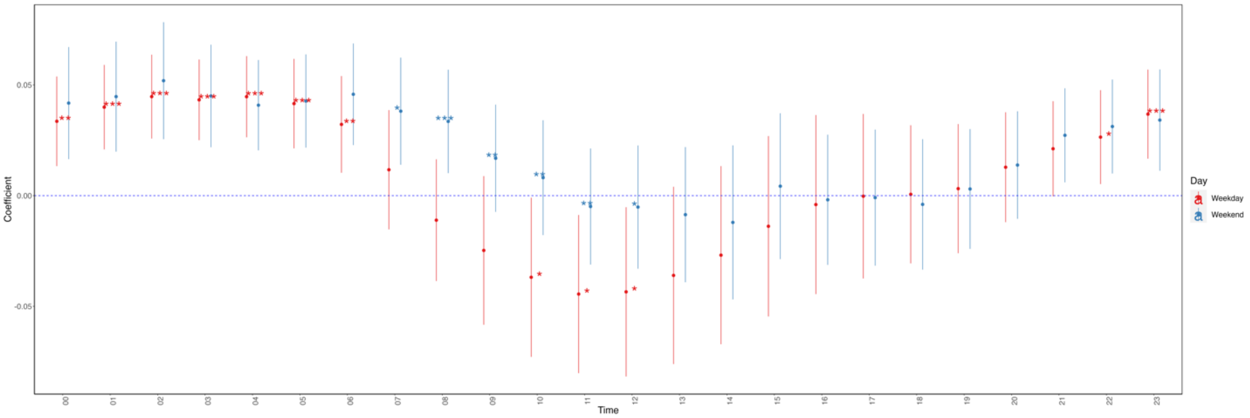

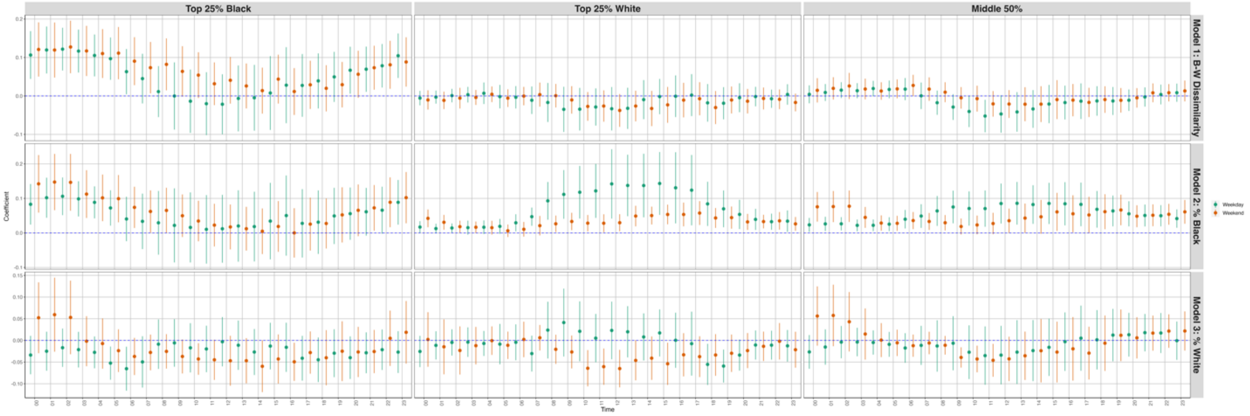

Models without public transportation stops as an independent variable, with three additional metropolitan areas (Louisville, KY; New Orleans, LA; and San Antonio, TX)

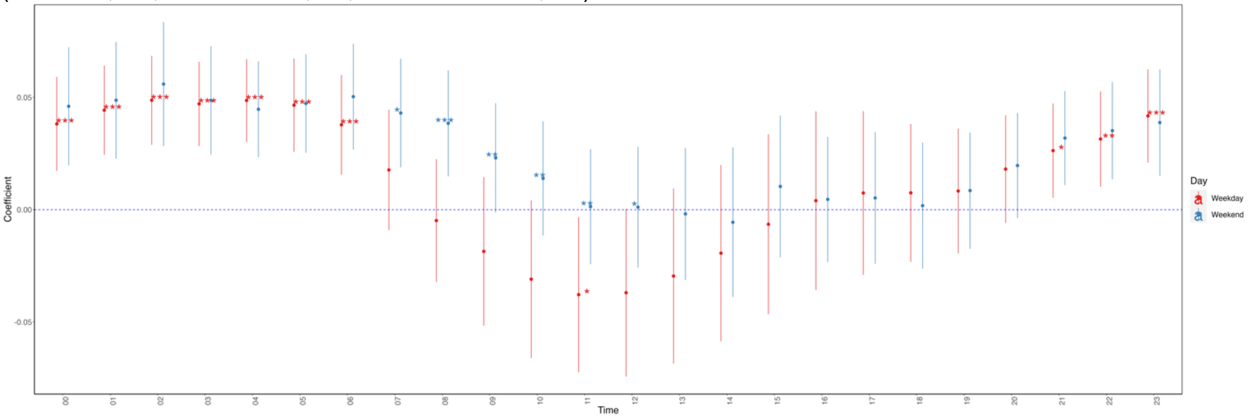

Public Administration

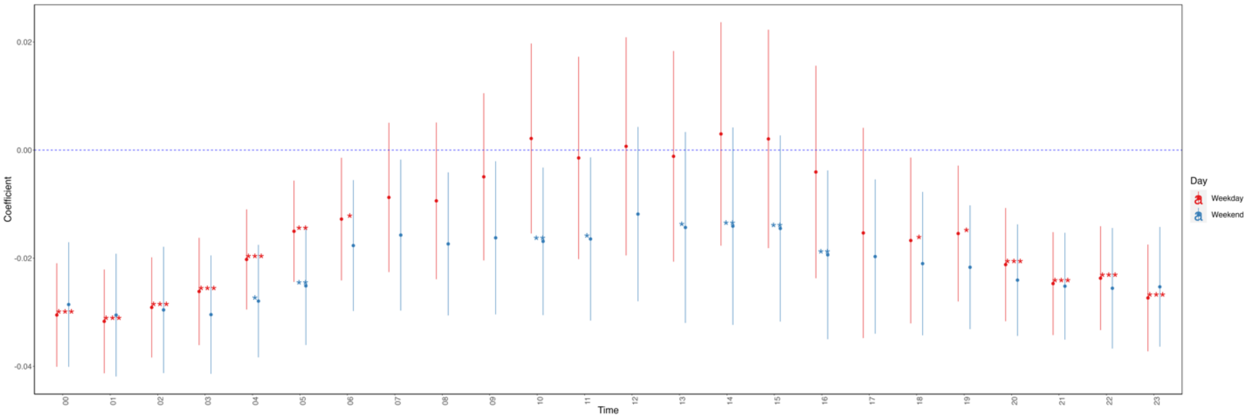

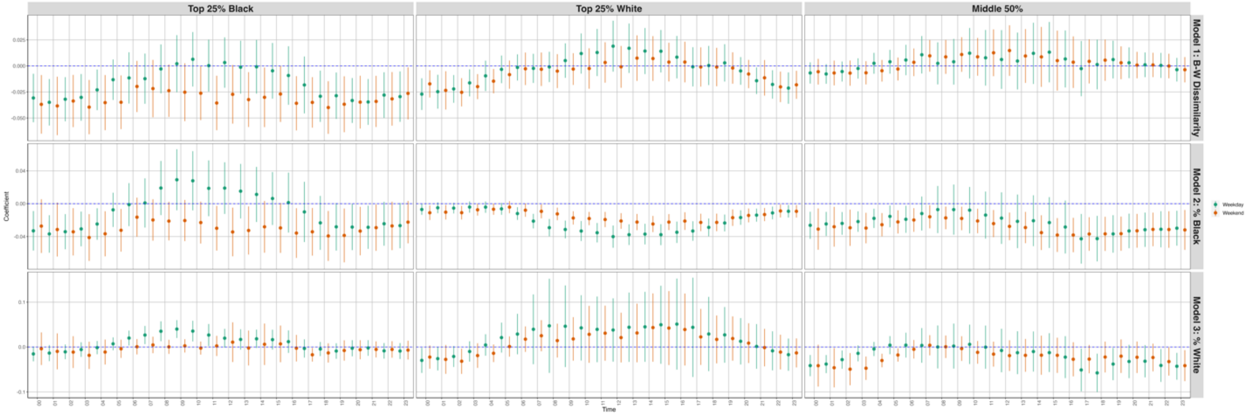

Models without public transportation stops as an independent variable, with three additional metropolitan areas (Louisville, KY; New Orleans, LA; and San Antonio, TX)

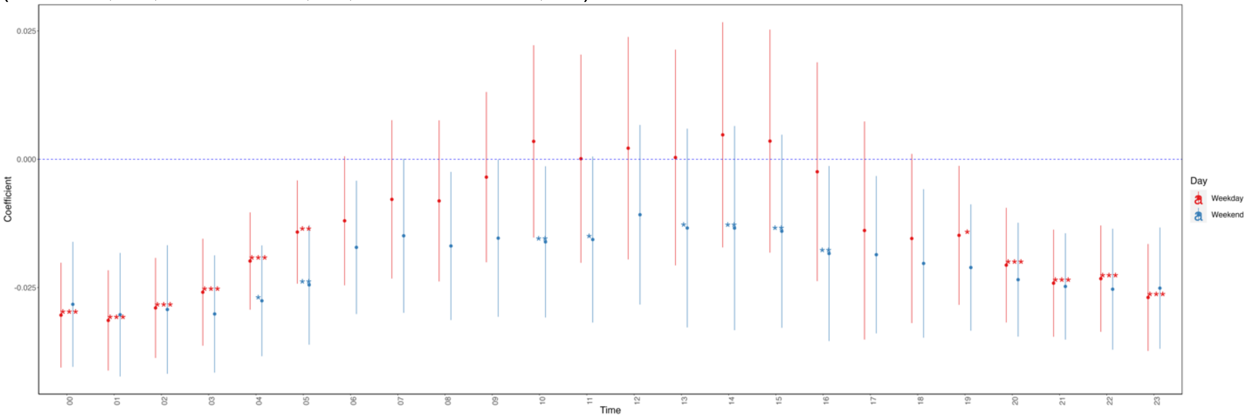

Real Estate, Rental, and Leasing

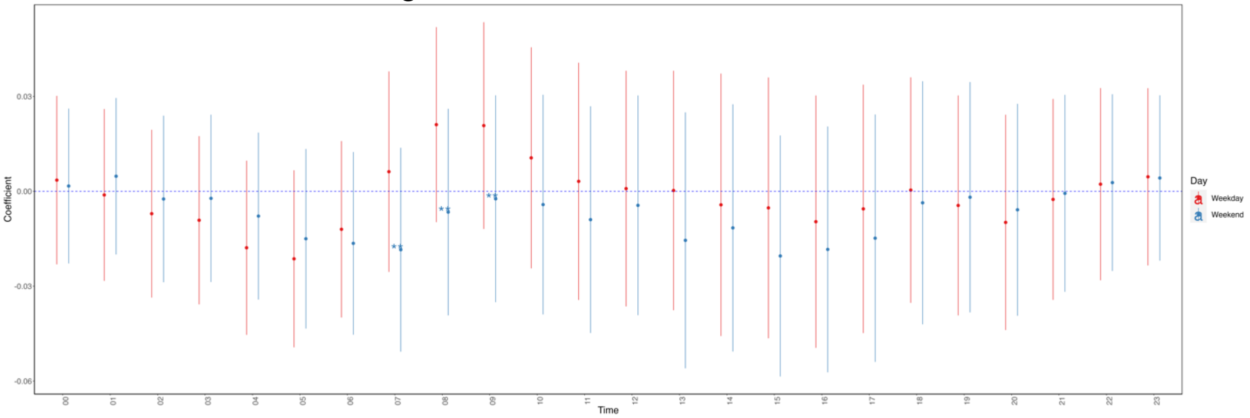

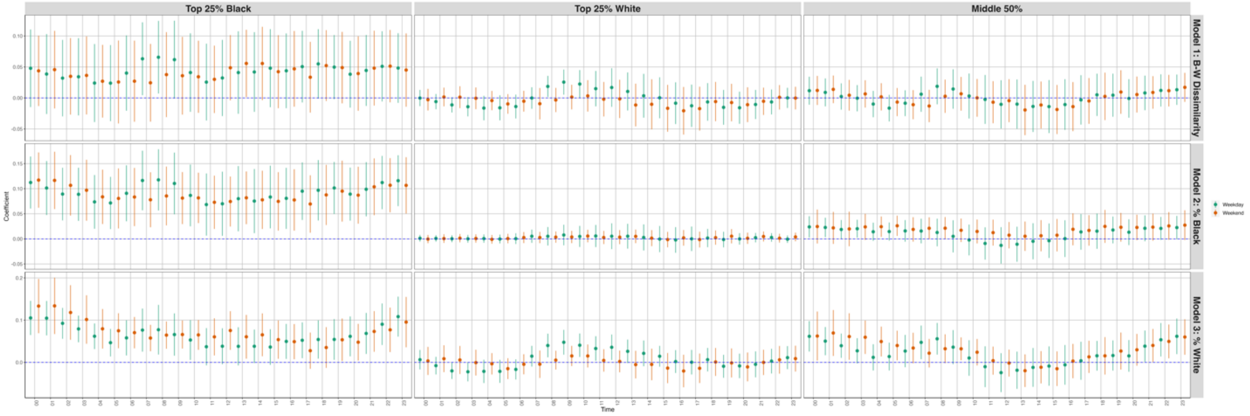

Models without public transportation stops as an independent variable, with three additional metropolitan areas (Louisville, KY; New Orleans, LA; and San Antonio, TX)

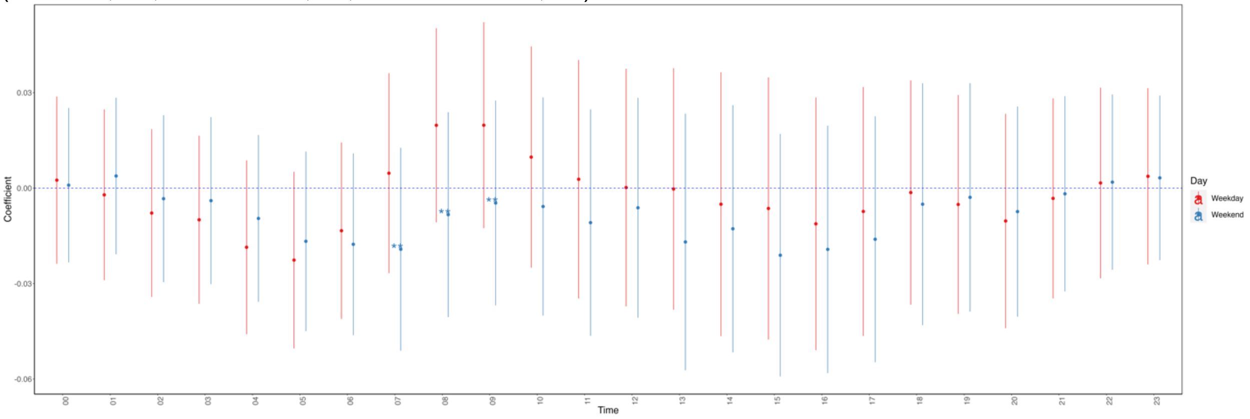

Recreation

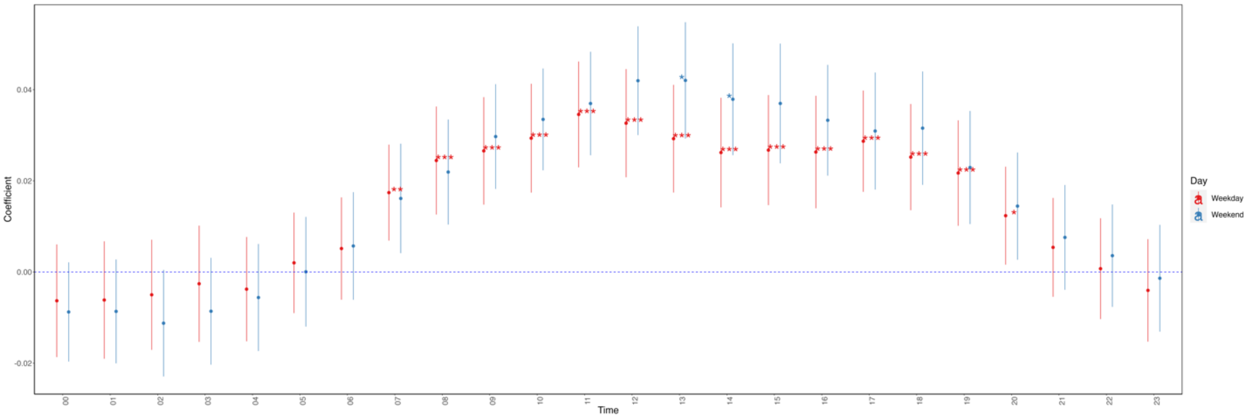

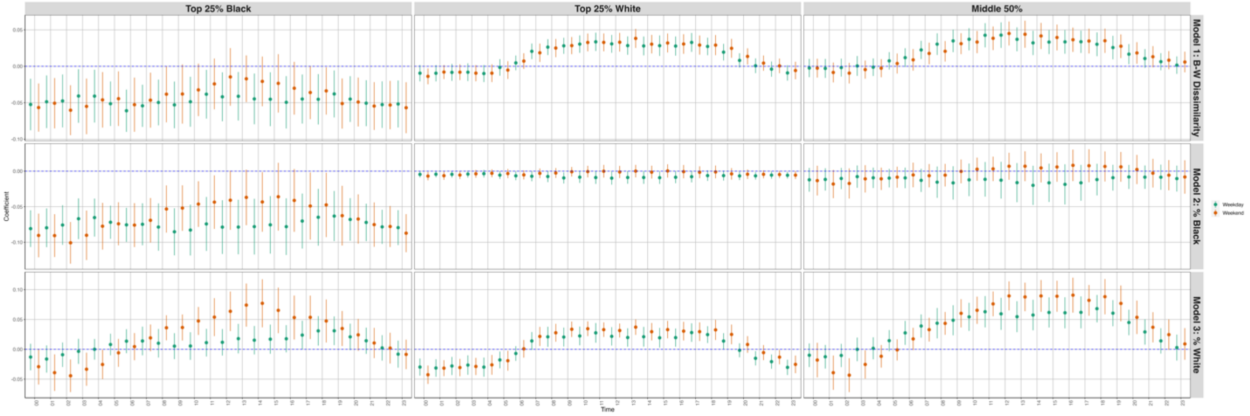

Models without public transportation stops as an independent variable, with three additional metropolitan areas (Louisville, KY; New Orleans, LA; and San Antonio, TX)

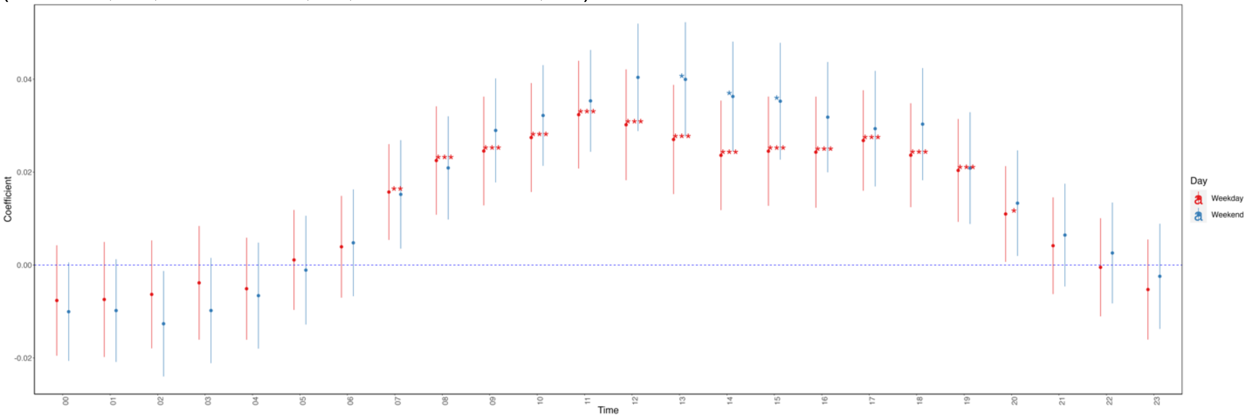

Religious Organizations

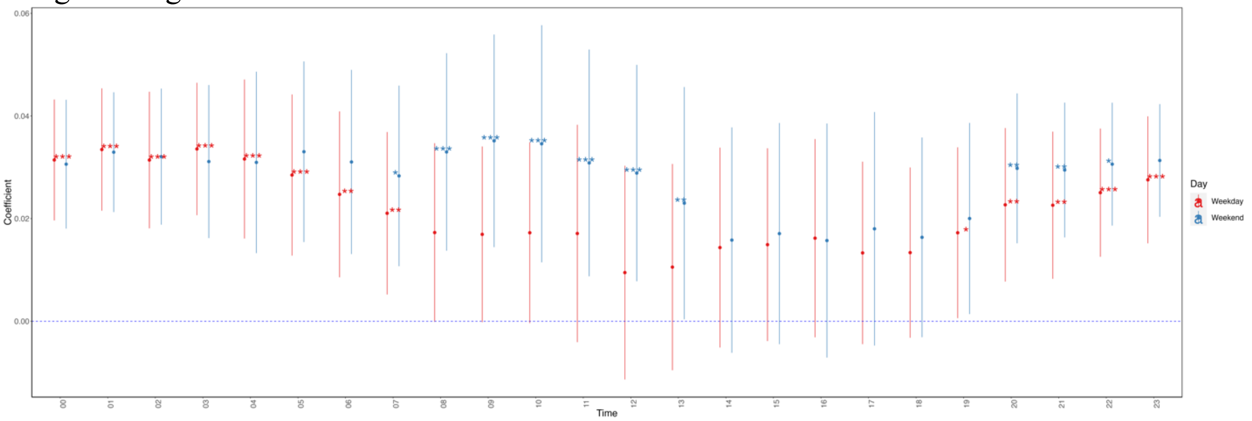

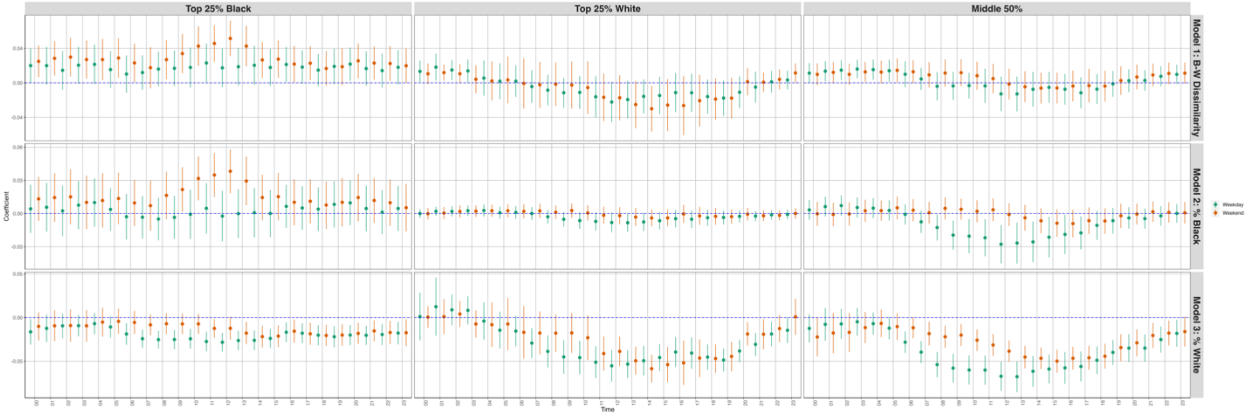

Models without public transportation stops as an independent variable, with three additional metropolitan areas (Louisville, KY; New Orleans, LA; and San Antonio, TX)

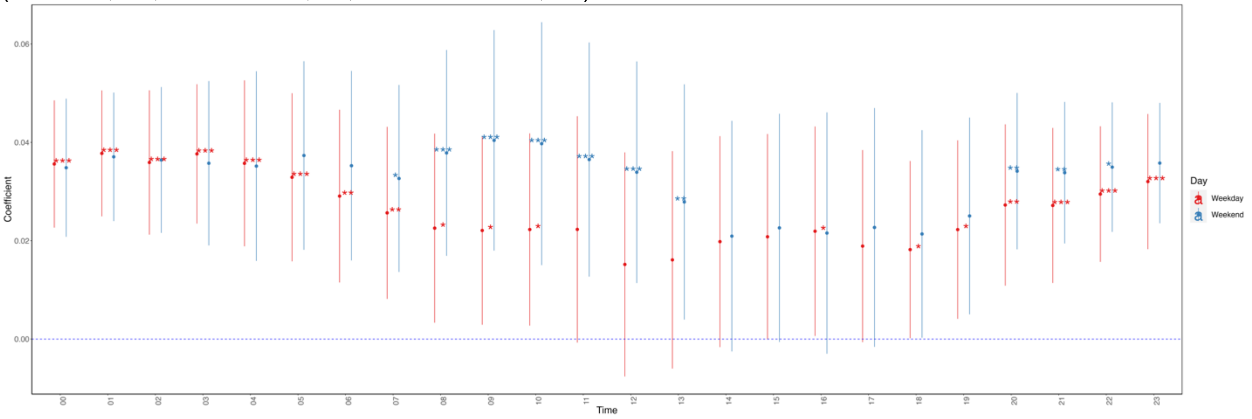

Restaurants and Bars

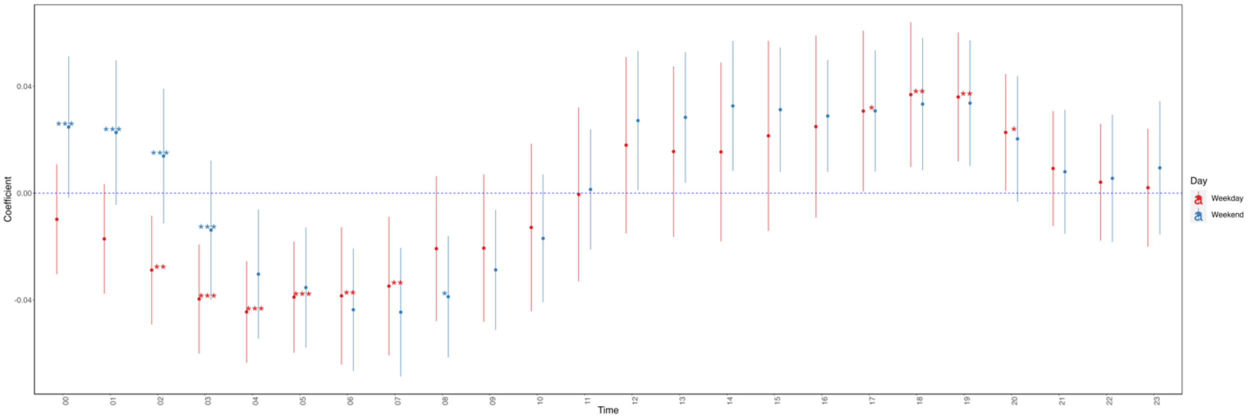

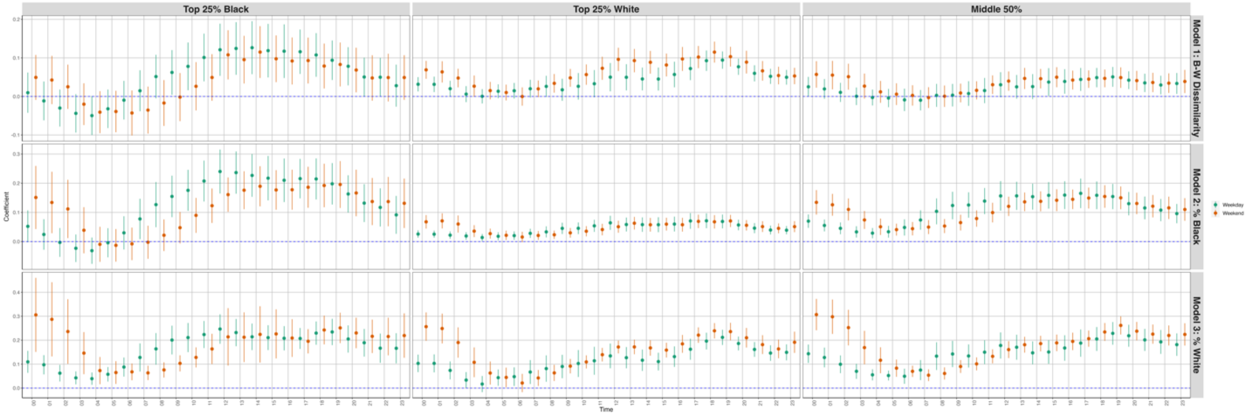

Models without public transportation stops as an independent variable, with three additional metropolitan areas (Louisville, KY; New Orleans, LA; and San Antonio, TX)

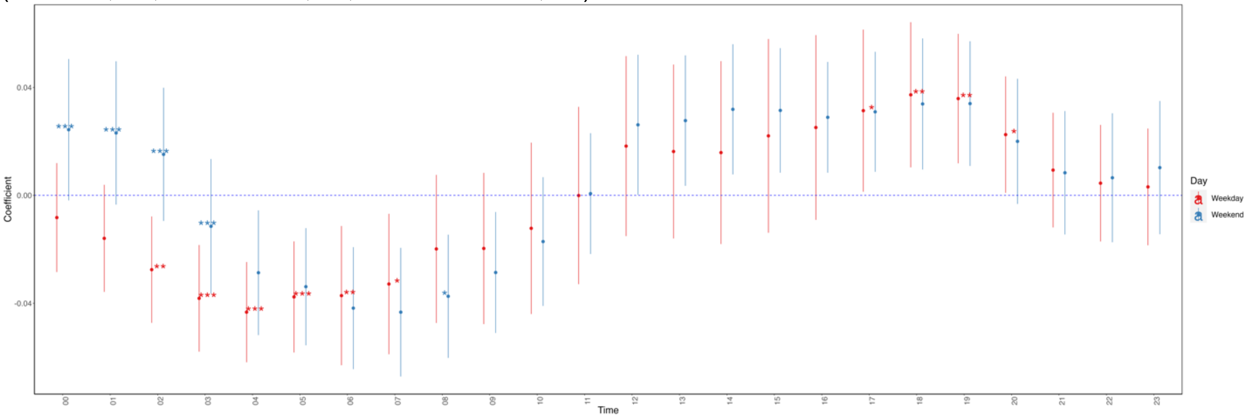

Residential Care

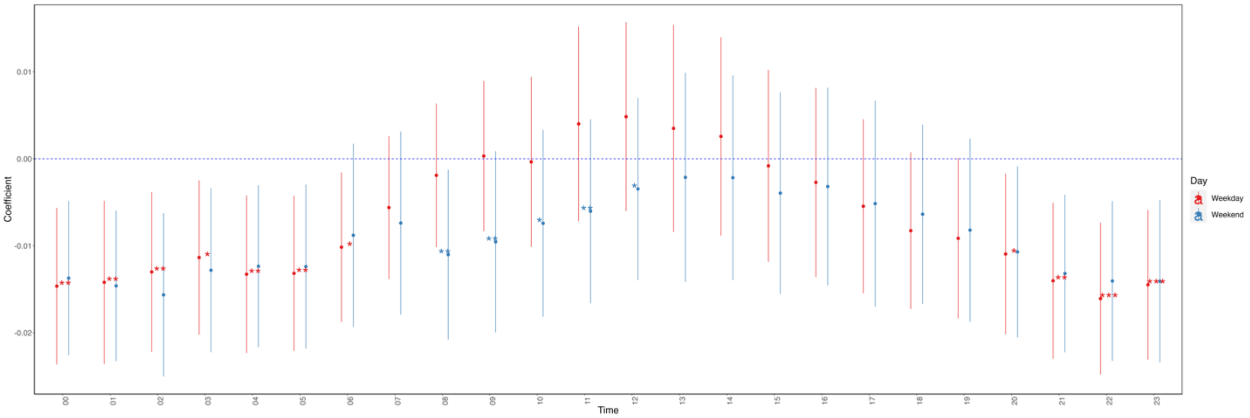

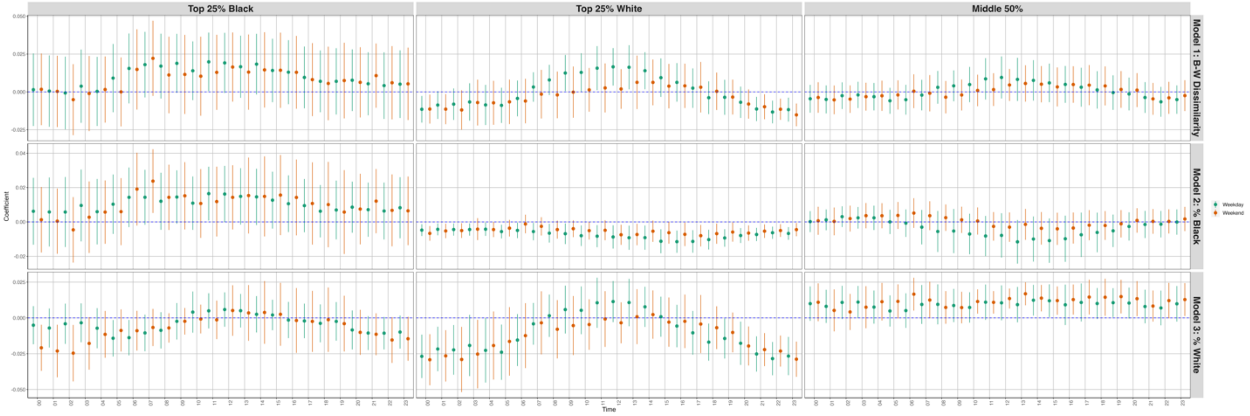

Models without public transportation stops as an independent variable, with three additional metropolitan areas (Louisville, KY; New Orleans, LA; and San Antonio, TX)

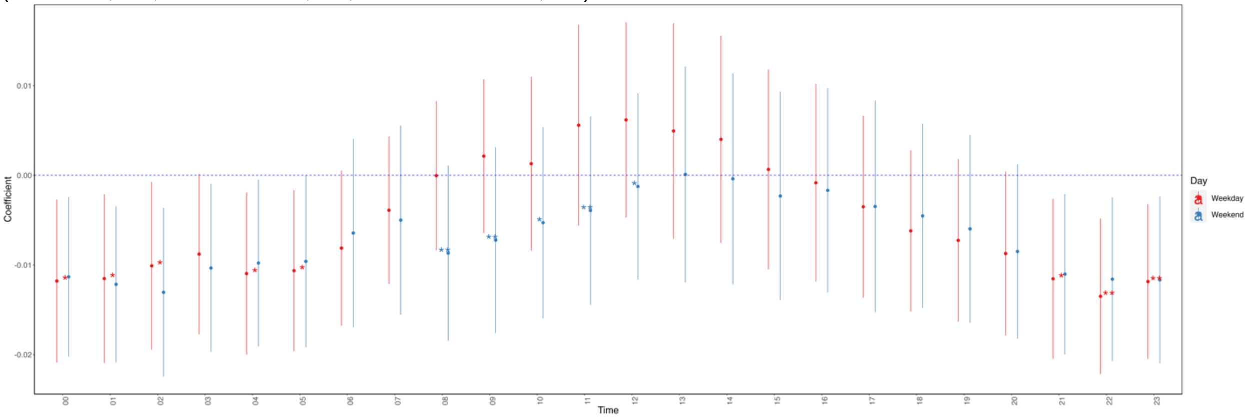

Retail

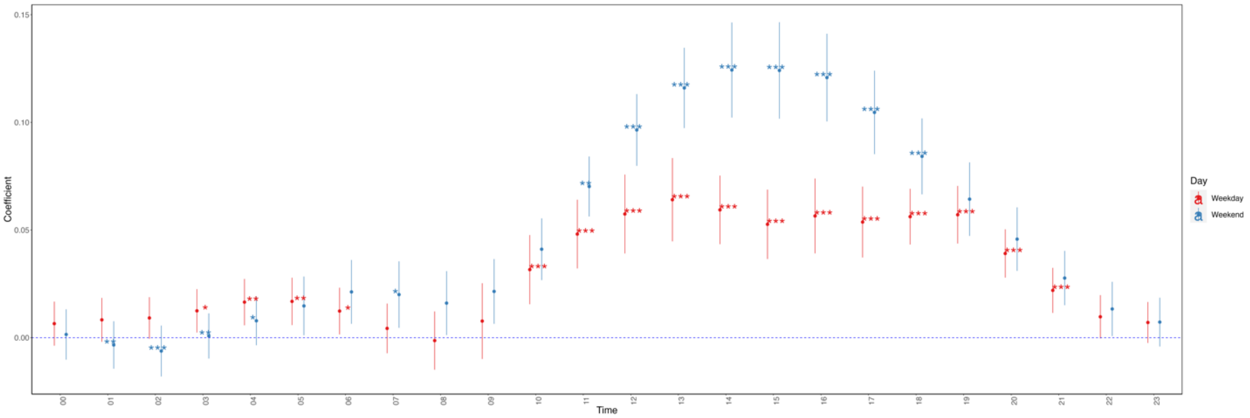

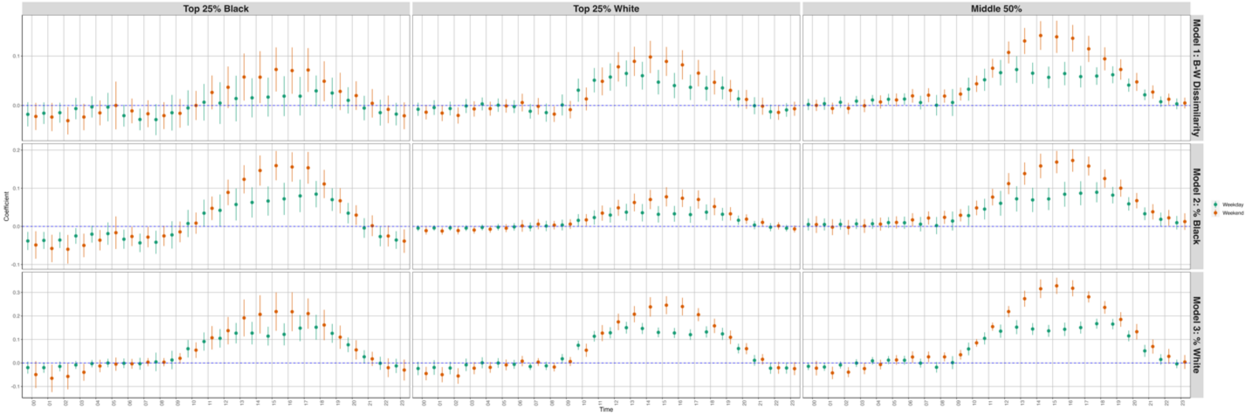

Models without public transportation stops as an independent variable, with three additional metropolitan areas (Louisville, KY; New Orleans, LA; and San Antonio, TX)

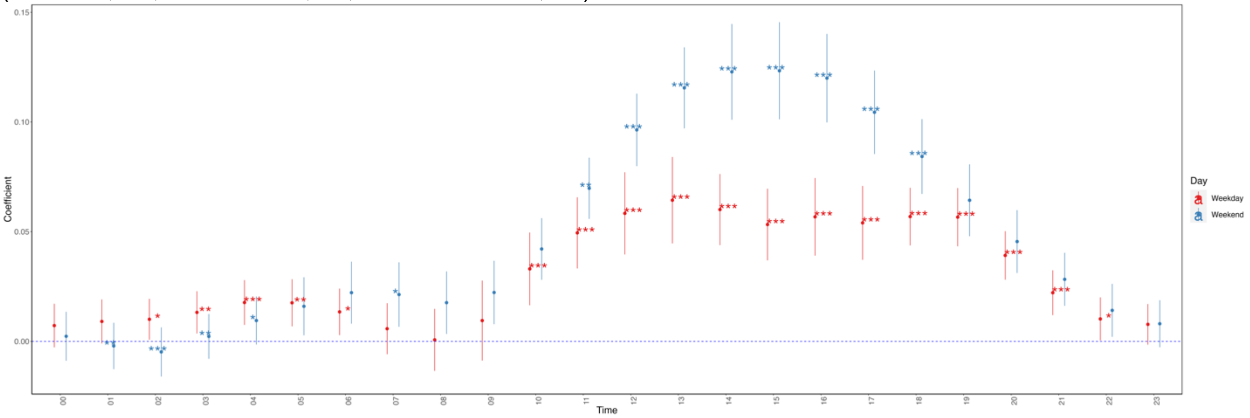

Census Tract Population Size

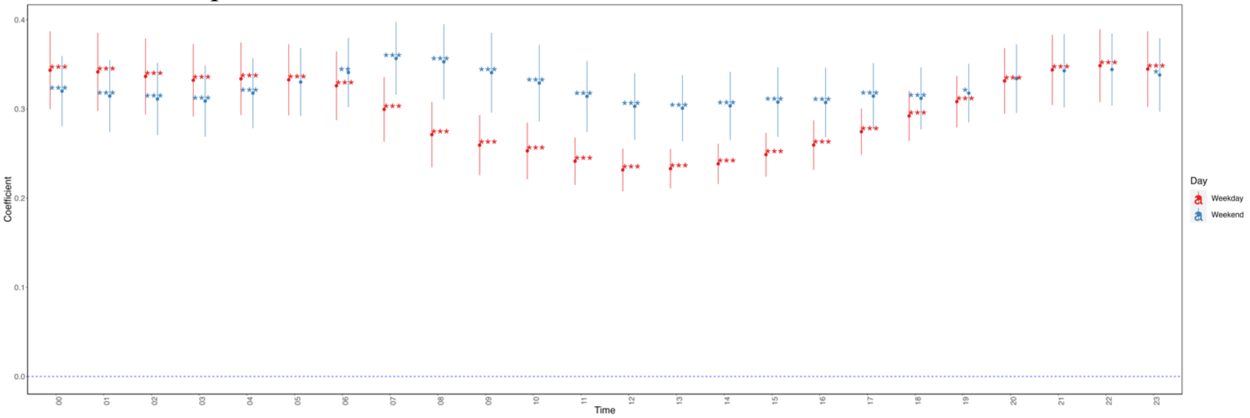

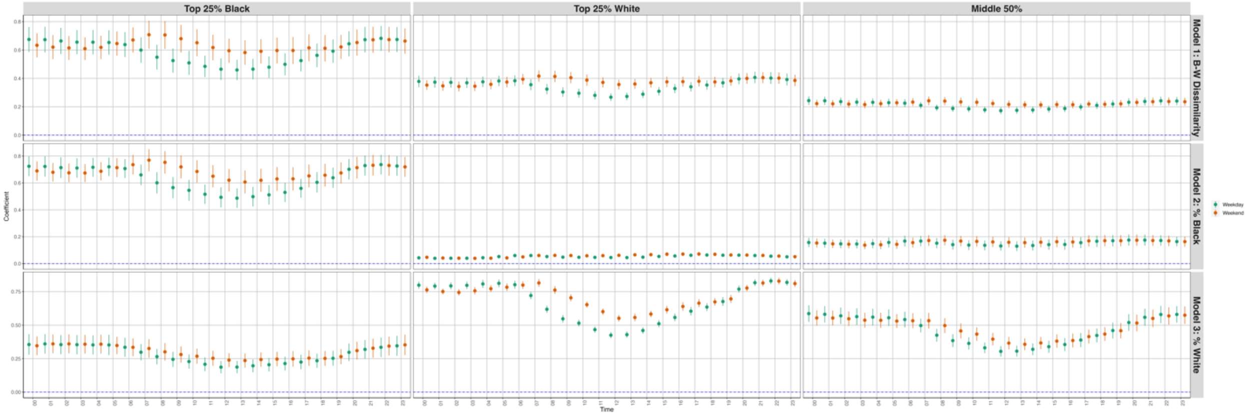

Models without public transportation stops as an independent variable, with three additional metropolitan areas (Louisville, KY; New Orleans, LA; and San Antonio, TX)

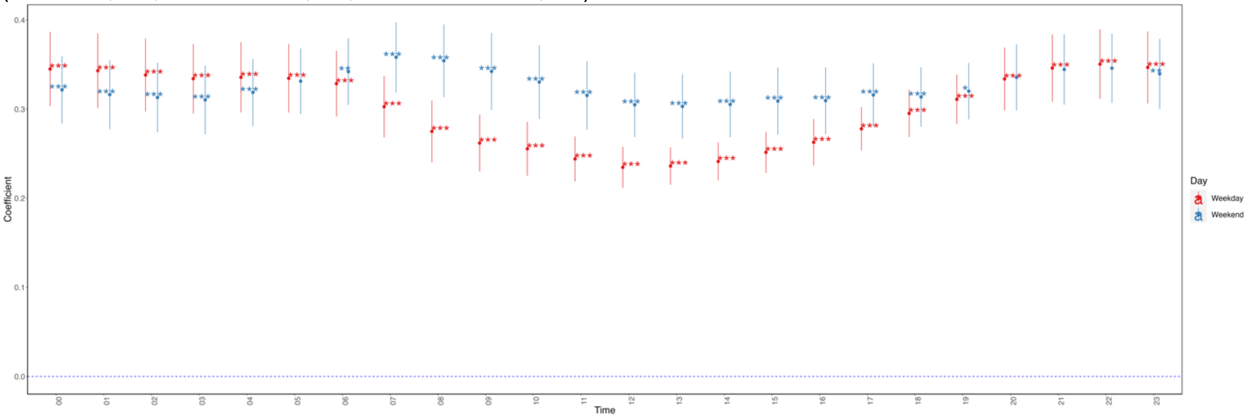

Social Assistance Organizations

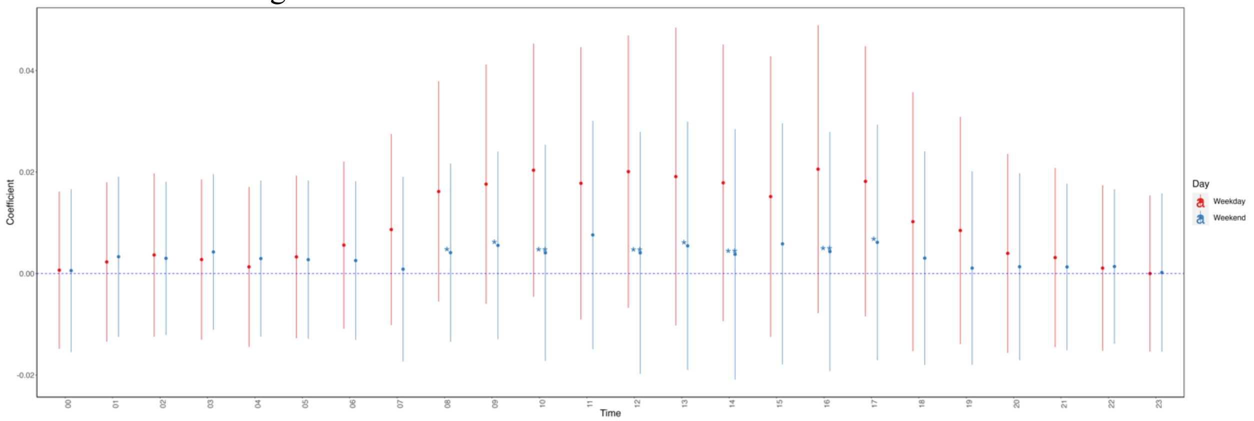

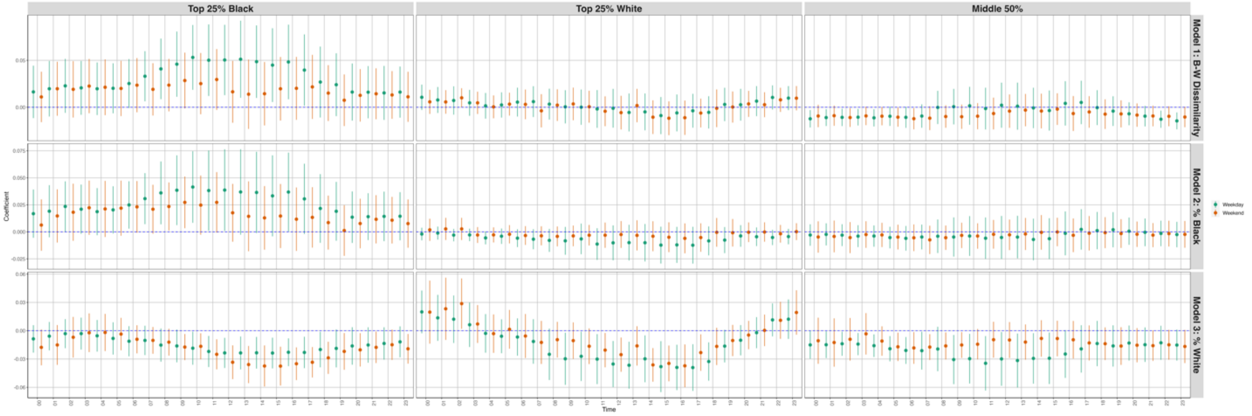

Models without public transportation stops as an independent variable, with three additional metropolitan areas (Louisville, KY; New Orleans, LA; and San Antonio, TX)

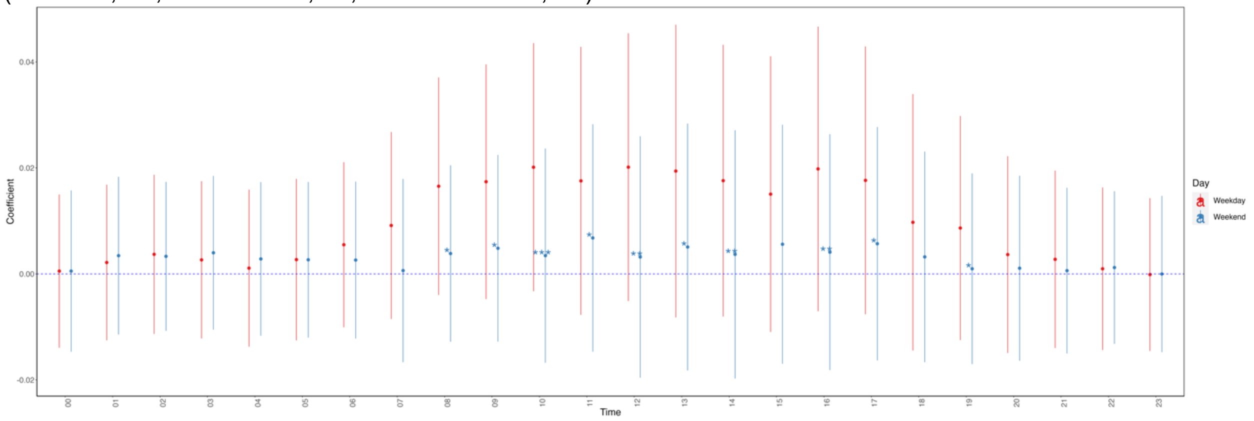

% Visits to Home Census Tracts

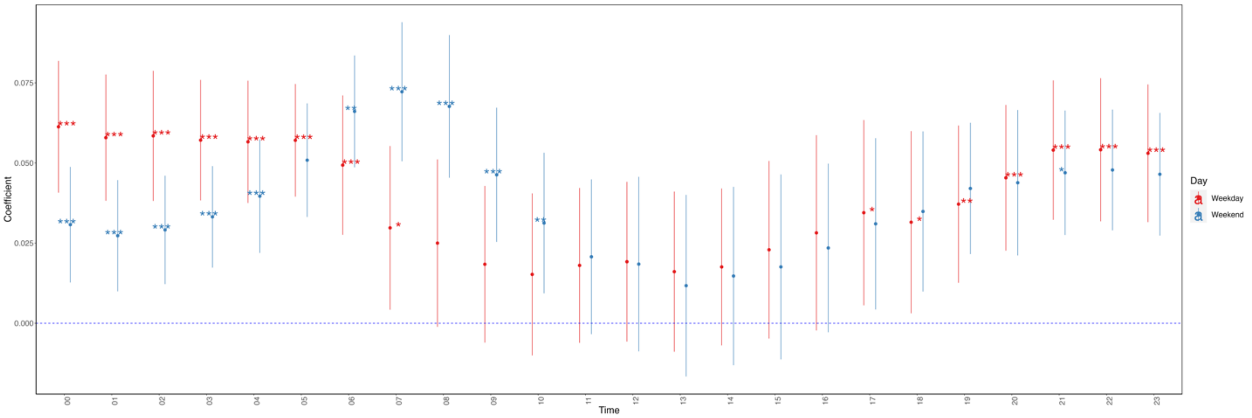

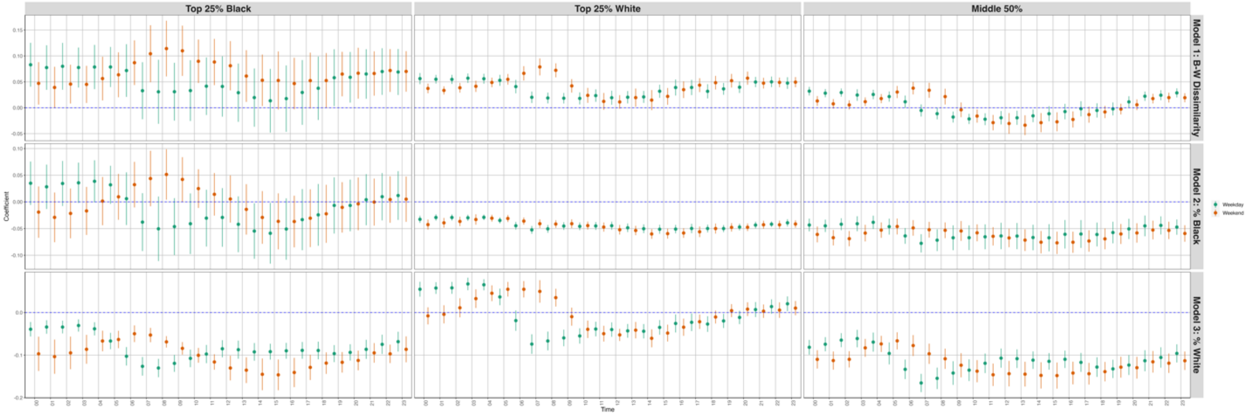

Models without public transportation stops as an independent variable, with three additional metropolitan areas (Louisville, KY; New Orleans, LA; and San Antonio, TX)

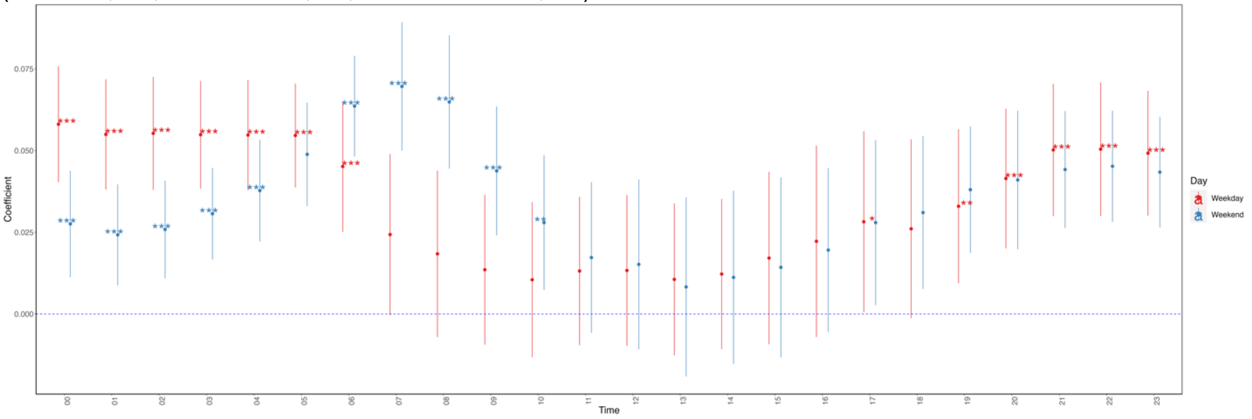

Number of Public Transportation Stops

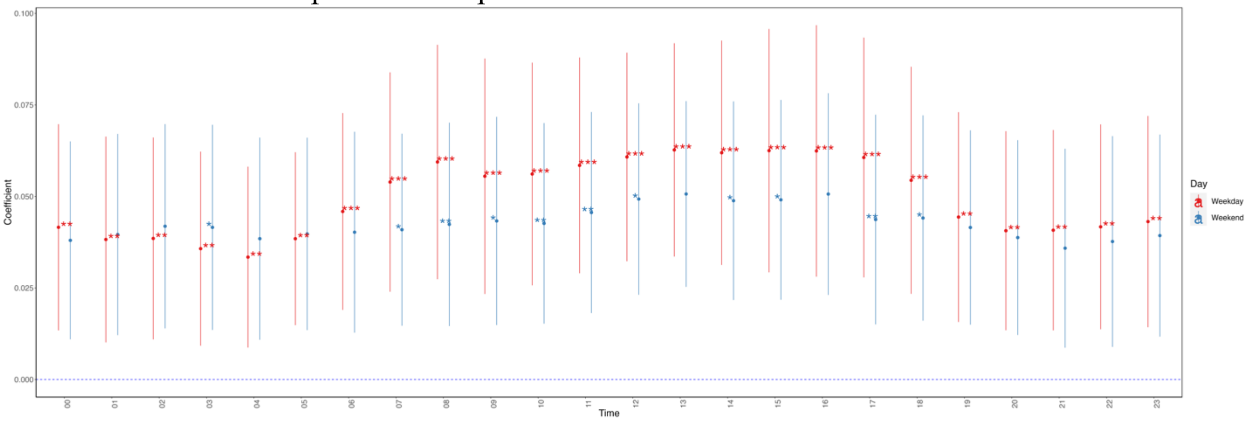

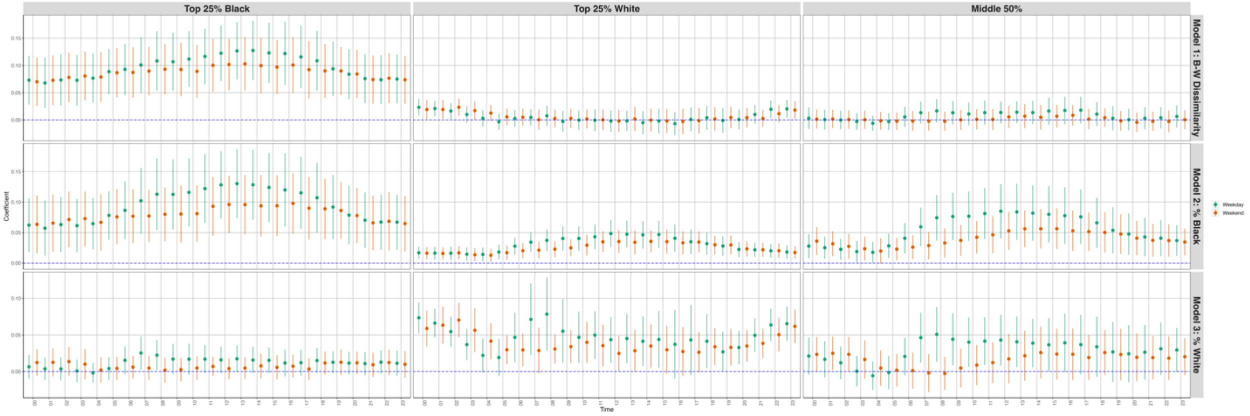

Transportation and Warehousing

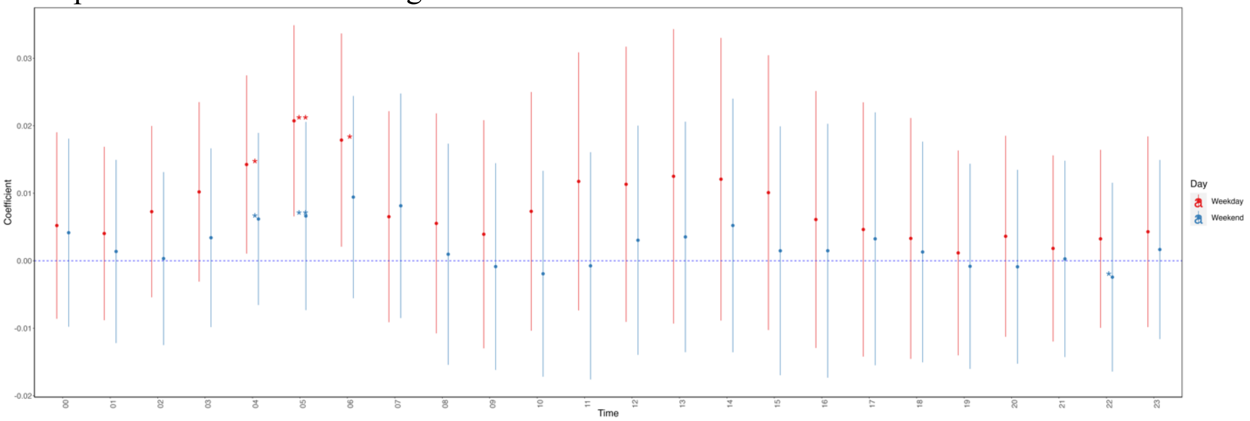

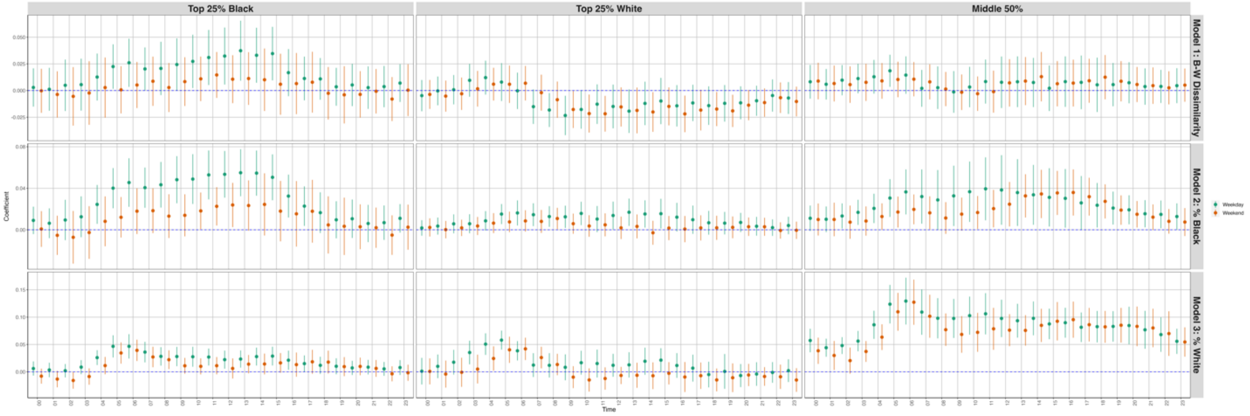

Models without public transportation stops as an independent variable, with three additional metropolitan areas (Louisville, KY; New Orleans, LA; and San Antonio, TX)

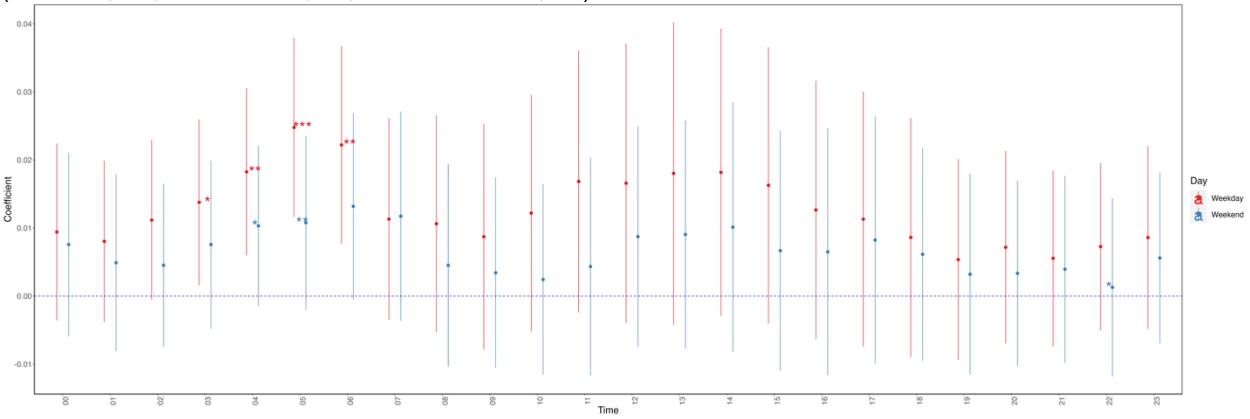

Utilities

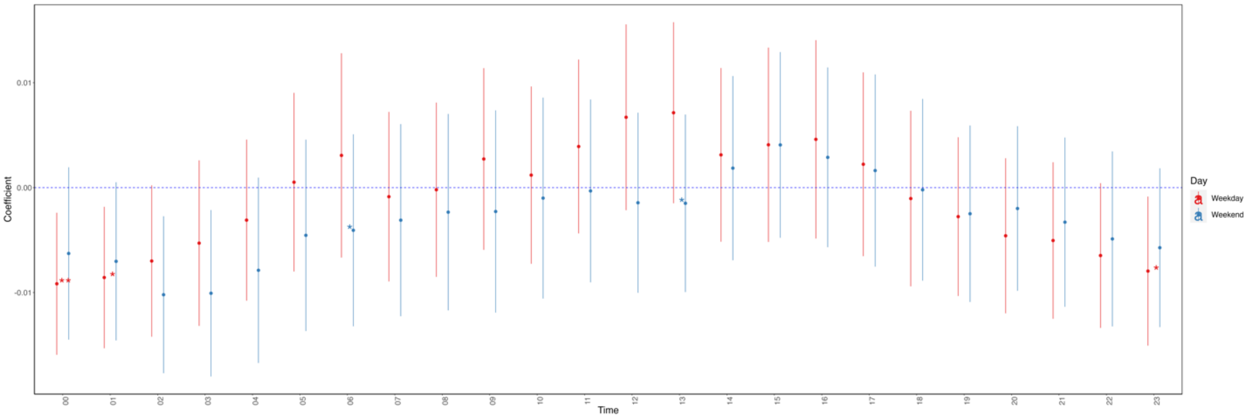

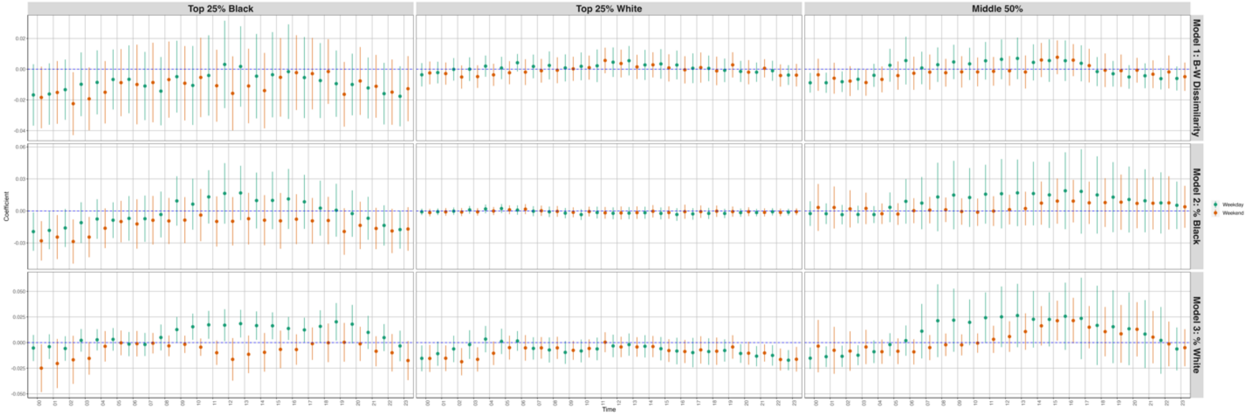

Models without public transportation stops as an independent variable, with three additional metropolitan areas (Louisville, KY; New Orleans, LA; and San Antonio, TX)

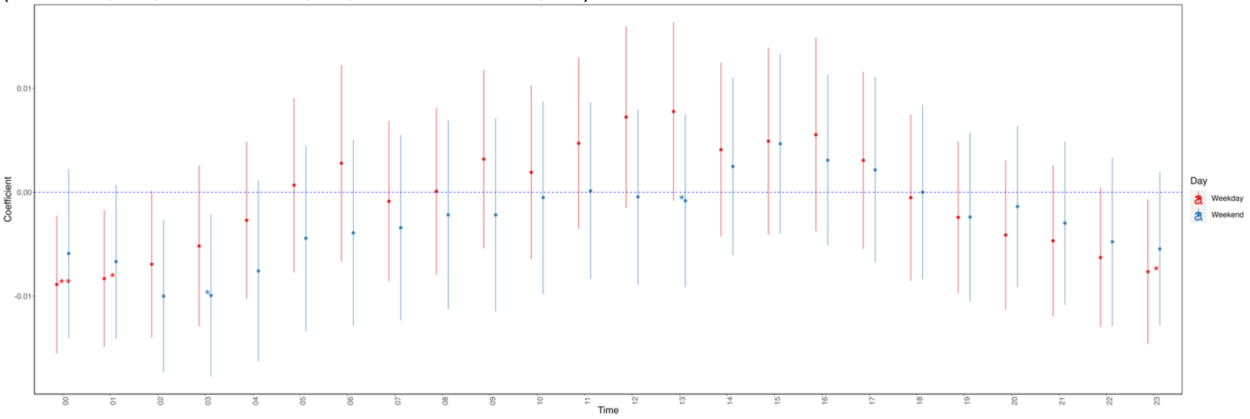

Wholesale Trade

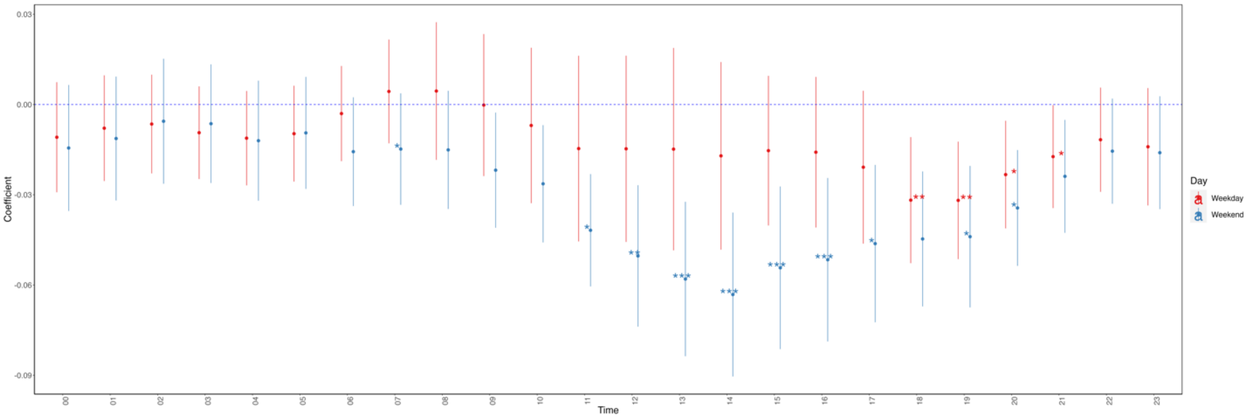

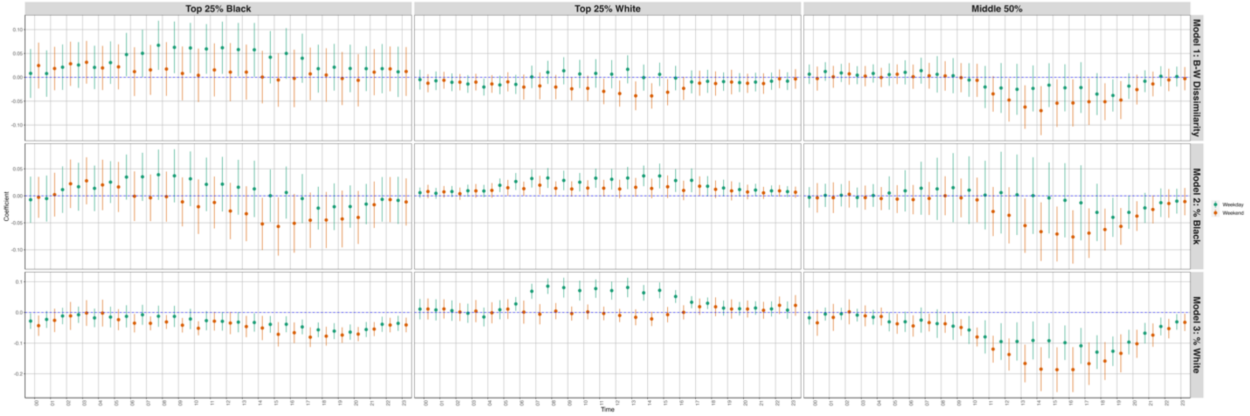

Models without public transportation stops as an independent variable, with three additional metropolitan areas (Louisville, KY; New Orleans, LA; and San Antonio, TX)

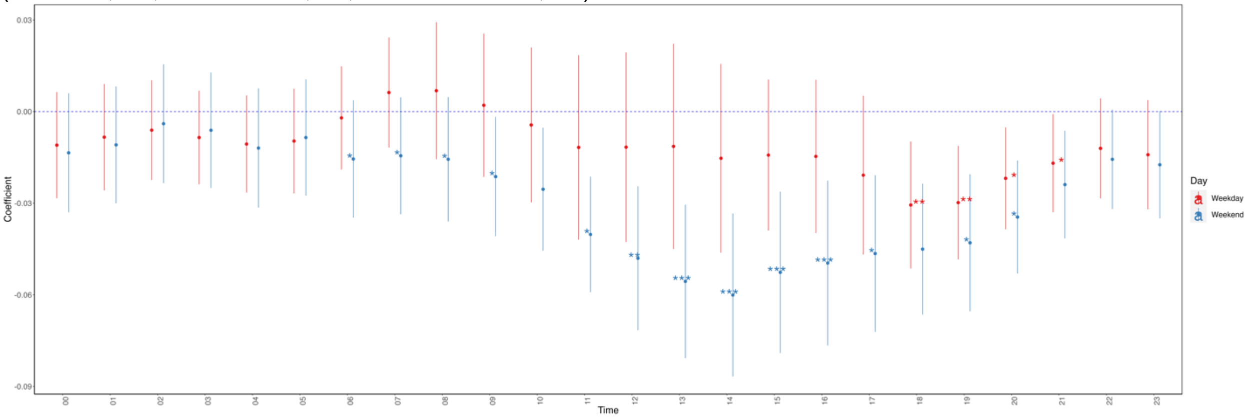

Supplement: Supplementary file 1 — Supplementary Information. [file 41598_2024_56257_MOESM1_ESM.pdf]
